# Primary care experiences at the intersection of sexual minority status and long-term mental health conditions in England: a cross-sectional analysis using the English General Practice Patient Survey

**DOI:** 10.1016/j.eclinm.2026.104016

**Published:** 2026-06-11

**Authors:** Arthur Gomes-Mendes, Sally McManus, Els van der Ven, Danai Dima, Anne-Kathrin Fett

**Affiliations:** a Centre for Clinical, Social & Cognitive Neuroscience, Department of Psychology and Neuroscience, School of Health and Medical Sciences, City St George's, University of London, London, UK; b Violence and Society Centre, City St George's, University of London, London, UK; c Faculty of Behavioural and Movement Sciences, Clinical Psychology, Vrije Universiteit Amsterdam, Amsterdam, the Netherlands; d Department of Neuroimaging, Institute of Psychiatry, Psychology and Neuroscience, King's College London, London, UK; e Department of Psychosis Studies, Institute of Psychiatry, Psychology and Neuroscience, King's College London, London, UK

**Keywords:** Public health, Primary care, Sexual minority orientation, Long-term mental health conditions, Intersectionality, England

## Abstract

**Background:**

Sexual minorities and individuals with mental health conditions (MHC) often report poorer healthcare experiences, but evidence on inequalities at their intersection remains limited.

**Methods:**

We analysed data from 1,277,383 respondents to the 2022–23 English General Practice Patient Survey using survey-weighted logistic regressions. Sexual orientation and self-reported long-term MHC were fully interacted to estimate adjusted predicted probabilities for six indicators of primary care experience: recognition of mental health needs, confidence and trust in healthcare professionals (HCP), HCP interpersonal skills, involvement in care decisions, overall needs met, and help-seeking before appointments.

**Findings:**

Sexual minorities with MHC accounted for 1.2% of our analytical sample and reported the lowest satisfaction with involvement in care decisions (89.8% [89.3–90.2]), and HCP interpersonal skills (8.9% [8.4–9.3]) compared to all other groups. Interaction effects indicated partially protective patterns among sexual minorities with MHC for recognition of mental health needs (81.7% [80.6–82.7]), confidence/trust in HCP (93.3% [92.8–93.9]), and overall needs met (91.3% [90.7–91.9]), with the interaction effects corresponding to positive departures from additivity of 0.8 to 2.1 percentage-points. For these outcomes, sexual minorities with MHC exhibited moderate levels of positive primary-care experience, falling between the highest- and lowest-positive endorsement groups.

**Interpretation:**

Sexual minority orientation and long-term MHC independently related to lower primary care satisfaction. However, their intersection was associated with both disadvantage and relative protection across different domains. Recognising these patterns is essential for reducing inequalities and better tailoring primary care services.

**Funding:**

This article was not commissioned or funded by any external agency.

**Research in context**

**Evidence before this study**

Sexual minority individuals and people living with mental health conditions consistently report poorer healthcare experiences than their heterosexual or mentally healthy counterparts. To examine how these characteristics intersect in shaping primary care experiences, we searched PubMed, EMBASE, and Scopus from database inception to Aug 27, 2025, with no language restrictions, using the following search terms: (lgb OR lesbian OR gay OR bisexual OR queer OR homosexual OR sexual minority OR nonheterosexual OR non-heterosexual) AND (psychological distress OR mental distress OR psychological problems OR psychological disorders OR mental health problems OR mental disorders OR mental health disorders OR mental health issues OR mental illness OR mental health conditions OR common mental health disorders OR mental health challenges OR chronic mental health issues) AND (intersectional framework OR intersectionality OR intersecting identities OR multiple stigma OR dual stigma OR double jeopardy OR compounded disadvantage OR cumulative disadvantage OR marginalisation OR marginalization OR multiple minority status) AND (United Kingdom OR UK OR Great Britain OR England OR English people OR Scotland OR Wales OR Welsh OR Northern Ireland OR British).

After the removal of duplicates, 48 records were identified. Of these, 26 addressed intersectionality, most often focusing on combinations of sexual orientation with ethnicity, socioeconomic status, or gender, and typically treating mental health as an outcome rather than an exposure. Only one narrative review briefly discussed sexual orientation and mental health status as parallel stigmatised characteristics, without empirical examination of their joint association with healthcare experiences. Although both sexual minority status and mental health conditions have independently been linked to poorer experiences in primary care, robust population-based evidence on how these identities relate to satisfaction, trust, and help-seeking behaviour remains limited. This leaves open questions as to whether intersecting minority identities relate to compounded disadvantage, buffering effects, or more complex patterns of inequality in healthcare.

**Added value of this study**

Using nationally representative data from 1,277,383 respondents to the 2022–23 English General Practice Patient Survey, we provide novel evidence on how sexual orientation and long-term mental health conditions intersect to shape primary care experiences in England. By fully interacting these identities in survey-weighted logistic regression models, we estimated adjusted predicted probabilities for six indicators of primary care experience, including recognition of mental health needs, confidence and trust in healthcare professionals, healthcare professional's interpersonal skills, involvement in care decisions, overall needs met, and help-seeking before GP appointments.

Overall differences in primary care experiences were modest, and satisfaction with NHS primary care was high, with positive endorsement ranging from 62.5% to 94.3%. However, sexual minorities, individuals with a long-term mental health condition, and particularly sexual minority individuals with a mental health condition consistently reported lower probabilities of positive experiences than heterosexual individuals without a mental health condition. Sexual minority individuals with a mental health condition showed the lowest satisfaction with involvement in care decisions and the most negative evaluations of the healthcare professional's interpersonal skills. At the same time, for recognition of mental health needs, confidence and trust in the healthcare professional, and overall needs met, sexual minority individuals with a mental health condition had intermediate probabilities of positive endorsement, with the interaction between sexual orientation and mental health condition accounting for increases of 0.75–2.13 percentage points above what would be expected under additivity. This suggests that intersectionality can operate as a potential buffer in specific dimensions of care.

In addition, sexual minority individuals with a mental health condition were the most likely group to seek support prior to attempting to schedule a GP appointment, particularly through informal sources. This pattern may reflect dissatisfaction with formal care, but also greater mental health literacy, awareness, and proactive help-seeking, which have previously been observed among both sexual minorities and individuals with mental health conditions.

**Implications of all the available evidence**

Our findings address a critical gap in the literature by demonstrating that sexual minority orientation and the presence of a long-term mental health conditions intersect in complex ways related to primary care experiences. While both characteristics independently relate to lower satisfaction, we observed both negative and protective intersectional effects, for different outcomes. Taken together, these patterns suggest that intersecting identities can shape both vulnerabilities and opportunities for support within primary care, underscoring the need for interventions that recognise this complexity.

At the same time, lower satisfaction with interpersonal aspects of care and involvement in decision-making among sexual minorities and individuals with mental health conditions underscores the need for continued efforts to improve inclusivity, communication, and patient-centred care in primary care settings. Further research is needed to investigate the mechanisms underlying the observed effects, assess generalisability across sexual minority subgroups, and examine how the type, severity, and duration of mental health conditions moderate these patterns. Such work is essential to informing targeted interventions aimed at reducing healthcare inequalities and improving care for individuals at the intersection of multiple minoritised identities.

## Introduction

Estimates suggest that between one in twenty to one in nine^1^^,^^2^ adults in the UK identify as a sexual minority, i.e. gay or lesbian, bisexual, or other non-heterosexual orientation. Sexual minorities have a higher risk for mental disorders than heterosexual individuals both globally and locally,^3^ with UK cohort studies reporting 1.4–2.0 times higher odds.^4^ This is in line with the benchmark risk observed for other global north, high-income countries, where two-to three-fold higher odds persist even after controlling for country-level sexual minority acceptance.^3^

Although the mechanisms underlying this elevated risk are not yet fully understood, Minority Stress Theory^5^ suggests that this elevated risk is driven by the accumulation of unique stressors linked to sexual minority orientation i.e., social exclusion, experiences of discrimination, perceived, enacted and internalised stigma, contributing to sustained psychological distress and, in turn, poorer mental health among non-heterosexual individuals.^4^

Alongside mental health inequalities, sexual minority individuals report more negative healthcare interactions,^6^ greater unmet care needs,^7^ and poorer mental health treatment outcomes.^8^ These inequalities may result from perceived and enacted discrimination and stigma,^6^^,^^9^ limited healthcare professionals' cultural competence,^6^ disenfranchisement from care decision-making,^10^ and decreased trust in healthcare systems following prior negative experiences.^11^^,^^12^ In parallel, and regardless of sexual orientation, individuals with mental disorders, also report poorer healthcare experiences, including unmet care needs,^13^^,^^14^ inadequate support for managing their condition,^13^^,^^15^^,^^16^ and healthcare providers’ dismissiveness,^15^ when compared to individuals without mental health conditions.

Intersectionality theory posits that social identities do not operate independently; rather, they intersect, producing unique effects that cannot be explained by simply adding the effects of stigmatised identities.^17^ Although having a mental disorder is not usually conceptualised as a social identity in intersectionality theory, emerging work suggests that it functions as a stigmatised social position that intersects with other minoritised identities.^18^ Mental health disparities among sexual minorities in the UK have widened over the past decade.^19^ Consequently, the intersection of these characteristics represents an urgent area of investigation with clear implications for policy and practice. While research has examined how each characteristic independently relates to poorer healthcare experiences and outcomes,^6^, ^7^, ^8^, ^9^, ^10^, ^11^, ^12^, ^13^, ^14^, ^15^, ^16^, ^17^^,^^20^, ^21^, ^22^ their joint impact on primary care experiences remains underexplored.

Using data from the nationally representative English General Practice Patient Survey (GPPS) we sought to examine how sexual minority orientation, presence of a long-term mental health condition, and their intersection relate to primary care experiences in England. We focused on experiences known to influence healthcare engagement, treatment adherence, and health outcomes^7^^,^^12^^,^^23^^,^^24^ and explored how these indicators of healthcare experience quality relate to help-seeking prior to scheduling a GP appointment.

We hypothesised that sexual minority individuals with a long-term mental health condition would report the least positive primary care experiences, reflecting compounded disadvantage at the intersection of these characteristics and that heterosexual individuals without long-term mental health condition would report the most positive experiences.

## Methods

### Study design and participants

The cross-sectional English GPPS data were obtained through a data-sharing agreement with NHS England through Ipsos MORI. To ensure a sufficient sample of sexual minority individuals, especially at the intersection of self-reported mental health conditions, we combined the 2022 and 2023 GPPS datasets. Participants were sampled at practice level through repeated stratified random sampling by age, gender and postcode using Personal Demographics Service (PDS) records and were eligible if aged ≥16 years, had a valid NHS number, and were registered with a GP for at least 6 months. The GPPS was administered to patients in 17 languages, by paper or online. Response rates for 2022 and 2023 were respectively 29.1 and 28.6%, covering approximately 1.1% of all registered NHS patients. While this is a small fraction of the registered population, GP registration covers nearly all of the English population, and the GPPS uses calibration weights to account for the sampling design and reduce potential non-response bias.^25^ Full methodological details for each year are published elsewhere.^25^

### Ethics statement

The original GPPS data collection was approved by the Central Office for Research Ethics Committee (COREC) and conducted by Ipsos MORI on behalf of NHS England. Detailed information on ethics, consent, and confidentiality for each survey year is available elsewhere.^25^ This study involved secondary analysis of fully anonymised GPPS data, from which individuals cannot be reidentified by the research team. In line with UK Health Research Authority guidance on anonymised data and secondary use, and in accordance with City St. George's, University of London research ethics committee standard procedures, ethical approval was not required for this study. All analyses complied with the data-sharing agreement with NHS England. This study was pre-registered with the Open Science Framework (osf.io/8g9k3) and is reported in accordance with STROBE guidelines.

### Exposures

Sexual orientation was derived from GPPS question “Which of the following best describes how you think of yourself?”.^25^ To ensure sufficient sample, responses of “Gay/Lesbian,” “Bisexual,” or “Other” were combined into a sexual minority category, with “Heterosexual or Straight” as reference category. Responses of “Prefer not to say” were excluded from the main analysis and reported only descriptively.

Self-reported long-term mental health condition (hereafter ‘mental health condition’) was derived from GPPS question “Which, if any, of the following long-term conditions do you have?”.^25^ Participants could select “A mental health condition” and/or 15 other conditions not reported here. Responses were coded into a binary variable (“mental health condition present” or “not present”). The survey defines a long-term condition as one that lasts, or is expected to last, 12 months or longer. GPPS items do not assess formal diagnosis, severity, treatment, or type of disorder for self-reported mental health conditions.

GPPS questions on protected characteristics such as sexual orientation and disability follow the Government Statistical Service (GSS) harmonised standard and are aligned with terminology recommended by the UK Equality Act 2010.^25^

### Outcomes

We analysed six outcomes. (1) *Mental health needs recognised by the healthcare professional* (“Did you feel that the healthcare professional recognised and/or understood any mental health needs that you might have had?”) and (2) *confidence and trust in the healthcare professional* (“Did you have confidence and trust in the healthcare professional you saw or spoke to?”) had response options: “Yes, definitely”, “Yes, to some extent”, “No, not at all”, and a non-applicable response option (e.g. “Don't know/can't say”). Respondents selecting non-applicable responses were excluded. Binary variables were created by grouping positive (“Yes, definitely” and “Yes, to some extent”) vs. negative responses (“No, not at all”, reference category). (3) *Perceived negative healthcare professional interpersonal skills*, was based on three items assessing interpersonal aspects of the healthcare professional: “Giving you enough time”, “Listening to you”, and “Treating you with care and concern”. Response options were ranged on a five-point Likert scale from “Very good” to “Very poor”. Respondents selecting “Doesn't apply” were excluded. As in previous research,^26^ we created a binary indicator coded as 1 if the respondent rated the healthcare professional as “Poor” or “Very poor” on at least one item, and 0 (reference category) otherwise. (4) *Perceived involvement in care and treatment decisions* (“Were you involved as much as you wanted to be in decisions about your care and treatment?”), and (5) *needs met during the last GP appointment (*“Thinking about the reason for your last general practice appointment, were your needs met?”) also had the response options: “Yes, definitely”, “Yes, to some extent”, “No, not at all”, and a non-applicable response option (e.g., “Don't know/can't say”), which was excluded. Binary variables were created grouping positive (“Yes, definitely” and “Yes, to some extent”) and negative responses (“No, not at all”, reference category). (6) *Help-seeking behaviour prior to GP appointment* was based on the question, “Before you tried to get this appointment, did you do any of the following?”. There were nine answer options covering formal NHS sources (e.g., helplines, online services, pharmacists) and informal sources of support (e.g., advice from friends or family). Those who reported using any prior support were coded as “Yes,” and those who selected “I did not try to get information or advice” were coded as “No” (reference category). All GPPS items referred to participants' most recent GP appointment.

### Statistical analysis

Missingness in all variables of interest ranged from 0.1% to 5.0% after correcting for skip logic, which was largely driven by survey mode, and appeared non-differential between exposure groups. Missingness was small for both, covariates and outcomes, and consistent with a missing at random mechanism once collection mode was considered. Given the low, balanced missingness and high overall completeness, we used complete case analysis as the primary approach, allowing inclusion and missingness to vary between outcomes, in line with guidance that this approach can yield unbiased estimates under these conditions.^27^

We reported baseline characteristics of participants with complete data on exposure variables and covariates as weighted percentages with 95% confidence intervals (CIs). For all models and descriptive statistics, GPPS survey weights were applied using Stata's *pweights* command.

We conducted survey-weighted logistic regression models for each outcome of interest, including sexual orientation and mental health condition as main effects, and their interaction term. The interaction was calculated by entering the cross-product of these two variables into the model, alongside their main effects. Models were adjusted for age (banded), sex, gender identity, ethnicity (five groups), multimorbidity (≥2 long-term health conditions other than mental^28^), area-level deprivation measured by the Index of Multiple Deprivation (IMD) based on patients’ postcode, region (London vs. elsewhere), survey year (2022 or 2023) and survey collection mode. These covariates were selected based on their established associations with healthcare experiences in prior UK studies.^28^^,^^29^

Full detailing of the model including study population flowchart, directed acyclic graphs presenting assumptions underlying the statistical modelling, empirical specification, estimating equation and operationalisation of covariates can be found in the Appendix (pp 5–12).

Results are presented as fully adjusted average predicted probabilities for each exposure group, estimated from the survey-weighted logistic regression models using the *margins* command. Predicted probabilities were obtained for each of the four exposure combinations from the fitted interaction model and point estimates with 95% CIs were obtained using endpoint transformation (inverse-logit transformation of the average log-odds and their corresponding Wald 95% CIs).^30^ Predicted probabilities (0–1) were rescaled to percentages (0–100) for presentation. Joint significance of the interaction between sexual orientation and mental health condition was first tested using Wald chi-square tests. We additionally estimated the relative excess risk due to interaction (RERI) using Stata's *nlcom* command applied to inverse-logit-transformed coefficients, capturing excess or reduced risk beyond additivity. The adjusted predicted probabilities *p*11, *p*10, *p*01, and *p*00 correspond to each combination of sexual orientation (sexual minority vs. heterosexual) and mental health condition (present vs. absent), with p00 referring to heterosexuals without a mental health condition. RERI was calculated as RERI = *p*11 – *p*10 – *p*01 + *p*00, reflecting departure from additivity on the probability scale.^31^

For non-significant interactions, adjusted predicted probabilities were recalculated from models without the interaction term. Pairwise group comparisons for each outcome were obtained using *margins, pwcompare(effects)* from the fully adjusted models.

Lastly, we conducted exploratory analyses to examine whether the quality–related outcomes explained variation in prior help-seeking. Results are presented in the appendix (pp 30–31). Results for overall main effects, pairwise comparisons of fully adjusted predicted probabilities *per* outcome, stepwise and fully adjusted models, and exploratory three-way interactions (age, ethnicity and deprivation level) are presented in the Appendix (pp 16–29, 32–73). Analyses were conducted using Stata 15.1.^32^

### Role of funding source

This article was not commissioned or funded by any external agency.

## Results

Complete data on sexual orientation, mental health condition, and all covariates were available for 1,277,383 respondents. Based on weighted data, 1,169,129 (89.3%) identified as heterosexual, 47,626 (5.6%) as sexual minority, and 60,628 (5.1%) preferred not to say.

Sexual minority individuals were younger, more likely to report mental health conditions, transgender/non-binary identities, and at least one long-term health condition than both heterosexuals and individuals who preferred not to disclose their sexual orientation. Both sexual minority individuals and those who preferred not to state their sexual orientation were more likely to reside in London and in more deprived areas, when compared to heterosexual respondents. All groups had majority White representation, but sexual minorities were overrepresented among mixed ethnicities relative to other groups and Asian/Asian British groups were overrepresented among individuals not stating their sexual orientation.

Descriptive statistics by sexual orientation are shown in Table 1. For descriptive statistics by mental health status see Appendix (Table S1, Appendix pp 13 and 14).

**Table 1:** Survey respondents demographics by sexual orientation group.

	Heterosexual	Sexual minority	Prefer not to say	Total
	N (weighted %)	N (weighted %)	N (weighted %)	N (weighted %)
N	1,169,129 (100%)	47,626 (100%)	60,628 (100%)	1,277,383 (100%)
*Self-reported mental health condition*				
No	1,059,390 (88.6%)	35,574 (69.2%)	54,386 (86.8%)	1,149,350 (87.4%)
Yes	109,739 (11.4%)	12,052 (30.8%)	6242 (13.2%)	128,033 (12.6%)
*Age*				
16–24	36,402 (8.2%)	5796 (22.1%)	3459 (12.8%)	45,657 (9.2%)
25–34	84,961 (15.9%)	9185 (29.9%)	6532 (20.0%)	100,678 (16.9%)
35–44	133,925 (17.5%)	8965 (20.1%)	10,962 (22.7%)	153,852 (17.9%)
45–54	184,248 (17.7%)	8407 (13.4%)	11,698 (18.4%)	204,353 (17.5%)
55–64	259,811 (17.2%)	7915 (8.6%)	11,599 (12.7%)	279,325 (16.5%)
65–74	260,359 (12.8%)	4664 (3.6%)	9395 (7.3%)	274,418 (12.0%)
75+	209,423 (10.8%)	2694 (2.2%)	6983 (6.0%)	219,100 (10.1%)
*Sex*				
Female	666,815 (52.0%)	23,631 (44.5%)	32,021 (47.0%)	722,467 (51.3%)
Male	497,452 (47.5%)	21,534 (49.1%)	21,108 (39.5%)	540,094 (47.2%)
Non-binary	721 (0.1%)	1387 (4.1%)	460 (0.8%)	2568 (0.3%)
Prefer to self-describe	732 (0.1%)	381 (0.9%)	718 (1.3%)	1831 (0.2%)
Prefer not to say	3409 (0.3%)	693 (1.4%)	6321 (11.3%)	10,423 (1.0%)
*Gender identity*				
Cisgender	1,164,184 (99.5%)	44,230 (91.3%)	51,204 (83.8%)	1,259,618 (98.2%)
Transgender	2714 (0.3%)	2337 (6.4%)	1540 (2.6%)	6591 (0.7%)
Prefer not to say	2231 (0.2%)	1059 (2.4%)	7884 (13.6%)	11,174 (1.0%)
*Ethnicity*				
White	998,740 (83.2%)	36,580 (79.8%)	32,453 (55.3%)	1,067,773 (81.6%)
Mixed/multiple ethnic groups	15,779 (1.8%)	1702 (4.0%)	1448 (3.0%)	18,929 (2.0%)
Asian/Asian British	92,936 (9.1%)	4948 (8.6%)	18,019 (26.9%)	115,903 (10.0%)
Black/African/Caribbean/Black British	43,592 (4.0%)	2398 (3.9%)	4350 (6.9%)	50,340 (4.2%)
Other ethnic group	18,082 (1.8%)	1998 (3.7%)	4358 (7.8%)	24,438 (2.2%)
*Multimorbidity*				
No LTCs	404,993 (43.3%)	16,614 (38.4%)	24,889 (48.1%)	446,496 (43.3%)
No multimorbidity	365,419 (30.1%)	15,885 (33.9%)	17,805 (28.0%)	399,109 (30.2%)
Multimorbidity	398,717 (26.6%)	15,127 (27.7%)	17,934 (23.9%)	431,778 (26.5%)
*Patient IMD quintile*				
Q1 (most deprived)	221,182 (19.5%)	12,558 (25.6%)	18,716 (30.8%)	252,456 (20.4%)
Q2	227,993 (20.1%)	12,333 (26.2%)	15,462 (25.4%)	255,788 (20.7%)
Q3	242,018 (20.2%)	9510 (19.8%)	11,350 (18.5%)	262,878 (20.1%)
Q4	243,958 (20.2%)	7548 (16.1%)	8489 (14.1%)	259,995 (19.6%)
Q5 (least deprived)	233,978 (20.0%)	5677 (12.4%)	6611 (11.2%)	246,266 (19.1%)
*Region*				
Outside London	982,692 (84.1%)	34,392 (75.9%)	42,588 (72.8%)	1,059,672 (83.1%)
London	186,437 (15.9%)	13,234 (24.1%)	18,040 (27.2%)	217,711 (16.9%)

### Mental health needs recognised by the healthcare professional

Heterosexuals had the highest probability of having their mental health needs recognised regardless of mental health condition (MHC) status (without: 82.5%; with: 81.9%), while sexual minorities without MHC had the lowest (80.1%; Table 2). Sexual minorities with MHC reported intermediate probabilities (81.7%), not significantly differing from other groups. A significant interaction between sexual minority status and MHC accounted for an increase in the probability of a positive endorsement by 2.13 percentage points for sexual minorities with MHC beyond additive effects (Table 3).

**Table 2:** Fully adjusted predicted probabilities for outcomes *per* sexual orientation and mental health condition group.

Outcome	Heterosexual no MHC (HnoMHC)	Sexual minority no MHC (SMnoMHC)	Heterosexual MHC (HMHC)	Sexual minority MHC (SMMHC)	Group differences significant at p-value^a^ ≤ 0.002
	Predicted probabilities [95% CIs]				
Mental health needs recognised by the healthcare professional (1)	82.5 [82.3–82.7]	80.1 [79.2–81.0]	81.9 [81.5–82.3]	81.7 [80.6–82.7]	HMHC, HnoMHC > SMnoMHC
Confidence and trust in healthcare professional at last appointment (2)	94.3 [94.3–94.4]	93.9 [93.5–94.3]	93.0 [92.8–93.2]	93.3 [92.8–93.9]	HnoMHC > HMHC, SMMHC SMnoMHC > HMHC
Negative perception of healthcare professional interpersonal skills (3)	6.5 [6.4–6.6]	7.1 [6.8–7.4]	8.2 [7.9–8.4]	8.9 [8.4–9.3]	SMMHC > HMHC > SMnoMHC > HnoMHC
Involvement in care and treatment decisions (4)	91.4 [91.3–91.5]	90.8 [90.4–91.2]	90.4 [90.1–90.6]	89.8 [89.3–90.2]	HnoMHC > HMHC, SMnoMHC > SMMHC
Needs met during the last GP appointment (5)	92.4 [92.3–92.5]	91.9 [91.5–92.3]	91.0 [90.8–91.3]	91.3 [90.7–91.9]	HnoMHC > HMHC, SMMHC SMnoMHC > HMHC
Help-seeking prior to GP appointment (6)	62.5 [62.3–62.6]	66.0 [65.3–66.7]	64.3 [63.8–64.7]	67.7 [66.9–68.4]	SMMHC > SMnoMHC > HMHC > HnoMHC

*Note*. MHC, Mental health condition.

Sample size *per* outcome: (1) 500,618; (2) 1,125,400; (3) 1,088,059; (4) 1,019,397; (5) 1,129,005; (6) 1,138,852.

a Bonferroni corrected (0.05/24 (number of tests) = 0.002).

**Table 3:** Interaction effects (χ^2^ and RERI) between sexual minority orientation and presence of mental health condition.

Outcome	χ^2^ (df)	p-value	RERI [95% CIs]	p-value
Mental health needs recognised by the healthcare professional (1)	8.75 (1)	0.003	2.13 [0.71–3.55]	0.003
Confidence and trust in healthcare professional at last appointment (2)	5.46 (1)	0.019	0.75 [0.10–1.40]	0.024
Negative perception of healthcare professional interpersonal skills (3)	0.06 (1)	0.81	0.22 [−0.54 to 0.98]	0.57
Involvement in care and treatment decisions (4)	3.76 (1)	0.052	0.81 [−0.06 to 1.68]	0.07
Needs met during the last GP appointment (5)	5.05 (1)	0.02	0.85 [0.10–1.60]	0.03
Help-seeking prior to GP appointment (6)	1.77 (1)	0.18	−1.16 [−2.74 to 0.43]	0.15

Sample size *per* outcome: (1) 500,618; (2) 1,125,400; (3) 1,088,059; (4) 1,019,397; (5) 1,129,005; (6) 1,138,852.

### Confidence and trust in healthcare professional at last appointment

Confidence and trust in the healthcare professional showed no significant differences by sexual orientation within mental health condition (MHC) strata: without MHC, heterosexuals (94.3%) and sexual minorities (93.9%) did not differ significantly, nor did heterosexuals (93.0%) and sexual minorities (93.3%) with MHC (Table 2). However, both groups with a mental health condition (MHC) had significantly lower probabilities than heterosexuals without MHC. Sexual minorities did not differ from each other irrespective of mental health condition (MHC) status (Table 2). A significant interaction accounted for an increase in the probability of a positive endorsement by 0.75 percentage points for sexual minorities with MHC beyond additive effects (Table 3).

### Negative perception of healthcare professional's interpersonal skills

Heterosexuals without a mental health condition had the lowest predicted probability of negative perceptions (6.5%), followed by sexual minorities without (7.1%), heterosexuals with (8.2%), and sexual minorities with a mental health condition (8.9%; all groups differed; Table 2). There was no evidence of an interacting effect between sexual orientation and mental health condition (Table 3).

### Involvement in care and treatment decisions

Heterosexuals without a mental health condition had the highest predicted probability of feeling involved in care decisions (91.4%), exceeding all other groups. Sexual minorities without a mental health condition (90.8%) and heterosexuals with a mental health condition showed similar intermediate predicted probabilities (90.4%) and did not differ from each other. Sexual minorities with a mental health condition had the lowest predicted probability of all groups (89.8%; Table 2). There was no evidence of an interacting effect between sexual orientation and mental health condition (Table 3).

### Needs met during the last GP appointment

Needs met during the last GP appointment showed no significant differences by sexual orientation within mental health condition (MHC) strata: without MHC, heterosexuals (92.4%) and sexual minorities (91.9%) did not differ significantly, nor did heterosexuals (91.0%) and sexual minorities (91.3%) with MHC (Table 2). However, both groups with a mental health condition (MHC) had significantly lower probabilities than heterosexuals without MHC, with heterosexuals with MHC also below sexual minorities without MHC. Sexual minorities did not differ from each other irrespective of MHC status (Table 2). A significant interaction accounted for a 0.85 percentage-point increase for sexual minorities with MHC beyond additive effects (Table 3).

### Help-seeking prior to GP appointment

**Fig. 1:**
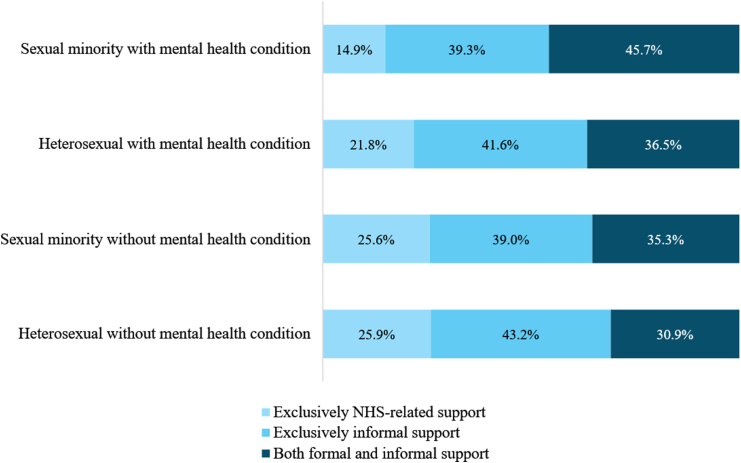
Distribution of sources of support sought before GP appointment by sexual orientation and presence of mental health condition.

Sexual minorities with a mental health condition showed the highest predicted probability of prior help-seeking (67.7%), followed by sexual minorities without a mental health condition (66.0%), heterosexuals with a mental health condition (64.3%) and lastly heterosexuals without a mental health condition, with the lowest predicted probabilities (62.5%; Table 2). Fig. 1 shows the types of support considered prior to a GP appointment by sexual orientation and mental health status. Exploratory analyses (Table S10, Appendix pp 30 and 31) showed that the odds for seeking help before the GP appointment were higher for those who felt that mental health needs were not recognised (OR 1.4 [95% CI: 1.4–1.5]), with overall unmet needs (OR 1.3 [95% CI: 1.2–1.4]), with lower ratings of the professional's interpersonal skills (OR 1.3 [95% CI: 1.2–1.4]), and lack of involvement in decision-making (OR 1.1 [95% CI: 1.1–1.2], all p < 0.001). There was no evidence of an interacting effect of sexual orientation and mental health condition on the probability of seeking help before the GP appointment (Table 3).

## Discussion

We found that individuals with sexual minority status, long-term mental health condition and their combination were less likely to report positive primary care experiences than heterosexual individuals without a mental health condition. Particularly for the quality-related outcomes of negative perceptions of healthcare professional's interpersonal skills and involvement in care decisions, sexual minority individuals with a mental health condition had the least favourable experiences of all groups, a pattern consistent with cumulative disadvantage at the intersection of these two characteristics.

By contrast, for mental health needs recognised, confidence and trust in the healthcare professional, and overall needs met, there was no evidence of compounded disadvantage. Instead, significant interaction effects suggested that this intersection group had slightly higher probabilities of positive endorsement than would be expected under an additive model, indicating that the intersection of these characteristics might confer a potential buffering of the disadvantages associated with each characteristic alone.

The absence of compounded disadvantage and the indication of potentially protective effects for these three outcomes should, however, be interpreted cautiously. Although statistically significant, the observed differences in predicted probabilities and interaction effects are modest. Nonetheless, even modest differences might carry non-negligible public health significance when scaled to the population level.^33^ For example, the significant interaction effect on confidence and trust in healthcare professionals yielded a 0.75 percentage point increase in the predicted probability of positive endorsement among sexual minorities with mental health conditions. Although our smallest protective effect, this translates, given 5.6% sexual minority prevalence in the data, 30.8% mental health condition prevalence within that group (Table 1), and England's approximately 61.8 million population,^34^ to roughly 8000 additional sexual minorities with mental health conditions expressing confidence in their healthcare provider compared to additive expectations.

While the GPPS lacks the information necessary to examine the mechanisms that may underlie these potential buffers, emerging evidence on the intersection of sexual orientation and ethnicity offers some insight. This research suggests that, in some circumstances, intersecting minority identities can mobilise psychosocial resources, coping mechanisms, and resilience processes associated with one minoritised identity to buffer the adverse effects of stigma linked to another.^35^ Further research is needed to identify which individual-level factors within sexual minority orientation and mental health status are central to these buffering effects, and how characteristics such as internalised sexual stigma or pride, type and severity of mental health condition, and related psychosocial processes shape their magnitude and direction.

Regardless of sexual orientation, confidence and trust in the healthcare professional and overall needs met were the lowest among participants with a long-term mental health condition, followed by sexual minority and heterosexual individuals without one. This pattern aligns with previous research showcasing that mental health issues are associated with lower care satisfaction,^36^ while reduced trust and confidence may be partly explained by disorder-related perceptual biases that might negatively shape how interactions with healthcare professionals are interpreted.^36^ In addition, primary care is often perceived as overly focused on physical health, with mental health concerns minimised or rushed,^37^ which might further lower the confidence individuals with mental health issues have in primary-care professionals’ ability to help.

We also found that sexual minorities without a long-term mental health condition had the lowest probabilities of having their mental health needs recognised, albeit not significantly different from sexual minorities with a long-term mental health condition. Given consistent evidence that sexual minority populations are at elevated risk of mental health difficulties,^6^^,^^19^ one possible explanation is that some individuals in this group are experiencing emerging or undiagnosed problems, whose needs are not yet recognised in primary care.

Sexual minority individuals, irrespective of mental health status, and heterosexual individuals with mental health conditions had higher probabilities of seeking help prior to scheduling a GP appointment than heterosexuals without a mental health condition. This pattern was partly explained by greater dissatisfaction with primary care experiences, including unrecognised mental health needs, overall unmet needs, low involvement in care decisions, and negative ratings of healthcare professional's interpersonal skills. This is consistent with previous evidence that low satisfaction or poor prior healthcare experiences can undermine confidence in receiving adequate future care and discourage formal help-seeking.^11^^,^^21^, ^22^, ^23^, ^24^^,^^37^^,^^38^

While prior help-seeking was not confined to informal sources and typically involved a combination of informal and formal options, many of the formal alternatives (such as NHS websites, online platforms, and 111 phone services) may entail lower social risk than an in-person GP consultation, given the more limited interpersonal contact, while still drawing on expert sources of information and advice. This interpretation is supported by previous research showing associations between digital and independent forms of health engagement and previous negative in-person healthcare experiences.^39^ Having a mental health condition is often associated with greater mental health literacy and awareness, which in turn has been shown to impact help-seeking behaviours positively.^40^ Sexual minorities have also been shown to display comparatively higher mental health literacy. Thus, the higher rates of prior help-seeking in these groups could also reflect greater awareness and a more proactive approach to seeking support.

A major strength of our study is the use of a large, recent, nationally representative GPPS sample and survey weights to adjust for sampling design and non-response. Pooling two years increased sample size, enabling us to generate estimates for smaller subgroups, such as sexual minority individuals with a mental health condition, who represent a small proportion of the overall population, while also allowing adjustment for multiple covariates, minimising confounding. However, a key related limitation of this grouping strategy is that it combines heterogeneous sexual minority identities into a single category, which may obscure potentially relevant differences between subgroups. Although unlikely as only approximately 1% of individuals registered with a GP respond to the GPPS, pooling may introduce repeated responses across survey years, thus the potential for bias due to this factor cannot be dismissed.

Current findings might be subject to reporting biases, as some individuals may be reluctant to report negative experiences with NHS services. A nationally representative survey commissioned by Healthwatch England found that about a quarter of adults in England reported a poor NHS care experience in the past year, yet approximately 56% took no action in regard to their concerns.^41^ Some of the reasoning behind taking no action included the belief that the NHS would not use their complaint to improve services, expectations that organisations would not respond effectively, or the perception that the NHS would not take their concerns seriously.^41^ Given the reluctance to report negative experiences, GPPS respondents may be more satisfied or more trusting that their feedback will lead to improvement. This selection could bias estimates towards a more positive view of NHS primary care, under-representing those with poorer experiences or greater distrust.

Furthermore, the GPPS assesses primary care experiences using predefined response categories, which do not capture context or individual experiences. Long-term mental health conditions were self-reported through a single item and did not distinguish between type, severity, duration, or treatment status, limiting investigation of potential mental health diagnostic differences. Lastly, the cross-sectional nature of the data also limits any conclusions on directionally of the effects observed and, while desirable for ease of interpretation, the dichotomization of outcomes might minimize and obscure variances.

Healthcare systems do not operate in isolation but are embedded within broader social contexts that shape how services are both delivered and experienced. Although legal protections such as the Equality Act 2010 aim to prevent discrimination based on sexual orientation, they do not immediately dispel sexual minorities' fear of discrimination or the biases held by some healthcare professionals. Whether the suboptimal experiences reported by sexual minorities arise from perceptual biases shaped by past negative encounters, structural shortcomings in care, or both, evidence indicates that health disparities within these groups are widening rather than narrowing.^19^ The limited enforcement of the Equality Act 2010, reflected in persistent disparities,^19^ the scarcity of healthcare policies explicitly addressing sexual minorities' needs,^42^ and lack of cultural training from both practitioners^38^ and service user's perspectives,^43^ highlights the need to better address this population's unique and intersectional healthcare requirements.

Our findings show that intersectionality is not solely a framework for identifying compounded disadvantage but also a lens for understanding protective effects.^44^ Further research is needed to investigate whether potential buffering effects arise from resilience processes, increased healthcare engagement among individuals with multiple minority characteristic or differential treatment by healthcare professionals following disclosure of mental health needs. Exploring such mechanisms is central to improving primary healthcare practice for individuals at the intersection of minoritised identities as it might inform pathways to reduce healthcare inequalities.

## Contributors

AGM, AKF, DD and SM were responsible for the conceptualisation of this work. AGM was responsible for data curation, methodology, software, formal analysis, visualisation and writing of the original draft. AKF was responsible for supervision and validation, with inputs from SM, EvdV and DD. All authors contributed to the final writing of this work, reviewing and editing the final text. All authors had access to and verified the data used in this work. All authors have read and approved the final version of this work.

## Data sharing statement

Individual-level data from GPPS was obtained from Ipsos MORI via an NHS England sharing agreement with our team at City St George's, University of London (formerly City, University of London; Reference ID3063). Further information about the GPPS and dataset is available at https://gp-patient.co.uk or by contacting gppatientsurvey@ipsos.com. The authors of this study cannot share individual-level data as they are the property of NHS England and managed by Ipsos MORI. Researchers can obtain cross-sectional data from 2007 through 2024 for all available patients by submitting a request to NHS England. Enquiries regarding the code developed for the analysis of this study can be made by contacting the corresponding author.

## Declaration of interests

AGM was supported by a Centrally Funded MPhil/PhD Doctoral Studentship by City St. George's, University of London; SM acknowledges salary support from the UKRI (Grant MR/V049879/1). DD is partially supported by NIHR Maudsley Biomedical Research Centre, South London and Maudsley NHS Trust. The views expressed are those of the author(s) and not necessarily those of the NIHR or the Department of Health and Social Care. EvdV is supported by a Veni grant (ZonMw, 09150161910016). The authors have no conflicts of interest to declare.
