# Supplementary material for: Primary care experiences at the intersection of sexual minority status and long-term mental health conditions in England: a cross-sectional analysis using the English General Practice Patient Survey
Source: eClinicalMedicine. 2026 Jun 11;96:104016. doi: 10.1016/j.eclinm.2026.104016 (PMC13272551; doi:10.1016/j.eclinm.2026.104016)
Supplement: Supplementary Material [file mmc1.docx]

*Primary care experiences at the intersection of sexual minority status and long-term mental health conditions in England: a cross-sectional analysis using the English General Practice Patient Survey.*

**Supplementary Appendix**

Table of contents

| **Figure / Table** | **Title** | **Page** |
| --- | --- | --- |
| Figure S1 | Study population flow chart | 5 |
| Figure S2 | Directed acyclic graph (DAG) model showing assumptions underlying the statistical model for sexual orientation and self-reported long-term mental health condition on primary care experience outcomes | 6 |
| Figure S3 | Directed acyclic graph (DAG) model showing assumptions underlying the statistical model for sexual orientation and self-reported long-term mental health condition on help-seeking prior to attempting to schedule a GP appointment | 7 |
| Box S1 | Details of the empirical approach | 8–10 |
| Box S2 | Operationalisation of covariates | 11–12 |
| Table S1 | Survey respondents’ demographic characteristics by presence/absence of a self-reported long-term mental health condition (unweighted counts, weighted percentages and 95% confidence intervals) | 13–14 |
| Table S2 | Fully adjusted predicted probabilities for outcomes separate for sexual orientation and mental health condition group (overall main effects) | 15 |
| Table S3 | Pairwise comparisons of fully adjusted predicted probabilities per outcome | 16–17 |
| Table S4 | Stepwise adjusted logistic regression models for mental health needs recognised by the healthcare professional | 18–19 |
| Table S5 | Stepwise adjusted logistic regression models for confidence and trust in the healthcare professional | 20–21 |
| Table S6 | Stepwise adjusted logistic regression models for negative perception of healthcare professional interpersonal skills | 22–23 |
| Table S7 | Stepwise adjusted logistic regression models for involvement in care and treatment decisions | 24–25 |
| Table S8 | Stepwise adjusted logistic regression models for needs met during the last GP appointment | 26–27 |
| Table S9 | Stepwise adjusted logistic regression models for help-seeking prior to attempting a GP appointment | 28–29 |
| Table S10 | Fully adjusted logistic regression model assessing the association between primary care experience quality indicators and prior help-seeking behaviour | 30–31 |
| Table S11 | Fully adjusted logistic regression model assessing the interaction between sexual orientation and self-reported long-term mental health condition on mental health needs recognised by the healthcare professional | 32 |
| Table S12 | Fully adjusted logistic regression model assessing the interaction between sexual orientation and self-reported long-term mental health condition on confidence and trust in the healthcare professional | 33 |
| Table S13 | Fully adjusted logistic regression model assessing the interaction between sexual orientation and self-reported long-term mental health condition on perceived negative healthcare professional interpersonal skills | 34 |
| Table S14 | Fully adjusted logistic regression model assessing the interaction between sexual orientation and self-reported long-term mental health condition on involvement in care and treatment decisions | 35 |
| Table S15 | Fully adjusted logistic regression model assessing the interaction between sexual orientation and self-reported long-term mental health condition on needs met during the last GP appointment | 36 |
| Table S16 | Fully adjusted logistic regression model assessing the interaction between sexual orientation and self-reported long-term mental health condition on help-seeking prior to attempting a GP appointment | 37 |
| Table S17 | Fully adjusted logistic regression model assessing the interaction between sexual orientation, self-reported long-term mental health condition and age on help-seeking prior to attempting a GP appointment | 38–39 |
| Table S18 | Fully adjusted logistic regression model assessing the interaction between sexual orientation, self-reported long-term mental health condition and age on mental health needs recognised by the healthcare professional | 40–41 |
| Table S19 | Fully adjusted logistic regression model assessing the interaction between sexual orientation, self-reported long-term mental health condition and age on confidence and trust in the healthcare professional | 42–43 |
| Table S20 | Fully adjusted logistic regression model assessing the interaction between sexual orientation, self-reported long-term mental health condition and age on perceived negative healthcare professional interpersonal skills | 44–45 |
| Table S21 | Fully adjusted logistic regression model assessing the interaction between sexual orientation, self-reported long-term mental health condition and age on involvement in care and treatment decisions | 46–47 |
| Table S22 | Fully adjusted logistic regression model assessing the interaction between sexual orientation, self-reported long-term mental health condition and age on needs met during the last GP appointment | 48–49 |
| Table S23 | Fully adjusted logistic regression model assessing the interaction between sexual orientation, self-reported long-term mental health condition and ethnicity on help-seeking prior to attempting a GP appointment | 50–51 |
| Table S24 | Fully adjusted logistic regression model assessing the interaction between sexual orientation, self-reported long-term mental health condition and ethnicity on mental health needs recognised by the healthcare professional | 52–53 |
| Table S25 | Fully adjusted logistic regression model assessing the interaction between sexual orientation, self-reported long-term mental health condition and ethnicity on confidence and trust in the healthcare professional | 54–55 |
| Table S26 | Fully adjusted logistic regression model assessing the interaction between sexual orientation, self-reported long-term mental health condition and ethnicity on perceived negative healthcare professional interpersonal skills | 56–57 |
| Table S27 | Fully adjusted logistic regression model assessing the interaction between sexual orientation, self-reported long-term mental health condition and ethnicity on involvement in care and treatment decisions | 58–59 |
| Table S28 | Fully adjusted logistic regression model assessing the interaction between sexual orientation, self-reported long-term mental health condition and ethnicity on needs met during the last GP appointment | 60–61 |
| Table S29 | Fully adjusted logistic regression model assessing the interaction between sexual orientation, self-reported long-term mental health condition and deprivation level on help-seeking prior to attempting a GP appointment | 62–63 |
| Table S30 | Fully adjusted logistic regression model assessing the interaction between sexual orientation, self-reported long-term mental health condition and deprivation level on mental health needs recognised by the healthcare professional | 64–65 |
| Table S31 | Fully adjusted logistic regression model assessing the interaction between sexual orientation, self-reported long-term mental health condition and deprivation level on confidence and trust in the healthcare professional | 66–67 |
| Table S32 | Fully adjusted logistic regression model assessing the interaction between sexual orientation, self-reported long-term mental health condition and deprivation level on perceived negative healthcare professional interpersonal skills | 68–69 |
| Table S33 | Fully adjusted logistic regression model assessing the interaction between sexual orientation, self-reported long-term mental health condition and deprivation level on involvement in care and treatment decisions | 70–71 |
| Table S34 | Fully adjusted logistic regression model assessing the interaction between sexual orientation, self-reported long-term mental health condition and deprivation level on needs met during the last GP appointment | 72–73 |
| STROBE | STROBE Statement—checklist of items that should be included in reports of observational studies | 74–75 |

Figure S1 – Study population flowchart


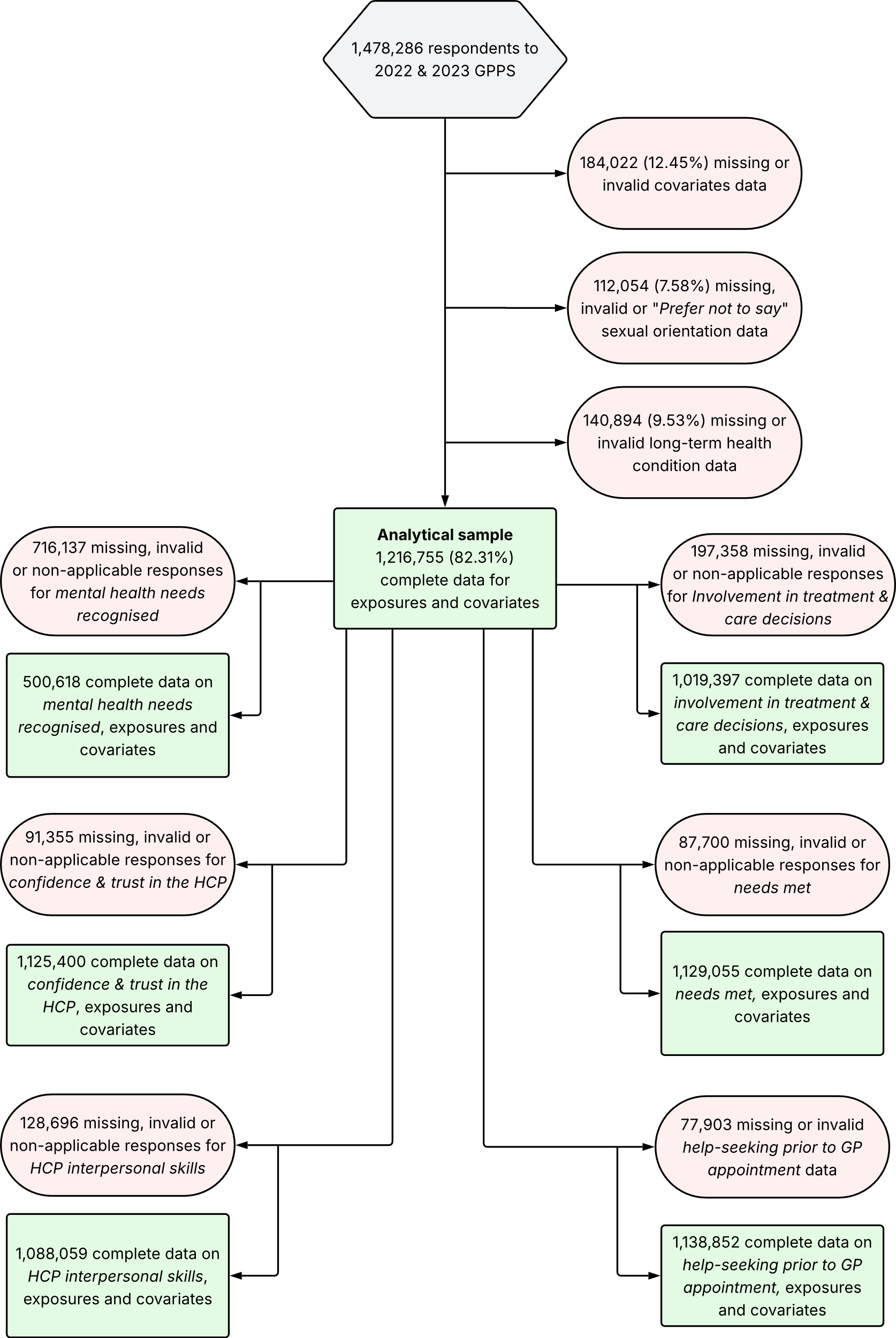


*GPPS*: General Practice Patient Survey

*HCP*: Healthcare professional

Figure S2 – Directed acyclic graph (DAG) Model showing assumptions underlying statistical model for sexual minority orientation and self-reported long-term mental health condition on primary care experience outcomes.


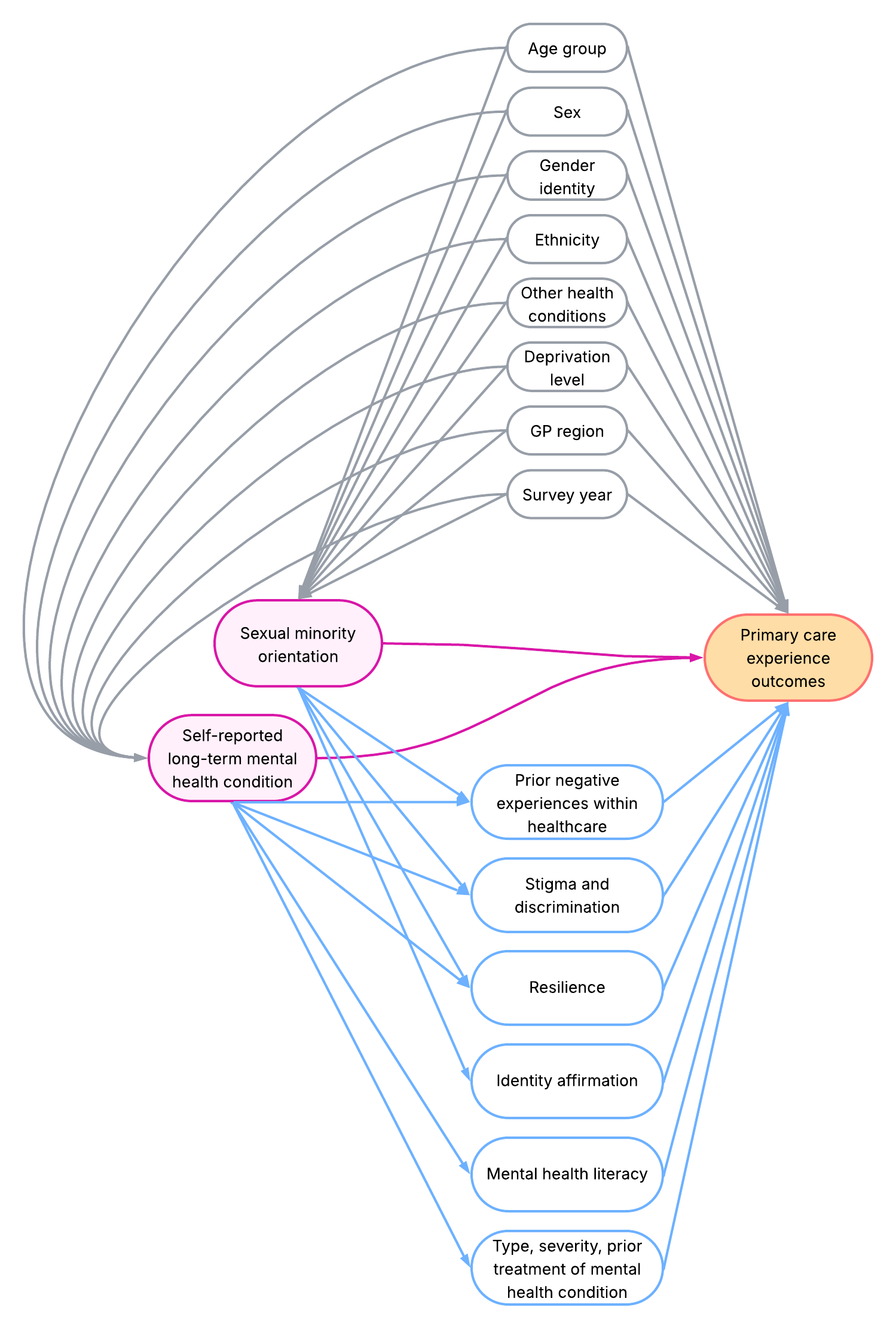


*Confounders measured in GPPS: adjusted for in all analyses*

*Potential mediators not measured in GPPS: could not be explored in the present analysis*

Figure S3 – Directed acyclic graph (DAG) Model showing assumptions underlying statistical model for sexual minority orientation and self-reported long-term mental health condition on help-seeking prior to trying to schedule a GP appointment.


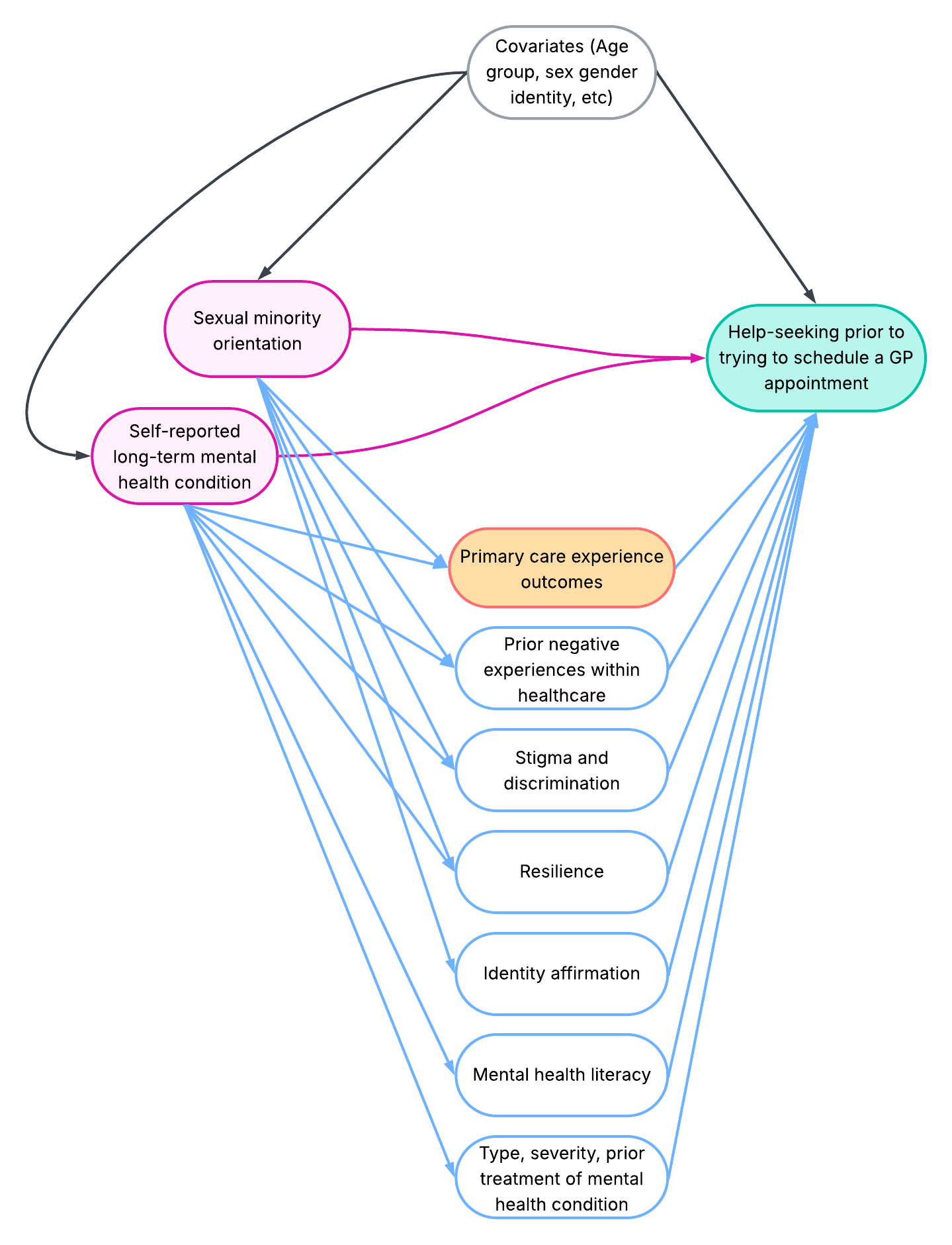


*Confounders measured in GPPS: adjusted for in all analyses.*

*Potential mediators not measured in GPPS: could not be explored in the present analysis.*

*Primary care quality-related outcomes from previous model were included in this model as potential explanatory variables.*

Box S1 – Details of the empirical approach

*Variables included in the model*

The study included two exposure variables. First, sexual orientation was derived from the GPPS question, “*Which of the following best describes how you think of yourself?*” The original response options were “Heterosexual or straight”, “Gay/Lesbian”, “Bisexual”, “Other”, and “Prefer not to say”. For analysis, these were recoded into a binary variable distinguishing sexual minority (Gay/Lesbian, Bisexual, Other) from heterosexual (reference category). Responses of “Prefer not to say” were excluded from the main analyses.

Second, the presence of a self-reported long-term mental health condition was derived from the GPPS question, “*Which, if any, of the following long-term conditions do you have?*” Participants could select from 17 long-term condition options (e.g. arthritis, diabetes, autism, neurological conditions, hearing or vision impairments, and others). This list included the option “A mental health condition”, which was used to construct a binary variable: respondents selecting this option were coded as “mental health condition”, and all others were coded as “no mental health condition” (reference category).

We included as covariates age group, sex, gender identity, ethnicity, multimorbidity, area-level deprivation measured by the Index of Multiple Deprivation (IMD) based on patients’ postcode, GP region, survey year and survey collection mode.

*Empirical specification*

We modelled the probability of outcome *O_r,y_* (mental health needs recognised by the healthcare professional (HCP; yes/no), confidence and trust in the HCP (yes/no), negative perception of HCP interpersonal skills (any “very poor” or “poor” vs. no “very poor” or “poor”), involvement in treatment and care decisions (yes/no), needs met during last GP appointment (yes/no), help-seeking prior to attempting a GP appointment (yes/no)) for respondent *r* in GPPS year *y* using a logit model of the following form:

Logit *P*(*O_r,y_* = 1) = *β*_0_ + ***β*_1_SexualOrientation**_r,y_ + ***β*_2_MentalHealthCondition**_r,y_ + ***β*_3_**(**SexualOrientation** x **MentalHealthCondition**)_r,y_ + ***β*_4_AgeGroup**_r,y_ + ***β*_5_Sex**_r,y_ + ***β*_6_GenderIdentity**_r,y_ + ***β*_7_Ethnicity**_r,y_ + ***β*_8_Multimorbidity**_r,y_ + ***β*_9_IMD**_r,y_ + ***β*_10_GPRegion**_r,y_+ ***β*_11_SurveyYear**_r,y_ + ***β*_12_CollectionMode**_r,y_ + ε_r,y_

- **SexualOrientation**_r,y_ is a 1-column binary variable derived from the question *“Which of the following best describes how you think of yourself?”*. Respondents identifying as “Gay/Lesbian”, “Bisexual”, or “Other” were coded as sexual minority (1), and those identifying as “Heterosexual or straight” were coded as heterosexual (0, reference group). “Prefer not to say” responses were excluded from the analytic sample.
- **MentalHealthCondition**_r,y_ is a 1-column binary variable derived from the question *“Which, if any, of the following long-term conditions do you have?”*. Individuals selecting *“A mental health condition”* were coded as mental health condition (1), and all others were coded as no mental health condition (0, reference category).
- **AgeGroup**_r,y_ is a 1-column ordinal variable with seven levels representing the respondent’s age group (16–24, 25–34, 35–44, 45–54, 55–64, 65–74, 75+), with the category 16–24 used as the reference group.
- **Sex**_r,y_ is a 1-column nominal variable with five levels corresponding to responses to the question *“Which of the following best describes you?”*, with levels “Female”, “Male”, “Non-binary”, “Prefer to self-describe”, and “Prefer not to say”. “Female” is used as the omitted reference category.
- **GenderIdentity**_r,y_ is a 1-column nominal variable with three levels derived from the question *“Is your gender identity the same as the sex you were assigned at birth?”*. The responses “Yes”, “No”, and “Prefer not to say” were recoded as cisgender (0, reference group), transgender (1), and prefer not to say (2).
- **Ethnicity**_r,y_ is a 1-column nominal variable representing the respondent’s ethnic group (“White”, “Mixed or Multiple ethnic groups”, “Asian or Asian British”, “Black, Black British, Caribbean or African”, “Other ethnic group”). White is the omitted reference category.
- **Multimorbidity**_r,y_ is a 1-column nominal variable derived from the long-term conditions question, distinguishing (a) no long-term conditions (reference), (b) one long-term condition (“no multimorbidity”), and (c) two or more long-term conditions (“multimorbidity”).
- **IMD**_r,y_ is a 1-column ordinal variable capturing neighbourhood deprivation level based on the Index of Multiple Deprivation quintiles (Q1–Q5). Q1 (most deprived) is the omitted reference category.
- **GPRegion**_r,y_ is a 1-column binary variable indicating whether the respondent’s general practice is located in London (1) or outside London (0, reference group).
- **SurveyYear**_r,y_ is a 1-column binary indicator distinguishing 2023 (1) from 2022 (0, reference group).
- **CollectionMode** _r,y_ is a 1-column binary indicator distinguishing online (1) from paper (0, reference group).
- **β₁ – β₁_2_** are vectors of coefficients corresponding to the variables listed above.
- **ε**_r,y_ is the error term

*Estimation procedure*

We estimated the full model outlined in the equation above. All the analyses were weighted by the survey weight provided with the GPPS datasets. Weights were accounted for in estimation and inference using the command *pweights* in Stata.

*Reporting of results and inferences*

Based on the fitted logistic regression models, we estimated adjusted average predicted probabilities to compare groups defined by our key exposures: sexual orientation and mental health status (presence or absence of a self-reported long-term mental health condition). Average predicted probabilities and corresponding 95% confidence intervals were derived using endpoint transformation. Specifically, predicted log-odds were first calculated at the observed values of all covariates and averaged across the sample. Wald 95% confidence intervals were then obtained on the log-odds scale for each joint exposure group (e.g. sexual minority individuals with a long-term mental health condition, heterosexual individuals with a long-term mental health condition), and both point estimates and confidence limits were transformed to the probability scale using the inverse logit function. This approach assumes approximate normality of the log-odds and ensures that predicted probabilities and confidence intervals are bounded between 0 and 1. For ease of interpretation, all predicted probabilities are presented as percentages (0–100). For completeness, stepwise adjusted logistic regression estimates are additionally reported as odds ratios with 95% confidence intervals in Tables S2 and S7.

*Testing interactions and contrast between exposure levels*

For each outcome, we conducted a χ² test of the joint null hypothesis that all interaction terms were equal to zero (i.e. a global test for interaction). Results of these tests are reported in the Results section. For outcomes where evidence of interaction was observed, we additionally quantified interaction on the additive scale by calculating the Relative Excess Risk due to Interaction (RERI), expressed on the probability scale, to estimate the magnitude of the difference in predicted probabilities attributable to the joint exposure. Finally, we assessed pairwise differences between the four joint exposure groups using Stata’s margins command with the *pwcompare(group)* and *pwcompare(effects)* options; these contrasts are also reported in the Results section.

Box S2: Operationalisation of Covariates

- Age group based on GPPS question “*How old are you?*”. Response options:
  - Under 16
  - 16 to 17
  - 18 to 24
  - 25 to 34
  - 35 to 44
  - 45 to 54
  - 55 to 64
  - 65 to 74
  - 75 to 84
  - 85 or over
- Sex based on GPPS question “*Which of the following best describes you?*”. Response options:
  - Female
  - Male
  - Non-binary
  - Prefer to self-describe
  - Prefer not to say
- Gender identity based on GPPS question “*Is your gender identity the same as the sex you were registered at birth?*”. Response options:
  - Yes
  - No
  - Prefer not to say
- Ethnicity based on GPPS question “*What is your ethnic group?*”. Response options:

A. White

- - - English, Welsh, Scottish, Northern Irish or British
    - Irish
    - Gypsy or Irish Traveller
    - Roma
    - Any other White background

B. Mixed or Multiple ethnic groups

- - - White and Black Caribbean
    - White and Black African
    - White and Asian
    - Any other Mixed or Multiple ethnic background

C. Asian or Asian British

- - - Indian
    - Pakistani
    - Bangladeshi
    - Chinese
    - Any other Asian background

D. Black, Black British, Caribbean or African

- - - Caribbean
    - African
    - Any other Black, Black British, Caribbean or African background

E. Other ethnic group

- - - Arab
    - Any other ethnic group

Ethnicity was included as a covariate, operationalised according to the major ethnic group classifications available in the GPPS dataset.

- Multimorbidity defined by the presence of two or more long-term health conditions in accordance with GPPS question “*Which, if any, of the following long-term conditions do you have?*”.
- Index of Multiple Deprivation (IMD) is the UK government’s measure of relative deprivation. It is a composite index based on scores in seven domains: income, employment, education, health, crime, barriers to housing and services, and living environment.
- Region was included as a covariate, defined using the general practice region coding as London or outside London. GP region options were:
  - East Midlands
  - Eastern
  - London
  - Northeast
  - Northwest
  - Southeast
  - Southwest
  - Wales
  - West Midlands
  - Yorkshire & Humber

| \| **Table S1** \| \| \| \| \| \| \| \| --- \| --- \| --- \| --- \| --- \| --- \| --- \| \| *Survey respondents demographics by presence/absence of mental health condition* \| \| \| \| \| \| \| \|  \| Mental health condition \| \| No mental health condition \| \| Total \| \| \| N \| 128,033 \|  \| 1,149,350 \|  \| 1,277,383 \|  \| \|  \| N (weighted %) \| 95% CI \| N (weighted %) \| 95% CI \| N (weighted %) \| 95% CI \| \| *Sexual orientation* \|  \|  \|  \|  \|  \|  \| \| Heterosexual \| 109 739 (81·0%) \| 80·6 – 81·3 \| 1 059 390 (90·5%) \| 90·41 – 90·6 \| 1 169 129 (89·3%) \| 89·2 – 89·4 \| \| Sexual minority \| 12 052 (13·7%) \| 13·4 – 14·0 \| 35 574 (4·4%) \| 4·4 – 4·5 \| 47 626 (5·6%) \| 5·5 – 5·7 \| \| Prefer not to say \| 6 242 (5·3%) \| 5·2 – 5·5 \| 54 386 (5·1%) \| 5·0 – 5·1 \| 60 628 (5·1%) \| 5·0 – 5·2 \| \|  \|  \|  \|  \|  \|  \|  \| \| *Age* \|  \|  \|  \|  \|  \|  \| \| 16-24 \| 8 376 (14·2%) \| 13·8 – 14·5 \| 37 281 (8·5%) \| 8·4 – 8·6 \| 45 657 (9·2%) \| 9·1 – 9·3 \| \| 25-34 \| 16 648 (22·8%) \| 22·4 – 23·2 \| 84 030 (16·0%) \| 15·9 – 16·2 \| 100 678 (16·9%) \| 16·8 – 17·0 \| \| 35-44 \| 20 816 (19·9%) \| 19·5 – 20·2 \| 133 036 (17·6%) \| 17·5 – 17·7 \| 153 852 (17·9%) \| 17·8 – 18·0 \| \| 45-54 \| 27 816 (19·7%) \| 19·4 – 20·0 \| 176 537 (17·1%) \| 17·0 – 17·2 \| 204 353 (17·5%) \| 17·4 – 17·5 \| \| 55-64 \| 31 914 (15·3%) \| 15·1 – 15·5 \| 247 411 (16·7%) \| 16·6 – 16·8 \| 279 325 (16·5%) \| 16·4 – 16·6 \| \| 65-74 \| 16 125 (5·8%) \| 5·7 – 5·9 \| 258 293 (12·9%) \| 12·8 – 12·9 \| 274 418 (12·0%) \| 11·9 – 12·0 \| \| 75+ \| 6 338 (2·4%) \| 2·3 – 2·4 \| 212 762 (11·2%) \| 11·1 – 11·2 \| 219 100 (10·1%) \| 10·0 – 10·1 \| \|  \|  \|  \|  \|  \|  \|  \| \| *Sex* \|  \|  \|  \|  \|  \|  \| \| Female \| 82 108 (57·8%) \| 57·3 – 58·2 \| 640 359 (50·4%) \| 50·2 – 50·5 \| 722 467 (51·3%) \| 51·2 – 51·4 \| \| Male \| 43 626(39·7%) \| 39·3 – 40·1 \| 496 468 (48·3%) \| 48·2 – 48·4 \| 540 094 (47·2%) \| 47·1 – 47·3 \| \| Non-binary \| 888 (1·3%) \| 1·2 – 1·4 \| 1 680 (0·2%) \| 0·18 – 0·21 \| 2 568 (0·3%) \| 0·3 – 0·4 \| \| Prefer to self-describe \| 317 (0·3%) \| 0·3 – 0·4 \| 1 514 (0·2%) \| 0·16 – 0·18 \| 1 831 (0·2%) \| 0·18 – 0·20 \| \| Prefer not to say \| 1 094 (1·0%) \| 0·9 – 1·1 \| 9 329 (1·0%) \| 0·9 – 1·0 \| 10 423 (1·0%) \| 0·9 – 1·0 \| \|  \|  \|  \|  \|  \|  \|  \| \| *Gender identity* \|  \|  \|  \|  \|  \|  \| \| Cisgender \| 125 279 (96·8%) \| 96·6 – 97·0 \| 1 134 339 (98·4%) \| 98·4 – 98·5 \| 1 259 618 (98·2%) \| 98·2 – 98·3 \| \| Transgender \| 1 431 (1·9%) \| 1·8 – 2·1 \| 5 160 (0·6%) \| 0·5 – 0·6 \| 6 591 (0·7%) \| 0·7 – 0·8 \| \| Prefer not to say \| 1 323 (1·3%) \| 1·2 – 1·4 \| 9 851 (1·0%) \| 1·0 – 1·0 \| 11 174 (1·0%) \| 1·0 – 1·1 \| \|  \|  \|  \|  \|  \|  \|  \| \| *Ethnicity* \|  \|  \|  \|  \|  \|  \| \| White \| 112 934 (88·3%) \| 88·0 – 88·5 \| 954 839 (80·6%) \| 80·5 – 80·7 \| 1 067 773 (81·6%) \| 81·5 – 81·7 \| \| Mixed/multiple ethnic groups \| 2 970 (2·9%) \| 2·7 – 3·0 \| 15 959 (1·9%) \| 1·9 – 2·0 \| 18 929 (2·0%) \| 2·0 – 2·1 \| \| Asian/Asian British \| 6 859 (5·1%) \| 4·9 – 5·2 \| 109 044 (10·7%) \| 10·6 – 10·8 \| 115 903 (10·0%) \| 9·9 – 10·1 \| \| Black/African/Caribbean/Black British \| 3 144 (2·3%) \| 2·2 – 2·4 \| 47 196 (4·4%) \| 4·4 – 4·5 \| 50 340 (4·2%) \| 4·1 – 4·2 \| \| Other ethnic group \| 2 126 (1·5%) \| 1·4 – 1·6 \| 22 312 (2·3%) \| 2·3 – 2·4 \| 24 438 (2·2%) \| 2·2 – 2·3 \| \|  \|  \|  \|  \|  \|  \|  \| \| *Multimorbidity* \|  \|  \|  \|  \|  \|  \| \| No long-term conditions \| – \| – \| 446 496 (49·5%) \| 49·4 – 49·7 \| 446 496 (43·3%) \| 43·2 – 43·4 \| \| No multimorbidity \| 43 044 (40·6%) \| 40·2 – 41·0 \| 356 065 (28·7%) \| 28·6 – 28·8 \| 399 109 (30·2%) \| 30·1 – 30·3 \| \| Multimorbidity \| 84 989 (59·4%) \| 59·0 – 59·8 \| 346 789 (21·8%) \| 21·7 – 21·8 \| 431 778 (26·5%) \| 26·4 – 26·6 \| \|  \|  \|  \|  \|  \|  \|  \| \| *Patient IMD quintile* \|  \|  \|  \|  \|  \|  \| \| Q1 (most deprived) \| 37 113 (28·2%) \| 27·8 – 28·6 \| 215 343 (19·3%) \| 19·2 – 19·4 \| 252 456 (20·4%) \| 20·3 – 20·5 \| \| Q2 \| 28 289 (22·8%) \| 22·5 – 23·2 \| 227 499 (20·4%) \| 20·3 – 20·5 \| 255 788 (20·7%) \| 20·6 – 20·8 \| \| Q3 \| 23 991 (18·8%) \| 18·5 – 19·1 \| 238 887 (20·3%) \| 20·2 – 20·4 \| 262 878 (20·1%) \| 20·0 – 20·2 \| \| Q4 \| 20 980 (16·3%) \| 16·0 – 16·6 \| 239 015 (20·1%) \| 20·0 – 20·2 \| 259 995 (19·6%) \| 19·5 – 19·7 \| \| Q5 (least deprived) \| 17 660 (13·9%) \| 13·6 – 14·1 \| 228 606 (19·8%) \| 19·7 – 19·9 \| 246 266 (19·1%) \| 19·0 – 19·2 \| \|  \|  \|  \|  \|  \|  \|  \| \| *Region* \|  \|  \|  \|  \|  \|  \| \| Outside London \| 109 552 (86·7%) \| 86·4 – 86·9 \| 950 120 (82·6%) \| 82·5 – 82·7 \| 1 059 672 (83·1%) \| 83·0 – 83·2 \| \| London \| 18 481 (13·3%) \| 13·1 – 13·6 \| 199 230 (17·4%) \| 17·3 – 17·5 \| 217 711 (16·9%) \| 16·8 – 17·0 \| |
| --- | --- | --- | --- | --- | --- | --- | --- | --- | --- | --- | --- | --- | --- | --- | --- | --- | --- | --- | --- | --- | --- | --- | --- | --- | --- | --- | --- | --- | --- | --- | --- | --- | --- | --- | --- | --- | --- | --- | --- | --- | --- | --- | --- | --- | --- | --- | --- | --- | --- | --- | --- | --- | --- | --- | --- | --- | --- | --- | --- | --- | --- | --- | --- | --- | --- | --- | --- | --- | --- | --- | --- | --- | --- | --- | --- | --- | --- | --- | --- | --- | --- | --- | --- | --- | --- | --- | --- | --- | --- | --- | --- | --- | --- | --- | --- | --- | --- | --- | --- | --- | --- | --- | --- | --- | --- | --- | --- | --- | --- | --- | --- | --- | --- | --- | --- | --- | --- | --- | --- | --- | --- | --- | --- | --- | --- | --- | --- | --- | --- | --- | --- | --- | --- | --- | --- | --- | --- | --- | --- | --- | --- | --- | --- | --- | --- | --- | --- | --- | --- | --- | --- | --- | --- | --- | --- | --- | --- | --- | --- | --- | --- | --- | --- | --- | --- | --- | --- | --- | --- | --- | --- | --- | --- | --- | --- | --- | --- | --- | --- | --- | --- | --- | --- | --- | --- | --- | --- | --- | --- | --- | --- | --- | --- | --- | --- | --- | --- | --- | --- | --- | --- | --- | --- | --- | --- | --- | --- | --- | --- | --- | --- | --- | --- | --- | --- | --- | --- | --- | --- | --- | --- | --- | --- | --- | --- | --- | --- | --- | --- | --- | --- | --- | --- | --- | --- | --- | --- | --- | --- | --- | --- | --- | --- | --- | --- | --- | --- | --- | --- | --- | --- | --- | --- | --- | --- | --- | --- | --- | --- | --- | --- | --- | --- | --- | --- | --- | --- | --- | --- | --- | --- | --- | --- | --- | --- | --- | --- | --- | --- | --- | --- | --- | --- | --- | --- | --- | --- | --- | --- | --- | --- | --- | --- | --- | --- | --- | --- | --- | --- | --- | --- | --- | --- | --- | --- | --- | --- | --- | --- | --- | --- | --- | --- | --- | --- | --- | --- | --- | --- | --- | --- | --- | --- | --- | --- | --- | --- | --- | --- | --- | --- | --- | --- | --- | --- | --- | --- | --- | --- | --- | --- | --- | --- | --- | --- | --- | --- | --- | --- | --- | --- | --- | --- | --- | --- | --- | --- | --- | --- | --- | --- | --- | --- | --- | --- | --- | --- | --- | --- | --- | --- |

| **Table S2** |  |  |  |  |  |  |  |
| --- | --- | --- | --- | --- | --- | --- | --- |
| *Fully adjusted predicted probabilities for outcomes separate for sexual orientation and mental health condition group (overall main effects)* | | | | | | | |
|  | Sexual orientation | | |  | Mental health condition (MHC) | | |
|  | Heterosexual | Sexual minority |  |  | MHC absent | MHC present |  |
| **Outcome** | Predicted probabilities [95% CIs] | | *p*-value |  | Predicted probabilities [95% CIs] | | *p*-value |
| *Mental health needs recognised by the healthcare professional* (1) | 82·4 [82·2–82·6] | 80·5 [79·8–81·2] | < 0·001 |  | 82·4 [82·2–82·5] | 81·9 [81·5–82·3] | 0·07 |
| *Confidence and trust in healthcare professional at last appointment* (2) | 94·2 [94·1–94·3] | 93·8 [93·5–94·1] | 0·08 |  | 94·3 [94·2–94·4] | 93·0 [92·8–93·2] | < 0·001 |
| *Negative perception of healthcare professional interpersonal skills* (3) | 6·7 [6·6–6·8] | 7·3 [6·9–7·6] | 0·002 |  | 6·6 [6·5–6·7] | 8·2 [8·0–8·4] | < 0·001 |
| *Involvement in care and treatment decisions* (4) | 91·2 [91·1–91·3] | 90·5 [90·1–90·9] | 0·001 |  | 91·3 [91·2–91·4] | 90·3 [90·1–90·5] | < 0·001 |
| *Needs met during the last GP appointment* (5) | 92·2 [92·1–92·3] | 91·8 [91·4–92·2] | 0·05 |  | 92·4 [92·3–92·5] | 91·0 [90·8–91·3] | < 0·001 |
| *Help-seeking prior to GP appointment* (6) | 62·7 [62·6–62·9] | 66·3 [65·7–67·0] | < 0·001 |  | 62·7 [62·5–62·8] | 64·5 [64·1–65·0] | < 0·001 |
| *Note*. Sample size per outcome: (1) 500 618; (2) 1 125 400; (3) 1 088 059; (4) 1 019 397; (5) 1 129 005; (6) 1 138 852. | | | | | | |  |

| **Table S3** | | |  | |  | |  |  | |  |
| --- | --- | --- | --- | --- | --- | --- | --- | --- | --- | --- |
| *Pairwise comparisons of fully adjusted predicted probabilities per outcome* | | |  | |  | |  |  | |  |
| Outcome | Contrast | 95% CI L | | 95% CI U | | z-statistic | | | p-value | |
| *Mental health needs recognised by the HCP* (1) |  |  | |  | |  | | |  | |
| Heterosexual#Yes vs Heterosexual#No | -0·60 | -1·07 | | -0·14 | | -2·54 | | | 0·01 | |
| Sexual minority#No vs Heterosexual#No | -2·40 | -3·33 | | -1·48 | | -5·08 | | | 0·00 | |
| Sexual minority#Yes vs Heterosexual#No | -0·87 | -1·97 | | 0·24 | | -1·54 | | | 0·12 | |
| Sexual minority#No vs Heterosexual#Yes | -1·80 | -2·80 | | -0·80 | | -3·54 | | | 0·00 | |
| Sexual minority#Yes vs Heterosexual#Yes | -0·26 | -1·38 | | 0·85 | | -0·47 | | | 0·64 | |
| Sexual minority#Yes vs Sexual minority#No | 1·54 | 0·15 | | 2·93 | | 2·17 | | | 0·03 | |
|  |  |  | |  | |  | | |  | |
| *Confidence and trust in healthcare professional at last appointment* (2) |  |  | |  | |  | | |  | |
| Heterosexual#Yes vs Heterosexual#No | -1·43 | -1·68 | | -1·18 | | -11·01 | | | 0·00 | |
| Sexual minority#No vs Heterosexual#No | -0·47 | -0·87 | | -0·06 | | -2·26 | | | 0·02 | |
| Sexual minority#Yes vs Heterosexual#No | -1·08 | -1·66 | | -0·50 | | -3·67 | | | 0·00 | |
| Sexual minority#No vs Heterosexual#Yes | 0·96 | 0·51 | | 1·42 | | 4·14 | | | 0·00 | |
| Sexual minority#Yes vs Heterosexual#Yes | 0·35 | -0·24 | | 0·94 | | 1·15 | | | 0·25 | |
| Sexual minority#Yes vs Sexual minority#No | -0·62 | -1·29 | | 0·06 | | -1·78 | | | 0·08 | |
|  |  |  | |  | |  | | |  | |
| *Negative perception of healthcare professional interpersonal skills* (3) |  |  | |  | |  | | |  | |
| Heterosexual#Yes vs Heterosexual#No | 1·79 | 1·51 | | 2·06 | | 12·77 | | | 0·00 | |
| Sexual minority#No vs Heterosexual#No | 0·61 | 0·25 | | 0·97 | | 3·31 | | | 0·00 | |
| Sexual minority#Yes vs Heterosexual#No | 2·52 | 2·02 | | 3·02 | | 9·87 | | | 0·00 | |
| Sexual minority#No vs Heterosexual#Yes | -1·18 | -1·65 | | -0·71 | | -4·91 | | | 0·00 | |
| Sexual minority#Yes vs Heterosexual#Yes | 0·73 | 0·30 | | 1·17 | | 3·32 | | | 0·00 | |
| Sexual minority#Yes vs Sexual minority#No | 1·92 | 1·62 | | 2·21 | | 12·65 | | | 0·00 | |
|  |  |  | |  | |  | | |  | |
| *Involvement in care and treatment decisions* (4) |  |  | |  | |  | | |  | |
| Heterosexual#Yes vs Heterosexual#No | -1·04 | -1·33 | | -0·75 | | -6·96 | | | 0·00 | |
| Sexual minority#No vs Heterosexual#No | -0·59 | -1·01 | | -0·17 | | -2·73 | | | 0·01 | |
| Sexual minority#Yes vs Heterosexual#No | -1·68 | -2·22 | | -1·15 | | -6·19 | | | 0·00 | |
| **Table S3 (continued)** |  |  | |  | |  | | |  | |
| Outcome | Contrast | 95% CI L | | 95% CI U | | z-statistic | | | p-value | |
| *Involvement in care and treatment decisions* (4) |  |  | |  | |  | | |  | |
| Sexual minority#No vs Heterosexual#Yes | 0·45 | -0·08 | | 0·99 | | 1·65 | | | 0·10 | |
| Sexual minority#Yes vs Heterosexual#Yes | -0·65 | -1·11 | | -0·18 | | -2·74 | | | 0·01 | |
| Sexual minority#Yes vs Sexual minority#No | -1·10 | -1·40 | | -0·79 | | -6·98 | | | 0·00 | |
|  |  |  | |  | |  | | |  | |
| *Needs met during the last GP appointment* (5) |  |  | |  | |  | | |  | |
| Heterosexual#Yes vs Heterosexual#No | -1·50 | -1·79 | | -1·20 | | -9·97 | | | 0·00 | |
| Sexual minority#No vs Heterosexual#No | -0·57 | -1·03 | | -0·10 | | -2·40 | | | 0·02 | |
| Sexual minority#Yes vs Heterosexual#No | -1·16 | -1·83 | | -0·49 | | -3·39 | | | 0·00 | |
| Sexual minority#No vs Heterosexual#Yes | 0·93 | 0·41 | | 1·45 | | 3·48 | | | 0·00 | |
| Sexual minority#Yes vs Heterosexual#Yes | 0·34 | -0·35 | | 1·03 | | 0·96 | | | 0·34 | |
| Sexual minority#Yes vs Sexual minority#No | -0·59 | -1·37 | | 0·19 | | -1·48 | | | 0·14 | |
|  |  |  | |  | |  | | |  | |
| *Help-seeking prior to GP appointment* (6) |  |  | |  | |  | | |  | |
| Heterosexual#Yes vs Heterosexual#No | 1·70 | 1·25 | | 2·15 | | 7·41 | | | 0·00 | |
| Sexual minority#No vs Heterosexual#No | 3·36 | 2·69 | | 4·03 | | 9·79 | | | 0·00 | |
| Sexual minority#Yes vs Heterosexual#No | 4·99 | 4·22 | | 5·77 | | 12·67 | | | 0·00 | |
| Sexual minority#No vs Heterosexual#Yes | 1·66 | 0·84 | | 2·49 | | 3·95 | | | 0·00 | |
| Sexual minority#Yes vs Heterosexual#Yes | 3·29 | 2·64 | | 3·95 | | 9·79 | | | 0·00 | |
| Sexual minority#Yes vs Sexual minority#No | 1·63 | 1·20 | | 2·06 | | 7·40 | | | 0·00 | |
| *Note*. Sample size per outcome: (1) 500 618; (2) 1 125 400; (3) 1 088 059; (4) 1 019 397; (5) 1 129 005; (6) 1 138 852 | | | | | | | | | | |

| **Table S4** | | | | |
| --- | --- | --- | --- | --- |
| *Stepwise adjusted logistic regression models with exponentiated coefficients (odds ratio) and 95% confidence intervals for mental health needs recognised outcome* | | | | |
|  | **Model A**:  Main analysis | **Model B**:  Model A + age group + gender + gender identity + ethnicity + survey year | **Model C**:  Model B + health variables | **Model D**:  Model C + sociodem. variables |
| *Sexual orientation* |  |  |  |  |
| Sexual minority | 0⸱72 [0⸱68–0⸱76] | 0⸱82 [0⸱77–0⸱87] | 0⸱83 [0⸱78–0⸱88] | 0⸱85 [0⸱80–0⸱90] |
|  |  |  |  |  |
| *Self-reported mental health condition* |  |  |  |  |
| Yes | 0⸱75 [0⸱73–0⸱77] | 0⸱74 [0⸱72–0⸱76] | 0⸱95 [0⸱92–0⸱98] | 0⸱96 [0⸱93–0⸱99] |
|  |  |  |  |  |
| *Interaction between sexual orientation and self-reported mental health condition* |  |  |  |  |
| Sexual minority#Yes | 1⸱15 [1⸱05–1⸱25] | 1⸱15 [1⸱05–1⸱26] | 1⸱17 [1⸱07–1⸱29] | 1⸱15 [1⸱05–1⸱26] |
|  |  |  |  |  |
| *Age* |  |  |  |  |
| 25-34 | – | 1⸱01 [0⸱96–1⸱06] | 1⸱01 [0⸱96–1⸱06] | 1⸱02 [0⸱97–1⸱08] |
| 35-44 | – | 1⸱05 [1⸱00–1⸱11] | 1⸱08 [1⸱03–1⸱13] | 1⸱09 [1⸱04–1⸱14] |
| 45-54 | – | 1⸱18 [1⸱13–1⸱24] | 1⸱29 [1⸱23–1⸱35] | 1⸱29 [1⸱23–1⸱35] |
| 55-64 | – | 1⸱33 [1⸱26–1⸱39] | 1⸱55 [1⸱47–1⸱63] | 1⸱54 [1⸱46–1⸱62] |
| 65-74 | – | 1⸱49 [1⸱41–1⸱57] | 1⸱87 [1⸱77–1⸱97] | 1⸱84 [1⸱74–1⸱94] |
| 75+ | – | 1⸱73 [1⸱64–1⸱83] | 2⸱34 [2⸱21–2⸱48] | 2⸱27 [2⸱14–2⸱40] |
|  |  |  |  |  |
| *Sex* |  |  |  |  |
| Male | – | 1⸱04 [1⸱02–1⸱06] | 1⸱05 [1⸱03–1⸱08] | 1⸱06 [1⸱03–1⸱08] |
| Non-binary | – | 0⸱88 [0⸱72–1⸱08] | 0⸱90 [0⸱74–1⸱11] | 0⸱90 [0⸱73–1⸱10] |
| Prefer to self-describe | – | 0⸱55 [0⸱43–0⸱70] | 0⸱56 [0⸱44–0⸱72] | 0⸱56 [0⸱44–0⸱71] |
| Prefer not to say | – | 0⸱81 [0⸱69–0⸱94] | 0⸱82 [0⸱70–0⸱96] | 0⸱83 [0⸱71–0⸱97] |
|  |  |  |  |  |
| *Gender identity* |  |  |  |  |
| Transgender | – | 0⸱82 [0⸱71–0⸱94] | 0⸱84 [0⸱73–0⸱96] | 0⸱85 [0⸱74–0⸱97] |
| Prefer not to say | – | 0⸱76 [0⸱63–0⸱92] | 0⸱76 [0⸱63–0⸱91] | 0⸱77 [0⸱64–0⸱93] |
|  |  |  |  |  |
| *Ethnicity* |  |  |  |  |
| Mixed/Multiple ethnic groups | – | 0⸱79 [0⸱73–0⸱85] | 0⸱79 [0⸱73–0⸱85] | 0⸱83 [0⸱76–0⸱89] |
| Asian/Asian British | – | 0⸱64 [0⸱62–0⸱66] | 0⸱62 [0⸱60–0⸱64] | 0⸱66 [0⸱64–0⸱69] |
| Black/African/Caribbean/Black British | – | 0⸱73 [0⸱69–0⸱77] | 0⸱72 [0⸱68–0⸱76] | 0⸱81 [0⸱76–0⸱85] |
| **Table S4 (continued)** |  |  |  |  |
|  | **Model A** | **Model B** | **Model C** | **Model D** |
| *Ethnicity* |  |  |  |  |
| Other ethnic group | – | 0⸱66 [0⸱62–0⸱71] | 0⸱66 [0⸱61–0⸱70] | 0⸱71 [0⸱66–0⸱76] |
|  |  |  |  |  |
| *Survey year* |  |  |  |  |
| 2023 | – | 1⸱06 [1⸱03–1⸱08] | 1⸱06 [1⸱04–1⸱08] | 1⸱06 [1⸱04–1⸱08] |
|  |  |  |  |  |
| *Multimorbidity* |  |  |  |  |
| No multimorbidity | **–** | – | 0⸱80 [0⸱77–0⸱82] | 0⸱80 [0⸱78–0⸱83] |
| Multimorbidity | – | – | 0⸱59 [0⸱57–0⸱61] | 0⸱60 [0⸱58–0⸱62] |
|  |  |  |  |  |
| *Patient IMD quintile* |  |  |  |  |
| Q2 | – | – | – | 1⸱08 [1⸱04–1⸱11] |
| Q3 | – | – | – | 1⸱19 [1⸱15–1⸱23] |
| Q4 | – | – | – | 1⸱25 [1⸱21–1⸱30] |
| Q5 (least deprived) | – | – | – | 1⸱33 [1⸱28–1⸱38] |
|  |  |  |  |  |
| *Region* |  |  |  |  |
| London | – | – | – | 0⸱90 [0⸱87–0⸱93] |
|  |  |  |  |  |
| Observations | 513,970 | 500,896 | 500,896 | 500,618 |

| **Table S5** | | | | |
| --- | --- | --- | --- | --- |
| *Stepwise adjusted logistic regression models with exponentiated coefficients (odds ratio) and 95% confidence intervals for confidence & trust in the HCP outcome* | | | | |
|  | **Model A**:  Main analysis | **Model B**:  Model A + age group + gender + gender identity + ethnicity + survey year | **Model C**:  Model B + health variables | **Model D**:  Model C + sociodem. variables |
| *Sexual orientation* |  |  |  |  |
| Sexual minority | 0⸱67 [0⸱63–0⸱72] | 0⸱86 [0⸱81–0⸱92] | 0⸱88 [0⸱83–0⸱94] | 0⸱91 [0⸱86–0⸱98] |
|  |  |  |  |  |
| *Self-reported mental health condition* |  |  |  |  |
| Yes | 0⸱52 [0⸱51–0⸱54] | 0⸱58 [0⸱56–0⸱60] | 0⸱78 [0⸱75–0⸱81] | 0⸱80 [0⸱77–0⸱83] |
|  |  |  |  |  |
| *Interaction between sexual orientation and self-reported mental health condition* |  |  |  |  |
| Sexual minority#Yes | 1⸱17 [1⸱06–1⸱30] | 1⸱16 [1⸱05–1⸱29] | 1⸱18 [1⸱06–1⸱31] | 1⸱14 [1⸱02–1⸱27] |
|  |  |  |  |  |
| *Age* |  |  |  |  |
| 25-34 | – | 0⸱88 [0⸱84–0⸱93] | 0⸱89 [0⸱84–0⸱94] | 0⸱91 [0⸱86–0⸱96] |
| 35-44 | – | 1⸱01 [0⸱96–1⸱07] | 1⸱05 [0⸱99–1⸱10] | 1⸱06 [1⸱00–1⸱11] |
| 45-54 | – | 1⸱26 [1⸱19–1⸱32] | 1⸱39 [1⸱32–1⸱46] | 1⸱38 [1⸱31–1⸱45] |
| 55-64 | – | 1⸱64 [1⸱56–1⸱73] | 1⸱97 [1⸱86–2⸱08] | 1⸱94 [1⸱84–2⸱05] |
| 65-74 | – | 2⸱23 [2⸱11–2⸱36] | 2⸱92 [2⸱75–3⸱09] | 2⸱81 [2⸱65–2⸱98] |
| 75+ | – | 2⸱74 [2⸱58–2⸱90] | 3⸱89 [3⸱65–4⸱14] | 3⸱67 [3⸱44–3⸱91] |
|  |  |  |  |  |
| *Sex* |  |  |  |  |
| Male | – | 1⸱02 [1⸱00–1⸱05] | 1⸱04 [1⸱01–1⸱06] | 1⸱04 [1⸱01–1⸱06] |
| Non-binary | – | 1⸱07 [0⸱85–1⸱35] | 1⸱10 [0⸱88–1⸱39] | 1⸱10 [0⸱87–1⸱38] |
| Prefer to self-describe | – | 0⸱44 [0⸱35–0⸱56] | 0⸱45 [0⸱35–0⸱57] | 0⸱46 [0⸱36–0⸱59] |
| Prefer not to say | – | 0⸱69 [0⸱59–0⸱82] | 0⸱71 [0⸱60–0⸱84] | 0⸱73 [0⸱61–0⸱86] |
|  |  |  |  |  |
| *Gender identity* |  |  |  |  |
| Transgender | – | 0⸱63 [0⸱54–0⸱74] | 0⸱65 [0⸱55–0⸱76] | 0⸱66 [0⸱57–0⸱77] |
| Prefer not to say | – | 0⸱69 [0⸱58–0⸱82] | 0⸱69 [0⸱58–0⸱82] | 0⸱70 [0⸱59–0⸱83] |
|  |  |  |  |  |
| *Ethnicity* |  |  |  |  |
| Mixed/Multiple ethnic groups | – | 0⸱81 [0⸱75–0⸱88] | 0⸱81 [0⸱75–0⸱88] | 0⸱87 [0⸱81–0⸱95] |
| Asian/Asian British | – | 0⸱68 [0⸱66–0⸱71] | 0⸱67 [0⸱65–0⸱70] | 0⸱75 [0⸱72–0⸱78] |
| Black/African/Caribbean/Black British | – | 1⸱04 [0⸱97–1⸱10] | 1⸱03 [0⸱97–1⸱10] | 1⸱23 [1⸱15–1⸱31] |
| **Table S5 (continued)** |  |  |  |  |
|  | **Model A** | **Model B** | **Model C** | **Model D** |
| *Ethnicity* |  |  |  |  |
| Other ethnic group | – | 0⸱62 [0⸱57–0⸱67] | 0⸱61 [0⸱57–0⸱66] | 0⸱69 [0⸱64–0⸱75] |
|  |  |  |  |  |
| *Survey year* |  |  |  |  |
| 2023 | – | 1⸱00 [0⸱98–1⸱03] | 1⸱01 [0⸱98–1⸱03] | 1⸱00 [0⸱98–1⸱03] |
|  |  |  |  |  |
| *Multimorbidity* |  |  |  |  |
| No multimorbidity | **–** | – | 0⸱76 [0⸱73–0⸱78] | 0⸱76 [0⸱74–0⸱79] |
| Multimorbidity | – | – | 0⸱55 [0⸱53–0⸱57] | 0⸱57 [0⸱55–0⸱59] |
|  |  |  |  |  |
| *Patient IMD quintile* |  |  |  |  |
| Q2 | – | – | – | 1⸱16 [1⸱12–1⸱21] |
| Q3 | – | – | – | 1⸱37 [1⸱32–1⸱42] |
| Q4 | – | – | – | 1⸱48 [1⸱43–1⸱54] |
| Q5 (least deprived) | – | – | – | 1⸱62 [1⸱56–1⸱69] |
|  |  |  |  |  |
| *Region* |  |  |  |  |
| London | – | – | – | 0⸱87 [0⸱84–0⸱90] |
|  |  |  |  |  |
| Observations | 1,151,447 | 1,126,027 | 1,126,027 | 1,125,400 |

| **Table S6** | | | | |
| --- | --- | --- | --- | --- |
| *Stepwise adjusted logistic regression models with exponentiated coefficients (odds ratio) and 95% confidence intervals for negative perception of HCP interpersonal skills outcome* | | | | |
|  | **Model A**:  Main analysis | **Model B**:  Model A + age group + gender + gender identity + ethnicity + survey year | **Model C**:  Model B + health variables | **Model D**:  Model C + sociodem. variables |
| *Sexual orientation* |  |  |  |  |
| Sexual minority | 1⸱50 [1⸱44–1⸱58] | 1⸱16 [1⸱10–1⸱22] | 1⸱13 [1⸱07–1⸱19] | 1⸱10 [1⸱05–1⸱16] |
|  |  |  |  |  |
| *Self-reported mental health condition* |  |  |  |  |
| Yes | 1⸱93 [1⸱87–1⸱99] | 1⸱73 [1⸱67–1⸱78] | 1⸱29 [1⸱25–1⸱34] | 1⸱28 [1⸱23–1⸱32] |
|  |  |  |  |  |
| *Age* |  |  |  |  |
| 25-34 | – | 0⸱95 [0⸱91–1⸱00] | 0⸱95 [0⸱90–0⸱99] | 0⸱93 [0⸱89–0⸱98] |
| 35-44 | – | 0⸱77 [0⸱74–0⸱81] | 0⸱75 [0⸱71–0⸱78] | 0⸱74 [0⸱71–0⸱77] |
| 45-54 | – | 0⸱60 [0⸱57–0⸱63] | 0⸱54 [0⸱52–0⸱57] | 0⸱54 [0⸱52–0⸱57] |
| 55-64 | – | 0⸱46 [0⸱44–0⸱48] | 0⸱38 [0⸱37–0⸱4] | 0⸱39 [0⸱37–0⸱41] |
| 65-74 | – | 0⸱32 [0⸱31–0⸱34] | 0⸱25 [0⸱24–0⸱26] | 0⸱26 [0⸱24–0⸱27] |
| 75+ | – | 0⸱25 [0⸱24–0⸱27] | 0⸱18 [0⸱17–0⸱19] | 0⸱19 [0⸱18–0⸱20] |
|  |  |  |  |  |
| *Sex* |  |  |  |  |
| Male | – | 0⸱96 [0⸱94–0⸱99] | 0⸱95 [0⸱93–0⸱98] | 0⸱95 [0⸱93–0⸱98] |
| Non-binary | – | 0⸱98 [0⸱79–1⸱21] | 0⸱94 [0⸱76–1⸱17] | 0⸱95 [0⸱77–1⸱18] |
| Prefer to self-describe | – | 2⸱11 [1⸱67–2⸱67] | 2⸱07 [1⸱64–2⸱62] | 2⸱03 [1⸱60–2⸱57] |
| Prefer not to say | – | 1⸱35 [1⸱15–1⸱58] | 1⸱33 [1⸱13–1⸱56] | 1⸱30 [1⸱11–1⸱52] |
|  |  |  |  |  |
| *Gender identity* |  |  |  |  |
| Transgender | – | 1⸱17 [1⸱00–1⸱38] | 1⸱13 [0⸱96–1⸱33] | 1⸱12 [0⸱96–1⸱32] |
| Prefer not to say | – | 1⸱27 [1⸱07–1⸱50] | 1⸱27 [1⸱07–1⸱50] | 1⸱25 [1⸱06–1⸱48] |
|  |  |  |  |  |
| *Ethnicity* |  |  |  |  |
| Mixed/Multiple ethnic groups | – | 1⸱18 [1⸱1–1⸱28] | 1⸱18 [1⸱09–1⸱27] | 1⸱10 [1⸱02–1⸱19] |
| Asian/Asian British | – | 1⸱45 [1⸱40–1⸱50] | 1⸱47 [1⸱42–1⸱53] | 1⸱34 [1⸱29–1⸱39] |
| Black/African/Caribbean/Black British | – | 0⸱83 [0⸱78–0⸱88] | 0⸱83 [0⸱79–0⸱89] | 0⸱71 [0⸱67–0⸱76] |
| Other ethnic group | – | 1⸱53 [1⸱42–1⸱65] | 1⸱55 [1⸱44–1⸱67] | 1⸱37 [1⸱27–1⸱48] |
|  |  |  |  |  |
| *Survey year* |  |  |  |  |
| 2023 | – | 0⸱99 [0⸱96–1⸱01] | 0⸱98 [0⸱96–1⸱00] | 0⸱98 [0⸱96–1⸱00] |
|  |  |  |  |  |
| **Table S6 (continued)** |  |  |  |  |
|  | **Model A** | **Model B** | **Model C** | **Model D** |
| *Multimorbidity* |  |  |  |  |
| No multimorbidity | **–** | – | 1⸱30 [1⸱26–1⸱34] | 1⸱30 [1⸱26–1⸱34] |
| Multimorbidity | – | – | 1⸱78 [1⸱72–1⸱84] | 1⸱73 [1⸱68–1⸱79] |
|  |  |  |  |  |
| *Patient IMD quintile* |  |  |  |  |
| Q2 | – | – | – | 0⸱91 [0⸱88–0⸱94] |
| Q3 | – | – | – | 0⸱80 [0⸱77–0⸱83] |
| Q4 | – | – | – | 0⸱75 [0⸱72–0⸱78] |
| Q5 (least deprived) | – | – | – | 0⸱70 [0⸱68–0⸱73] |
|  |  |  |  |  |
| *Region* |  |  |  |  |
| London | – | – | – | 1⸱19 [1⸱16–1⸱23] |
|  |  |  |  |  |
| Observations | 1,111,033 | 1,088,675 | 1,088,675 | 1,088,059 |

| **Table S7** | | | | |
| --- | --- | --- | --- | --- |
| *Stepwise adjusted logistic regression models with exponentiated coefficients (odds ratio) and 95% confidence intervals for involvement in care and treatment decisions outcome* | | | | |
|  | **Model A**:  Main analysis | **Model B**:  Model A + age group + gender + gender identity + ethnicity + survey year | **Model C**:  Model B + health variables | **Model D**:  Model C + sociodem. variables |
| *Sexual orientation* |  |  |  |  |
| Sexual minority | 0⸱72 [0⸱69–0⸱76] | 0⸱88 [0⸱84–0⸱93] | 0⸱90 [0⸱86–0⸱95] | 0⸱93 [0⸱88–0⸱97] |
|  |  |  |  |  |
| *Self-reported mental health condition* |  |  |  |  |
| Yes | 0⸱66 [0⸱64–0⸱67] | 0⸱68 [0⸱66–0⸱70] | 0⸱87 [0⸱84–0⸱90] | 0⸱88 [0⸱86–0⸱91] |
|  |  |  |  |  |
| *Age* |  |  |  |  |
| 25-34 | – | 0⸱98 [0⸱93–1⸱03] | 0⸱98 [0⸱93–1⸱03] | 1⸱00 [0⸱95–1⸱05] |
| 35-44 | – | 1⸱04 [0⸱99–1⸱10] | 1⸱07 [1⸱02–1⸱12] | 1⸱08 [1⸱03–1⸱13] |
| 45-54 | – | 1⸱24 [1⸱18–1⸱3] | 1⸱34 [1⸱28–1⸱41] | 1⸱33 [1⸱27–1⸱40] |
| 55-64 | – | 1⸱48 [1⸱41–1⸱55] | 1⸱71 [1⸱63–1⸱80] | 1⸱68 [1⸱60–1⸱77] |
| 65-74 | – | 1⸱82 [1⸱74–1⸱92] | 2⸱27 [2⸱16–2⸱39] | 2⸱19 [2⸱08–2⸱31] |
| 75+ | – | 2⸱08 [1⸱98–2⸱19] | 2⸱78 [2⸱63–2⸱94] | 2⸱63 [2⸱49–2⸱78] |
|  |  |  |  |  |
| *Sex* |  |  |  |  |
| Male | – | 0⸱87 [0⸱85–0⸱89] | 0⸱88 [0⸱86–0⸱90] | 0⸱88 [0⸱86–0⸱90] |
| Non-binary | – | 0⸱94 [0⸱76–1⸱16] | 0⸱97 [0⸱78–1⸱20] | 0⸱95 [0⸱77–1⸱18] |
| Prefer to self-describe | – | 0⸱58 [0⸱46–0⸱73] | 0⸱59 [0⸱47–0⸱75] | 0⸱61 [0⸱48–0⸱76] |
| Prefer not to say | – | 0⸱65 [0⸱56–0⸱76] | 0⸱66 [0⸱57–0⸱77] | 0⸱68 [0⸱59–0⸱79] |
|  |  |  |  |  |
| *Gender identity* |  |  |  |  |
| Transgender | – | 0⸱69 [0⸱60–0⸱79] | 0⸱71 [0⸱62–0⸱81] | 0⸱72 [0⸱63–0⸱83] |
| Prefer not to say | – | 0⸱64 [0⸱54–0⸱75] | 0⸱63 [0⸱54–0⸱74] | 0⸱65 [0⸱55–0⸱76] |
|  |  |  |  |  |
| *Ethnicity* |  |  |  |  |
| Mixed/Multiple ethnic groups | – | 0⸱79 [0⸱73–0⸱85] | 0⸱79 [0⸱73–0⸱85] | 0⸱84 [0⸱78–0⸱90] |
| Asian/Asian British | – | 0⸱65 [0⸱63–0⸱67] | 0⸱64 [0⸱62–0⸱66] | 0⸱70 [0⸱68–0⸱73] |
| Black/African/Caribbean/Black British | – | 0⸱78 [0⸱74–0⸱82] | 0⸱78 [0⸱74–0⸱82] | 0⸱91 [0⸱86–0⸱96] |
| Other ethnic group | – | 0⸱55 [0⸱51–0⸱58] | 0⸱54 [0⸱51–0⸱58] | 0⸱6 [0⸱56–0⸱65] |
|  |  |  |  |  |
| *Survey year* |  |  |  |  |
| 2023 | – | 1⸱05 [1⸱03–1⸱07] | 1⸱06 [1⸱03–1⸱08] | 1⸱05 [1⸱03–1⸱08] |
|  |  |  |  |  |
|  |  |  |  |  |
| **Table S7 (continued)** |  |  |  |  |
|  | **Model A** | **Model B** | **Model C** | **Model D** |
| *Multimorbidity* |  |  |  |  |
| No multimorbidity | **–** | – | 0⸱81 [0⸱79–0⸱83] | 0⸱82 [0⸱79–0⸱84] |
| Multimorbidity | – | – | 0⸱61 [0⸱59–0⸱63] | 0⸱63 [0⸱61–0⸱65] |
|  |  |  |  |  |
| *Patient IMD quintile* |  |  |  |  |
| Q2 | – | – | – | 1⸱16 [1⸱13–1⸱20] |
| Q3 | – | – | – | 1⸱33 [1⸱29–1⸱38] |
| Q4 | – | – | – | 1⸱42 [1⸱38–1⸱47] |
| Q5 (least deprived) | – | – | – | 1⸱59 [1⸱53–1⸱64] |
|  |  |  |  |  |
| *Region* |  |  |  |  |
| London | – | – | – | 0⸱89 [0⸱87–0⸱92] |
|  |  |  |  |  |
| Observations | 1,043,267 | 1,019,969 | 1,019,969 | 1,019,397 |

| **Table S8** | | | | |
| --- | --- | --- | --- | --- |
| *Stepwise adjusted logistic regression models with exponentiated coefficients (odds ratio) and 95% confidence intervals for needs met outcome* | | | | |
|  | **Model A**:  Main analysis | **Model B**:  Model A + age group + gender + gender identity + ethnicity + survey year | **Model C**:  Model B + health variables | **Model D**:  Model C + sociodem. variables |
| *Sexual orientation* |  |  |  |  |
| Sexual minority | 0⸱68 [0⸱64–0⸱72] | 0⸱87 [0⸱82–0⸱92] | 0⸱89 [0⸱84–0⸱94] | 0⸱92 [0⸱87–0⸱97] |
|  |  |  |  |  |
| *Self-reported mental health condition* |  |  |  |  |
| Yes | 0⸱58 [0⸱56–0⸱60] | 0⸱63 [0⸱61–0⸱65] | 0⸱81 [0⸱79–0⸱84] | 0⸱83 [0⸱80–0⸱86] |
|  |  |  |  |  |
| *Interaction between sexual orientation and self-reported mental health condition* |  |  |  |  |
| Sexual minority#Yes | 1⸱18 [1⸱07–1⸱29] | 1⸱14 [1⸱03–1⸱26] | 1⸱15 [1⸱04–1⸱27] | 1⸱12 [1⸱02–1⸱24] |
|  |  |  |  |  |
| *Age* |  |  |  |  |
| 25-34 | – | 1⸱01 [0⸱96–1⸱06] | 1⸱02 [0⸱97–1⸱07] | 1⸱03 [0⸱98–1⸱08] |
| 35-44 | – | 1⸱13 [1⸱08–1⸱18] | 1⸱16 [1⸱11–1⸱22] | 1⸱17 [1⸱12–1⸱22] |
| 45-54 | – | 1⸱45 [1⸱39–1⸱52] | 1⸱58 [1⸱51–1⸱66] | 1⸱57 [1⸱50–1⸱65] |
| 55-64 | – | 1⸱91 [1⸱83–2⸱00] | 2⸱23 [2⸱12–2⸱33] | 2⸱2 [2⸱10–2⸱31] |
| 65-74 | – | 2⸱67 [2⸱55–2⸱81] | 3⸱35 [3⸱18–3⸱52] | 3⸱25 [3⸱09–3⸱42] |
| 75+ | – | 3⸱11 [2⸱95–3⸱27] | 4⸱18 [3⸱96–4⸱42] | 3⸱99 [3⸱78–4⸱22] |
|  |  |  |  |  |
| *Sex* |  |  |  |  |
| Male | – | 0⸱91 [0⸱89–0⸱93] | 0⸱92 [0⸱90–0⸱94] | 0⸱92 [0⸱90–0⸱94] |
| Non-binary | – | 0⸱90 [0⸱73–1⸱11] | 0⸱93 [0⸱75–1⸱14] | 0⸱93 [0⸱75–1⸱14] |
| Prefer to self-describe | – | 0⸱47 [0⸱38–0⸱59] | 0⸱48 [0⸱38–0⸱60] | 0⸱48 [0⸱38–0⸱60] |
| Prefer not to say | – | 0⸱67 [0⸱57–0⸱77] | 0⸱68 [0⸱58–0⸱78] | 0⸱69 [0⸱60–0⸱80] |
|  |  |  |  |  |
| *Gender identity* |  |  |  |  |
| Transgender | – | 0⸱85 [0⸱74–0⸱99] | 0⸱88 [0⸱76–1⸱01] | 0⸱88 [0⸱77–1⸱02] |
| Prefer not to say | – | 0⸱75 [0⸱64–0⸱89] | 0⸱75 [0⸱64–0⸱89] | 0⸱77 [0⸱65–0⸱91] |
|  |  |  |  |  |
| *Ethnicity* |  |  |  |  |
| Mixed/Multiple ethnic groups | – | 0⸱80 [0⸱75–0⸱86] | 0⸱81 [0⸱75–0⸱87] | 0⸱85 [0⸱79–0⸱92] |
| Asian/Asian British | – | 0⸱68 [0⸱65–0⸱70] | 0⸱67 [0⸱65–0⸱69] | 0⸱72 [0⸱70–0⸱75] |
|  |  |  |  |  |
| **Table S8 (continued)** |  |  |  |  |
|  | **Model A** | **Model B** | **Model C** | **Model D** |
| *Ethnicity* |  |  |  |  |
| Black/African/Caribbean/Black British | – | 0⸱81 [0⸱77–0⸱86] | 0⸱81 [0⸱77–0⸱85] | 0⸱93 [0⸱88–0⸱98] |
| Other ethnic group | – | 0⸱59 [0⸱55–0⸱63] | 0⸱58 [0⸱54–0⸱63] | 0⸱65 [0⸱60–0⸱69] |
|  |  |  |  |  |
| *Survey year* |  |  |  |  |
| 2023 | – | 1⸱01 [0⸱99–1⸱03] | 1⸱02 [0⸱99–1⸱04] | 1⸱01 [0⸱99–1⸱03] |
|  |  |  |  |  |
| *Multimorbidity* |  |  |  |  |
| No multimorbidity | **–** | – | 0⸱79 [0⸱77–0⸱82] | 0⸱80 [0⸱78–0⸱82] |
| Multimorbidity | – | – | 0⸱60 [0⸱58–0⸱62] | 0⸱62 [0⸱60–0⸱64] |
|  |  |  |  |  |
| *Patient IMD quintile* |  |  |  |  |
| Q2 | – | – | – | 1⸱15 [1⸱12–1⸱19] |
| Q3 | – | – | – | 1⸱27 [1⸱23–1⸱32] |
| Q4 | – | – | – | 1⸱37 [1⸱33–1⸱42] |
| Q5 (least deprived) | – | – | – | 1⸱48 [1⸱43–1⸱54] |
|  |  |  |  |  |
| *Region* |  |  |  |  |
| London | – | – | – | 0⸱89 [0⸱87–0⸱92] |
|  |  |  |  |  |
| Observations | 1,155,167 | 1,129,683 | 1,129,683 | 1,129,055 |

| **Table S9** | | | | |
| --- | --- | --- | --- | --- |
| *Stepwise adjusted logistic regression models with exponentiated coefficients (odds ratio) and 95% confidence intervals for help-seeking outcome* | | | | |
|  | **Model A**:  Main analysis | **Model B**:  Model A + age group + gender + gender identity + ethnicity + survey year | **Model C**:  Model B + health variables | **Model D**:  Model C + sociodem. variables |
| *Sexual orientation* |  |  |  |  |
| Sexual minority | 1⸱49 [1⸱45–1⸱53] | 1⸱18 [1⸱15–1⸱22] | 1⸱18 [1⸱14–1⸱21] | 1⸱18 [1⸱14–1⸱21] |
|  |  |  |  |  |
| *Self-reported mental health condition* |  |  |  |  |
| Yes | 1⸱35 [1⸱32–1⸱37] | 1⸱16 [1⸱14–1⸱19] | 1⸱08 [1⸱05–1⸱10] | 1⸱08 [1⸱06–1⸱10] |
|  |  |  |  |  |
| *Age* |  |  |  |  |
| 25-34 | – | 0⸱96 [0⸱93–1⸱00] | 0⸱96 [0⸱93–0⸱99] | 0⸱96 [0⸱93–0⸱99] |
| 35-44 | – | 0⸱83 [0⸱80–0⸱86] | 0⸱82 [0⸱8–0⸱85] | 0⸱82 [0⸱79–0⸱85] |
| 45-54 | – | 0⸱59 [0⸱57–0⸱60] | 0⸱57 [0⸱55–0⸱59] | 0⸱57 [0⸱55–0⸱59] |
| 55-64 | – | 0⸱45 [0⸱43–0⸱46] | 0⸱43 [0⸱41–0⸱44] | 0⸱42 [0⸱41–0⸱44] |
| 65-74 | – | 0⸱36 [0⸱35–0⸱38] | 0⸱34 [0⸱33–0⸱35] | 0⸱34 [0⸱33–0⸱35] |
| 75+ | – | 0⸱31 [0⸱30–0⸱32] | 0⸱28 [0⸱27–0⸱29] | 0⸱28 [0⸱27–0⸱29] |
|  |  |  |  |  |
| *Sex* |  |  |  |  |
| Male | – | 0⸱83 [0⸱82–0⸱83] | 0⸱82 [0⸱81–0⸱83] | 0⸱82 [0⸱81–0⸱83] |
| Non-binary | – | 1⸱00 [0⸱86–1⸱17] | 0⸱99 [0⸱85–1⸱16] | 0⸱99 [0⸱85–1⸱16] |
| Prefer to self-describe | – | 0⸱85 [0⸱71–1⸱02] | 0⸱84 [0⸱70–1⸱02] | 0⸱85 [0⸱71–1⸱02] |
| Prefer not to say | – | 0⸱89 [0⸱80–0⸱99] | 0⸱89 [0⸱80–0⸱99] | 0⸱90 [0⸱81–0⸱99] |
|  |  |  |  |  |
| *Gender identity* |  |  |  |  |
| Transgender | – | 0⸱91 [0⸱82–1⸱01] | 0⸱90 [0⸱82–1⸱00] | 0⸱91 [0⸱82–1⸱00] |
| Prefer not to say | – | 0⸱91 [0⸱81–1⸱03] | 0⸱92 [0⸱81–1⸱03] | 0⸱92 [0⸱81–1⸱04] |
|  |  |  |  |  |
| *Ethnicity* |  |  |  |  |
| Mixed/Multiple ethnic groups | – | 1⸱16 [1⸱11–1⸱22] | 1⸱16 [1⸱11–1⸱22] | 1⸱15 [1⸱1–1⸱21] |
| Asian/Asian British | – | 1⸱36 [1⸱33–1⸱39] | 1⸱36 [1⸱33–1⸱4] | 1⸱36 [1⸱33–1⸱39] |
| Black/African/Caribbean/Black British | – | 0⸱96 [0⸱93–0⸱99] | 0⸱96 [0⸱93–0⸱98] | 0⸱96 [0⸱93–0⸱99] |
| Other ethnic group | – | 1⸱18 [1⸱12–1⸱23] | 1⸱18 [1⸱12–1⸱23] | 1⸱17 [1⸱12–1⸱23] |
|  |  |  |  |  |
| *Survey year* |  |  |  |  |
| 2023 | – | 1⸱07 [1⸱06–1⸱08] | 1⸱07 [1⸱06–1⸱08] | 1⸱07 [1⸱06–1⸱08] |
|  |  |  |  |  |
| **Table S9 (continued)** |  |  |  |  |
|  | **Model A** | **Model B** | **Model C** | **Model D** |
| *Multimorbidity* |  |  |  |  |
| No multimorbidity | **–** | – | 1⸱03 [1⸱01–1⸱04] | 1⸱03 [1⸱02–1⸱05] |
| Multimorbidity | – | – | 1⸱19 [1⸱17–1⸱20] | 1⸱20 [1⸱18–1⸱22] |
|  |  |  |  |  |
| *Patient IMD quintile* |  |  |  |  |
| Q2 | – | – | – | 1⸱04 [1⸱02–1⸱06] |
| Q3 | – | – | – | 1⸱05 [1⸱03–1⸱07] |
| Q4 | – | – | – | 1⸱08 [1⸱06–1⸱10] |
| Q5 (least deprived) | – | – | – | 1⸱11 [1⸱09–1⸱13] |
|  |  |  |  |  |
| *Region* |  |  |  |  |
| London | – | – | – | 1⸱07 [1⸱05–1⸱09] |
|  |  |  |  |  |
| Observations | 1,165,307 | 1,139,493 | 1,139,493 | 1,138,852 |

| **Table S10** |  |  |  |  |
| --- | --- | --- | --- | --- |
| *Fully adjusted logistic regression model assessing the impact of experience quality outcomes on prior help-seeking behaviour* | | | | |
|  | Odds Ratio | 95% CI L | 95% CI U | p-value |
| *Mental health needs recognised* |  |  |  |  |
| No | 1·42 | 1·37 | 1·47 | 0·00 |
|  |  |  |  |  |
| *Confidence & Trust in the HCP* |  |  |  |  |
| No | 0·95 | 0·89 | 1·01 | 0·08 |
|  |  |  |  |  |
| *Involvement in treatment and care decisions* |  |  |  |  |
| No | 1·11 | 1·06 | 1·17 | 0·00 |
|  |  |  |  |  |
| *HCP interpersonal skills* |  |  |  |  |
| Any 'Very poor/Poor' evaluation | 1·29 | 1·22 | 1·36 | 0·00 |
|  |  |  |  |  |
| *Needs met* |  |  |  |  |
| No | 1·30 | 1·24 | 1·36 | 0·00 |
|  |  |  |  |  |
| *Sexual orientation* |  |  |  |  |
| Sexual minority | 1·16 | 1·11 | 1·21 | 0·00 |
|  |  |  |  |  |
| *Self-reported mental health condition* |  |  |  |  |
| Yes | 0·98 | 0·95 | 1·01 | 0·14 |
|  |  |  |  |  |
| *Age* |  |  |  |  |
| 25-34 | 0·92 | 0·87 | 0·97 | 0·00 |
| 35-44 | 0·78 | 0·74 | 0·82 | 0·00 |
| 45-54 | 0·58 | 0·55 | 0·60 | 0·00 |
| 55-64 | 0·46 | 0·43 | 0·48 | 0·00 |
| 65-74 | 0·40 | 0·38 | 0·42 | 0·00 |
| 75+ | 0·37 | 0·35 | 0·39 | 0·00 |
|  |  |  |  |  |
| *Sex* |  |  |  |  |
| Male | 0·81 | 0·79 | 0·82 | 0·00 |
| Non-binary | 0·95 | 0·76 | 1·17 | 0·61 |
| Prefer to self-describe | 0·80 | 0·62 | 1·03 | 0·08 |
| Prefer not to say | 0·86 | 0·74 | 1·01 | 0·07 |
|  |  |  |  |  |
| *Gender identity* |  |  |  |  |
| Transgender | 1·00 | 0·87 | 1·14 | 0·95 |
| Prefer not to say | 0·89 | 0·75 | 1·07 | 0·21 |
|  |  |  |  |  |
| *Ethnicity* |  |  |  |  |
| Mixed/Multiple ethnic groups | 1·10 | 1·02 | 1·19 | 0·01 |
| Asian/Asian British | 1·34 | 1·29 | 1·39 | 0·00 |
| Black/African/Caribbean/Black British | 1·02 | 0·98 | 1·08 | 0·34 |
| Other ethnic group | 1·19 | 1·11 | 1·27 | 0·00 |
|  |  |  |  |  |
| *Multimorbidity* |  |  |  |  |
| No multimorbidity | 1·02 | 0·99 | 1·04 | 0·20 |
| Multimorbidity | 1·14 | 1·11 | 1·17 | 0·00 |
|  |  |  |  |  |
| *Patient IMD quintile* |  |  |  |  |
| Q2 | 1·05 | 1·02 | 1·08 | 0·00 |
| Q3 | 1·10 | 1·07 | 1·14 | 0·00 |
| Q4 | 1·13 | 1·09 | 1·16 | 0·00 |
| Q5 (least deprived) | 1·15 | 1·12 | 1·19 | 0·00 |
| **Table S10 (continued)** |  |  |  |  |
|  | Odds Ratio | 95% CI L | 95% CI U | p-value |
|  |  |  |  |  |
| *Region* |  |  |  |  |
| London | 1·04 | 1·02 | 1·07 | 0·00 |
|  |  |  |  |  |
| *Survey year* |  |  |  |  |
| 2023 | 1·07 | 1·05 | 1·09 | 0·00 |
|  |  |  |  |  |
| *Collection mode* |  |  |  |  |
| Online | 1·18 | 1·16 | 1·21 | 0·00 |
|  |  |  |  |  |
| Observations |  |  |  |  |

| **Table S11** |  |  |  |  |
| --- | --- | --- | --- | --- |
| *Fully adjusted logistic regression model assessing the interaction between sexual orientation and self-reported long-term mental health condition on mental health needs recognised by the HCP* | | | | |
|  | Odds Ratio | 95% CI L | 95% CI U | p-value |
| *Sexual orientation* |  |  |  |  |
| Gay/Lesbian | 0·89 | 0·81 | 0·98 | 0·02 |
| Bisexual | 0·85 | 0·76 | 0·94 | 0·00 |
| Other | 0·82 | 0·74 | 0·90 | 0·00 |
|  |  |  |  |  |
| *Self-reported mental health condition* |  |  |  |  |
| Yes | 0·96 | 0·93 | 0·99 | 0·01 |
|  |  |  |  |  |
| *Interaction between sexual orientation and self-reported mental health condition* |  |  |  |  |
| Gay/Lesbian # Yes | 1·06 | 0·92 | 1·23 | 0·41 |
| Bisexual # Yes | 1·14 | 0·99 | 1·32 | 0·07 |
| Other # Yes | 1·37 | 1·13 | 1·66 | 0·00 |
|  |  |  |  |  |
| *Age* |  |  |  |  |
| 25-34 | 1·03 | 0·98 | 1·09 | 0·26 |
| 35-44 | 1·10 | 1·05 | 1·15 | 0·00 |
| 45-54 | 1·29 | 1·23 | 1·36 | 0·00 |
| 55-64 | 1·53 | 1·46 | 1·61 | 0·00 |
| 65-74 | 1·81 | 1·71 | 1·91 | 0·00 |
| 75+ | 2·18 | 2·06 | 2·31 | 0·00 |
|  |  |  |  |  |
| *Sex* |  |  |  |  |
| Male | 1·05 | 1·03 | 1·08 | 0·00 |
| Non-binary | 0·88 | 0·72 | 1·08 | 0·21 |
| Prefer to self-describe | 0·57 | 0·45 | 0·72 | 0·00 |
| Prefer not to say | 0·84 | 0·71 | 0·98 | 0·02 |
|  |  |  |  |  |
| *Gender identity* |  |  |  |  |
| Transgender | 0·85 | 0·74 | 0·98 | 0·02 |
| Prefer not to say | 0·78 | 0·64 | 0·94 | 0·01 |
|  |  |  |  |  |
| *Ethnicity* |  |  |  |  |
| Mixed/Multiple ethnic groups | 0·83 | 0·77 | 0·90 | 0·00 |
| Asian/Asian British | 0·67 | 0·65 | 0·69 | 0·00 |
| Black/African/Caribbean/Black British | 0·82 | 0·77 | 0·86 | 0·00 |
| Other ethnic group | 0·72 | 0·67 | 0·77 | 0·00 |
|  |  |  |  |  |
| *Multimorbidity* |  |  |  |  |
| No multimorbidity | 0·80 | 0·78 | 0·83 | 0·00 |
| Multimorbidity | 0·60 | 0·58 | 0·62 | 0·00 |
|  |  |  |  |  |
| *Patient IMD quintile* |  |  |  |  |
| Q2 | 1·08 | 1·04 | 1·11 | 0·00 |
| Q3 | 1·19 | 1·15 | 1·23 | 0·00 |
| Q4 | 1·25 | 1·21 | 1·29 | 0·00 |
| Q5 (least deprived) | 1·33 | 1·28 | 1·38 | 0·00 |
|  |  |  |  |  |
| *Region* |  |  |  |  |
| London | 0·90 | 0·87 | 0·93 | 0·00 |
|  |  |  |  |  |
| *Survey year* |  |  |  |  |
| 2023 | 1·07 | 1·04 | 1·09 | 0·00 |
| *Collection mode* |  |  |  |  |
| Online | 0·89 | 0·87 | 0·91 | 0·00 |
| Observations | 500 618 |  |  |  |

| **Table S12** |  |  |  |  |
| --- | --- | --- | --- | --- |
| *Fully adjusted logistic regression model assessing the interaction between sexual orientation and self-reported long-term mental health condition on confidence & trust in the HCP* | | | | |
|  | Odds Ratio | 95% CI L | 95% CI U | p-value |
| *Sexual orientation* |  |  |  |  |
| Gay/Lesbian | 1·00 | 0·91 | 1·09 | 0·94 |
| Bisexual | 1·00 | 0·89 | 1·13 | 0·94 |
| Other | 0·74 | 0·65 | 0·84 | 0·00 |
|  |  |  |  |  |
| *Self-reported mental health condition* |  |  |  |  |
| Yes | 0·80 | 0·77 | 0·83 | 0·00 |
|  |  |  |  |  |
| *Interaction between sexual orientation and self-reported mental health condition* |  |  |  |  |
| Gay/Lesbian # Yes | 1·01 | 0·86 | 1·19 | 0·92 |
| Bisexual # Yes | 1·03 | 0·88 | 1·22 | 0·70 |
| Other # Yes | 1·64 | 1·30 | 2·07 | 0·00 |
|  |  |  |  |  |
| *Age* |  |  |  |  |
| 25-34 | 0·92 | 0·87 | 0·97 | 0·00 |
| 35-44 | 1·07 | 1·02 | 1·13 | 0·01 |
| 45-54 | 1·40 | 1·32 | 1·47 | 0·00 |
| 55-64 | 1·92 | 1·82 | 2·03 | 0·00 |
| 65-74 | 2·71 | 2·56 | 2·88 | 0·00 |
| 75+ | 3·42 | 3·21 | 3·65 | 0·00 |
|  |  |  |  |  |
| *Sex* |  |  |  |  |
| Male | 1·03 | 1·01 | 1·06 | 0·02 |
| Non-binary | 1·07 | 0·85 | 1·34 | 0·57 |
| Prefer to self-describe | 0·48 | 0·38 | 0·62 | 0·00 |
| Prefer not to say | 0·74 | 0·63 | 0·88 | 0·00 |
|  |  |  |  |  |
| *Gender identity* |  |  |  |  |
| Transgender | 0·68 | 0·58 | 0·80 | 0·00 |
| Prefer not to say | 0·72 | 0·60 | 0·85 | 0·00 |
|  |  |  |  |  |
| *Ethnicity* |  |  |  |  |
| Mixed/Multiple ethnic groups | 0·88 | 0·81 | 0·96 | 0·00 |
| Asian/Asian British | 0·76 | 0·73 | 0·79 | 0·00 |
| Black/African/Caribbean/Black British | 1·26 | 1·18 | 1·34 | 0·00 |
| Other ethnic group | 0·71 | 0·65 | 0·77 | 0·00 |
|  |  |  |  |  |
| *Multimorbidity* |  |  |  |  |
| No multimorbidity | 0·76 | 0·74 | 0·79 | 0·00 |
| Multimorbidity | 0·57 | 0·55 | 0·59 | 0·00 |
|  |  |  |  |  |
| *Patient IMD quintile* |  |  |  |  |
| Q2 | 1·16 | 1·12 | 1·20 | 0·00 |
| Q3 | 1·36 | 1·31 | 1·42 | 0·00 |
| Q4 | 1·47 | 1·42 | 1·53 | 0·00 |
| Q5 (least deprived) | 1·61 | 1·55 | 1·68 | 0·00 |
|  |  |  |  |  |
| *Region* |  |  |  |  |
| London | 0·87 | 0·84 | 0·90 | 0·00 |
|  |  |  |  |  |
| *Survey year* |  |  |  |  |
| 2023 | 1·01 | 0·99 | 1·04 | 0·31 |
| *Collection mode* |  |  |  |  |
| Online | 0·81 | 0·79 | 0·83 | 0·00 |
|  |  |  |  |  |
| Observations | 1 125 400 |  |  |  |
| **Table S13** |  |  |  |  |
| *Fully adjusted logistic regression model assessing the interaction between sexual orientation and self-reported long-term mental health condition on perceived negative HCP interpersonal skills* | | | | |
|  | Odds Ratio | 95% CI L | 95% CI U | p-value |
| *Sexual orientation* |  |  |  |  |
| Gay/Lesbian | 1·10 | 1·01 | 1·20 | 0·03 |
| Bisexual | 1·11 | 1·00 | 1·23 | 0·06 |
| Other | 1·02 | 0·89 | 1·18 | 0·78 |
|  |  |  |  |  |
| *Self-reported mental health condition* |  |  |  |  |
| Yes | 1·27 | 1·23 | 1·32 | 0·00 |
|  |  |  |  |  |
| *Interaction between sexual orientation and self-reported mental health condition* |  |  |  |  |
| Gay/Lesbian # Yes | 1·07 | 0·92 | 1·25 | 0·38 |
| Bisexual # Yes | 0·97 | 0·84 | 1·13 | 0·72 |
| Other # Yes | 0·98 | 0·78 | 1·22 | 0·84 |
|  |  |  |  |  |
| *Age* |  |  |  |  |
| 25-34 | 0·92 | 0·88 | 0·96 | 0·00 |
| 35-44 | 0·73 | 0·70 | 0·77 | 0·00 |
| 45-54 | 0·54 | 0·52 | 0·57 | 0·00 |
| 55-64 | 0·39 | 0·37 | 0·41 | 0·00 |
| 65-74 | 0·27 | 0·25 | 0·28 | 0·00 |
| 75+ | 0·20 | 0·19 | 0·21 | 0·00 |
|  |  |  |  |  |
| *Sex* |  |  |  |  |
| Male | 0·95 | 0·93 | 0·98 | 0·00 |
| Non-binary | 0·97 | 0·78 | 1·20 | 0·79 |
| Prefer to self-describe | 1·96 | 1·55 | 2·48 | 0·00 |
| Prefer not to say | 1·29 | 1·10 | 1·51 | 0·00 |
|  |  |  |  |  |
| *Gender identity* |  |  |  |  |
| Transgender | 1·12 | 0·95 | 1·32 | 0·16 |
| Prefer not to say | 1·25 | 1·06 | 1·48 | 0·01 |
|  |  |  |  |  |
| *Ethnicity* |  |  |  |  |
| Mixed/Multiple ethnic groups | 1·09 | 1·01 | 1·18 | 0·03 |
| Asian/Asian British | 1·32 | 1·27 | 1·37 | 0·00 |
| Black/African/Caribbean/Black British | 0·70 | 0·66 | 0·75 | 0·00 |
| Other ethnic group | 1·37 | 1·27 | 1·48 | 0·00 |
|  |  |  |  |  |
| *Multimorbidity* |  |  |  |  |
| No multimorbidity | 1·30 | 1·26 | 1·34 | 0·00 |
| Multimorbidity | 1·74 | 1·68 | 1·80 | 0·00 |
|  |  |  |  |  |
| *Patient IMD quintile* |  |  |  |  |
| Q2 | 0·91 | 0·88 | 0·94 | 0·00 |
| Q3 | 0·80 | 0·78 | 0·83 | 0·00 |
| Q4 | 0·75 | 0·73 | 0·78 | 0·00 |
| Q5 (least deprived) | 0·71 | 0·68 | 0·74 | 0·00 |
|  |  |  |  |  |
| *Region* |  |  |  |  |
| London | 1·19 | 1·16 | 1·23 | 0·00 |
|  |  |  |  |  |
| *Survey year* |  |  |  |  |
| 2023 | 0·98 | 0·95 | 1·00 | 0·04 |
| *Collection mode* |  |  |  |  |
| Online | 1·20 | 1·17 | 1·22 | 0·00 |
|  |  |  |  |  |
| Observations | 1 088 059 |  |  |  |

| **Table S14** |  |  |  |  |
| --- | --- | --- | --- | --- |
| *Fully adjusted logistic regression model assessing the interaction between sexual orientation and self-reported long-term mental health condition on perceived involvement in care decisions* | | | | |
|  | Odds Ratio | 95% CI L | 95% CI U | p-value |
| *Sexual orientation* |  |  |  |  |
| Gay/Lesbian | 1·00 | 0·92 | 1·09 | 0·97 |
| Bisexual | 0·94 | 0·85 | 1·04 | 0·23 |
| Other | 0·72 | 0·64 | 0·80 | 0·00 |
|  |  |  |  |  |
| *Self-reported mental health condition* |  |  |  |  |
| Yes | 0·88 | 0·85 | 0·91 | 0·00 |
|  |  |  |  |  |
| *Interaction between sexual orientation and self-reported mental health condition* |  |  |  |  |
| Gay/Lesbian # Yes | 0·95 | 0·81 | 1·11 | 0·50 |
| Bisexual # Yes | 1·05 | 0·90 | 1·22 | 0·52 |
| Other # Yes | 1·57 | 1·26 | 1·95 | 0·00 |
|  |  |  |  |  |
| *Age* |  |  |  |  |
| 25-34 | 1·01 | 0·96 | 1·06 | 0·76 |
| 35-44 | 1·09 | 1·04 | 1·15 | 0·00 |
| 45-54 | 1·35 | 1·28 | 1·41 | 0·00 |
| 55-64 | 1·68 | 1·60 | 1·76 | 0·00 |
| 65-74 | 2·15 | 2·04 | 2·26 | 0·00 |
| 75+ | 2·51 | 2·38 | 2·66 | 0·00 |
|  |  |  |  |  |
| *Sex* |  |  |  |  |
| Male | 0·88 | 0·86 | 0·89 | 0·00 |
| Non-binary | 0·91 | 0·74 | 1·12 | 0·39 |
| Prefer to self-describe | 0·62 | 0·49 | 0·78 | 0·00 |
| Prefer not to say | 0·69 | 0·60 | 0·81 | 0·00 |
|  |  |  |  |  |
| *Gender identity* |  |  |  |  |
| Transgender | 0·73 | 0·64 | 0·84 | 0·00 |
| Prefer not to say | 0·66 | 0·57 | 0·78 | 0·00 |
|  |  |  |  |  |
| *Ethnicity* |  |  |  |  |
| Mixed/Multiple ethnic groups | 0·84 | 0·78 | 0·91 | 0·00 |
| Asian/Asian British | 0·71 | 0·69 | 0·74 | 0·00 |
| Black/African/Caribbean/Black British | 0·93 | 0·88 | 0·98 | 0·01 |
| Other ethnic group | 0·62 | 0·58 | 0·66 | 0·00 |
|  |  |  |  |  |
| *Multimorbidity* |  |  |  |  |
| No multimorbidity | 0·82 | 0·79 | 0·84 | 0·00 |
| Multimorbidity | 0·63 | 0·61 | 0·65 | 0·00 |
|  |  |  |  |  |
| *Patient IMD quintile* |  |  |  |  |
| Q2 | 1·16 | 1·12 | 1·20 | 0·00 |
| Q3 | 1·33 | 1·29 | 1·37 | 0·00 |
| Q4 | 1·42 | 1·37 | 1·47 | 0·00 |
| Q5 (least deprived) | 1·58 | 1·53 | 1·64 | 0·00 |
|  |  |  |  |  |
| *Region* |  |  |  |  |
| London | 0·89 | 0·87 | 0·92 | 0·00 |
|  |  |  |  |  |
| *Survey year* |  |  |  |  |
| 2023 | 1·06 | 1·04 | 1·08 | 0·00 |
| *Collection mode* |  |  |  |  |
| Online | 0·86 | 0·84 | 0·88 | 0·00 |
|  |  |  |  |  |
| Observations | 1 019 397 |  |  |  |
| **Table S15** |  |  |  |  |
| *Fully adjusted logistic regression model assessing the interaction between sexual orientation and self-reported long-term mental health condition on needs met* | | | | |
|  | Odds Ratio | 95% CI L | 95% CI U | p-value |
| *Sexual orientation* |  |  |  |  |
| Gay/Lesbian | 1·04 | 0·96 | 1·13 | 0·35 |
| Bisexual | 0·90 | 0·81 | 0·99 | 0·03 |
| Other | 0·80 | 0·71 | 0·90 | 0·00 |
|  |  |  |  |  |
| *Self-reported mental health condition* |  |  |  |  |
| Yes | 0·83 | 0·80 | 0·86 | 0·00 |
|  |  |  |  |  |
| *Interaction between sexual orientation and self-reported mental health condition* |  |  |  |  |
| Gay/Lesbian # Yes | 0·92 | 0·79 | 1·07 | 0·30 |
| Bisexual # Yes | 1·21 | 1·05 | 1·41 | 0·01 |
| Other # Yes | 1·34 | 1·08 | 1·65 | 0·01 |
|  |  |  |  |  |
| *Age* |  |  |  |  |
| 25-34 | 1·05 | 1·00 | 1·10 | 0·06 |
| 35-44 | 1·19 | 1·13 | 1·24 | 0·00 |
| 45-54 | 1·59 | 1·51 | 1·66 | 0·00 |
| 55-64 | 2·17 | 2·07 | 2·28 | 0·00 |
| 65-74 | 3·13 | 2·97 | 3·29 | 0·00 |
| 75+ | 3·71 | 3·50 | 3·92 | 0·00 |
|  |  |  |  |  |
| *Sex* |  |  |  |  |
| Male | 0·92 | 0·90 | 0·94 | 0·00 |
| Non-binary | 0·91 | 0·74 | 1·12 | 0·39 |
| Prefer to self-describe | 0·51 | 0·41 | 0·64 | 0·00 |
| Prefer not to say | 0·71 | 0·61 | 0·82 | 0·00 |
|  |  |  |  |  |
| *Gender identity* |  |  |  |  |
| Transgender | 0·91 | 0·79 | 1·05 | 0·20 |
| Prefer not to say | 0·78 | 0·66 | 0·92 | 0·00 |
|  |  |  |  |  |
| *Ethnicity* |  |  |  |  |
| Mixed/Multiple ethnic groups | 0·86 | 0·80 | 0·93 | 0·00 |
| Asian/Asian British | 0·74 | 0·72 | 0·77 | 0·00 |
| Black/African/Caribbean/Black British | 0·95 | 0·90 | 1·01 | 0·08 |
| Other ethnic group | 0·66 | 0·61 | 0·70 | 0·00 |
|  |  |  |  |  |
| *Multimorbidity* |  |  |  |  |
| No multimorbidity | 0·80 | 0·78 | 0·82 | 0·00 |
| Multimorbidity | 0·62 | 0·60 | 0·64 | 0·00 |
|  |  |  |  |  |
| *Patient IMD quintile* |  |  |  |  |
| Q2 | 1·15 | 1·11 | 1·18 | 0·00 |
| Q3 | 1·27 | 1·22 | 1·31 | 0·00 |
| Q4 | 1·37 | 1·32 | 1·41 | 0·00 |
| Q5 (least deprived) | 1·48 | 1·42 | 1·53 | 0·00 |
|  |  |  |  |  |
| *Region* |  |  |  |  |
| London | 0·89 | 0·87 | 0·92 | 0·00 |
|  |  |  |  |  |
| *Survey year* |  |  |  |  |
| 2023 | 1·02 | 1·00 | 1·04 | 0·04 |
| *Collection mode* |  |  |  |  |
| Online | 0·80 | 0·78 | 0·81 | 0·00 |
|  |  |  |  |  |
| Observations | 1 129 055 |  |  |  |

| **Table S16** |  |  |  |  |
| --- | --- | --- | --- | --- |
| *Fully adjusted logistic regression model assessing the interaction between sexual orientation and self-reported long-term mental health condition on help-seeking behaviour* | | | | |
|  | Odds Ratio | 95% CI L | 95% CI U | p-value |
| *Sexual orientation* |  |  |  |  |
| Gay/Lesbian | 1·26 | 1·20 | 1·32 | 0·00 |
| Bisexual | 1·26 | 1·18 | 1·34 | 0·00 |
| Other | 0·95 | 0·88 | 1·02 | 0·16 |
|  |  |  |  |  |
| *Self-reported mental health condition* |  |  |  |  |
| Yes | 1·09 | 1·06 | 1·11 | 0·00 |
|  |  |  |  |  |
| *Interaction between sexual orientation and self-reported mental health condition* |  |  |  |  |
| Gay/Lesbian # Yes | 0·81 | 0·73 | 0·90 | 0·00 |
| Bisexual # Yes | 1·01 | 0·90 | 1·13 | 0·87 |
| Other # Yes | 1·04 | 0·88 | 1·23 | 0·66 |
|  |  |  |  |  |
| *Age* |  |  |  |  |
| 25-34 | 0·95 | 0·92 | 0·98 | 0·00 |
| 35-44 | 0·81 | 0·79 | 0·84 | 0·00 |
| 45-54 | 0·57 | 0·55 | 0·59 | 0·00 |
| 55-64 | 0·43 | 0·42 | 0·44 | 0·00 |
| 65-74 | 0·35 | 0·34 | 0·36 | 0·00 |
| 75+ | 0·30 | 0·29 | 0·31 | 0·00 |
|  |  |  |  |  |
| *Sex* |  |  |  |  |
| Male | 0·82 | 0·81 | 0·83 | 0·00 |
| Non-binary | 1·01 | 0·87 | 1·18 | 0·89 |
| Prefer to self-describe | 0·82 | 0·68 | 0·99 | 0·04 |
| Prefer not to say | 0·89 | 0·80 | 0·99 | 0·03 |
|  |  |  |  |  |
| *Gender identity* |  |  |  |  |
| Transgender | 0·93 | 0·84 | 1·02 | 0·14 |
| Prefer not to say | 0·94 | 0·83 | 1·06 | 0·28 |
|  |  |  |  |  |
| *Ethnicity* |  |  |  |  |
| Mixed/Multiple ethnic groups | 1·15 | 1·09 | 1·21 | 0·00 |
| Asian/Asian British | 1·35 | 1·32 | 1·38 | 0·00 |
| Black/African/Caribbean/Black British | 0·95 | 0·92 | 0·98 | 0·00 |
| Other ethnic group | 1·18 | 1·12 | 1·23 | 0·00 |
|  |  |  |  |  |
| *Multimorbidity* |  |  |  |  |
| No multimorbidity | 1·03 | 1·02 | 1·05 | 0·00 |
| Multimorbidity | 1·20 | 1·19 | 1·22 | 0·00 |
|  |  |  |  |  |
| *Patient IMD quintile* |  |  |  |  |
| Q2 | 1·04 | 1·02 | 1·06 | 0·00 |
| Q3 | 1·05 | 1·04 | 1·07 | 0·00 |
| Q4 | 1·08 | 1·06 | 1·10 | 0·00 |
| Q5 (least deprived) | 1·11 | 1·09 | 1·13 | 0·00 |
|  |  |  |  |  |
| *Region* |  |  |  |  |
| London | 1·07 | 1·05 | 1·08 | 0·00 |
|  |  |  |  |  |
| *Survey year* |  |  |  |  |
| 2023 | 1·06 | 1·05 | 1·08 | 0·00 |
| *Collection mode* |  |  |  |  |
| Online | 1·18 | 1·17 | 1·20 | 0·00 |
|  |  |  |  |  |
| Observations | 1 138 852 |  |  |  |

| **Table S17** |  |  |  |  |  |
| --- | --- | --- | --- | --- | --- |
| *Fully adjusted logistic regression model assessing the interaction between sexual orientation, self-reported long-term mental health condition and age on help-seeking behaviour* | | | | |  |
|  |  |  |  |  |  |
|  | Odds Ratio | 95% CI L | 95% CI U | p-value |  |
| *Sexual orientation* |  |  |  |  |  |
| Sexual minority | 1·40 | 1·24 | 1·57 | 0·00 |  |
|  |  |  |  |  |  |
| *Self-reported mental health condition* |  |  |  |  |  |
| Yes | 1·28 | 1·17 | 1·41 | 0·00 |  |
|  |  |  |  |  |  |
| *Interaction between sexual orientation and self-reported mental health condition* |  |  |  |  |  |
| Sexual minority # Yes | 0·84 | 0·69 | 1·02 | 0·08 |  |
|  |  |  |  |  |  |
| *Age* |  |  |  |  |  |
| 25-34 | 1·01 | 0·97 | 1·05 | 0·73 |  |
| 35-44 | 0·88 | 0·84 | 0·91 | 0·00 |  |
| 45-54 | 0·60 | 0·58 | 0·62 | 0·00 |  |
| 55-64 | 0·44 | 0·43 | 0·46 | 0·00 |  |
| 65-74 | 0·35 | 0·33 | 0·36 | 0·00 |  |
| 75+ | 0·29 | 0·28 | 0·30 | 0·00 |  |
|  |  |  |  |  |  |
| *Interaction between sexual orientation and age* |  |  |  |  |  |
| Sexual minority#25-34 | 0·84 | 0·73 | 0·97 | 0·02 |  |
| Sexual minority#35-44 | 0·79 | 0·69 | 0·90 | 0·00 |  |
| Sexual minority#45-54 | 0·88 | 0·77 | 1·00 | 0·06 |  |
| Sexual minority#55-64 | 0·82 | 0·71 | 0·93 | 0·00 |  |
| Sexual minority#65-74 | 0·86 | 0·74 | 0·99 | 0·04 |  |
| Sexual minority#75+ | 0·84 | 0·72 | 0·98 | 0·03 |  |
|  |  |  |  |  |  |
| *Interaction between self-reported mental health condition and age* |  |  |  |  |  |
| Yes#25-34 | 0·83 | 0·74 | 0·93 | 0·00 |  |
| Yes#35-44 | 0·74 | 0·67 | 0·82 | 0·00 |  |
| Yes#45-54 | 0·79 | 0·71 | 0·87 | 0·00 |  |
| Yes#55-64 | 0·87 | 0·79 | 0·96 | 0·01 |  |
| Yes#65-74 | 1·04 | 0·94 | 1·15 | 0·48 |  |
| Yes#75+ | 1·11 | 0·99 | 1·25 | 0·07 |  |
|  |  |  |  |  |  |
| *Three-way interaction between sexual orientation, self-reported mental health condition and age* |  |  |  |  |  |
| Sexual minority#Yes#25-34 | 1·12 | 0·88 | 1·43 | 0·37 |  |
| Sexual minority#Yes#35-44 | 1·26 | 0·98 | 1·60 | 0·07 |  |
| **Table S17 (continued)** |  |  |  |  |  |
|  | Odds Ratio | 95% CI L | 95% CI U | p-value |  |
| *Three-way interaction between sexual orientation, self-reported mental health condition and age* |  |  |  |  |  |
| Sexual minority#Yes#45-54 | 1·05 | 0·82 | 1·34 | 0·70 |  |
| Sexual minority#Yes#55-64 | 1·12 | 0·87 | 1·43 | 0·38 |  |
| Sexual minority#Yes#65-74 | 1·14 | 0·83 | 1·57 | 0·42 |  |
| Sexual minority#Yes#75+ | 1·30 | 0·78 | 2·16 | 0·32 |  |
|  |  |  |  |  |  |
| *Sex* |  |  |  |  |  |
| Male | 0·82 | 0·81 | 0·83 | 0·00 |  |
| Non-binary | 0·98 | 0·83 | 1·14 | 0·75 |  |
| Prefer to self-describe | 0·85 | 0·70 | 1·02 | 0·08 |  |
| Prefer not to say | 0·90 | 0·81 | 0·99 | 0·04 |  |
|  |  |  |  |  |  |
| *Gender identity* |  |  |  |  |  |
| Transgender | 0·90 | 0·81 | 0·99 | 0·03 |  |
| Prefer not to say | 0·92 | 0·81 | 1·03 | 0·15 |  |
|  |  |  |  |  |  |
| *Ethnicity* |  |  |  |  |  |
| Mixed/Multiple ethnic groups | 1·15 | 1·10 | 1·21 | 0·00 |  |
| Asian/Asian British | 1·36 | 1·33 | 1·39 | 0·00 |  |
| Black/African/Caribbean/Black British | 0·95 | 0·93 | 0·99 | 0·00 |  |
| Other ethnic group | 1·17 | 1·12 | 1·23 | 0·00 |  |
|  |  |  |  |  |  |
| *Multimorbidity* |  |  |  |  |  |
| No multimorbidity | 1·04 | 1·02 | 1·05 | 0·00 |  |
| Multimorbidity | 1·21 | 1·19 | 1·23 | 0·00 |  |
|  |  |  |  |  |  |
| *Patient IMD quintile* |  |  |  |  |  |
| Q2 | 1·04 | 1·02 | 1·06 | 0·00 |  |
| Q3 | 1·05 | 1·03 | 1·07 | 0·00 |  |
| Q4 | 1·08 | 1·06 | 1·10 | 0·00 |  |
| Q5 (least deprived) | 1·11 | 1·09 | 1·13 | 0·00 |  |
|  |  |  |  |  |  |
| *Region* |  |  |  |  |  |
| London | 1·07 | 1·05 | 1·09 | 0·00 |  |
|  |  |  |  |  |  |
| *Survey year* |  |  |  |  |  |
| 2023 | 1·07 | 1·06 | 1·08 | 0·00 |  |
|  |  |  |  |  |  |
| Observations | 1 138 852 |  |  |  |  |

| **Table S18** | | |  | | |  | |  | |  |  |  |
| --- | --- | --- | --- | --- | --- | --- | --- | --- | --- | --- | --- | --- |
| *Fully adjusted logistic regression model assessing the interaction between sexual orientation, self-reported long-term mental health condition and age on mental health needs recognised by the HCP* | | | | | | | | | | |  |  |
|  |  |  |  |  |  |  |  |  |  |  |  | |
|  | Odds Ratio | | | 95% CI L | | | 95% CI U | | p-value | |  | |
| *Sexual orientation* |  | | |  | | |  | |  | |  | |
| Sexual minority | 0·88 | | | 0·74 | | | 1·05 | | 0·16 | |  | |
|  |  | | |  | | |  | |  | |  | |
| *Self-reported mental health condition* |  | | |  | | |  | |  | |  | |
| Yes | 0·90 | | | 0·81 | | | 1·00 | | 0·06 | |  | |
|  |  | | |  | | |  | |  | |  | |
| *Interaction between sexual orientation and self-reported mental health condition* |  | | |  | | |  | |  | |  | |
| Sexual minority # Yes | 1·04 | | | 0·82 | | | 1·32 | | 0·75 | |  | |
|  |  | | |  | | |  | |  | |  | |
| *Age* |  | | |  | | |  | |  | |  | |
| 25-34 | 0·99 | | | 0·93 | | | 1·06 | | 0·80 | |  | |
| 35-44 | 1·01 | | | 0·95 | | | 1·08 | | 0·65 | |  | |
| 45-54 | 1·25 | | | 1·18 | | | 1·33 | | 0·00 | |  | |
| 55-64 | 1·53 | | | 1·43 | | | 1·63 | | 0·00 | |  | |
| 65-74 | 1·86 | | | 1·75 | | | 1·99 | | 0·00 | |  | |
| 75+ | 2·29 | | | 2·14 | | | 2·45 | | 0·00 | |  | |
|  |  | | |  | | |  | |  | |  | |
| *Interaction between sexual orientation and age* |  | | |  | | |  | |  | |  | |
| Sexual minority#25-34 | 0·94 | | | 0·76 | | | 1·17 | | 0·58 | |  | |
| Sexual minority#35-44 | 1·02 | | | 0·83 | | | 1·26 | | 0·82 | |  | |
| Sexual minority#45-54 | 0·93 | | | 0·75 | | | 1·15 | | 0·51 | |  | |
| Sexual minority#55-64 | 0·96 | | | 0·77 | | | 1·20 | | 0·73 | |  | |
| Sexual minority#65-74 | 0·95 | | | 0·74 | | | 1·21 | | 0·68 | |  | |
| Sexual minority#75+ | 0·78 | | | 0·59 | | | 1·01 | | 0·06 | |  | |
|  |  | | |  | | |  | |  | |  | |
| *Interaction between self-reported mental health condition and age* |  | | |  | | |  | |  | |  | |
| Yes#25-34 | 1·08 | | | 0·95 | | | 1·23 | | 0·22 | |  | |
| Yes#35-44 | 1·25 | | | 1·11 | | | 1·41 | | 0·00 | |  | |
| Yes#45-54 | 1·08 | | | 0·96 | | | 1·22 | | 0·21 | |  | |
| Yes#55-64 | 1·01 | | | 0·90 | | | 1·14 | | 0·87 | |  | |
| Yes#65-74 | 0·87 | | | 0·76 | | | 0·99 | | 0·04 | |  | |
| Yes#75+ | 0·79 | | | 0·68 | | | 0·92 | | 0·00 | |  | |
|  |  | | |  | | |  | |  | |  | |
| *Three-way interaction between sexual orientation, self-reported mental health condition and age* |  | | |  | | |  | |  | |  | |
| Sexual minority#Yes#25-34 | 1·16 | | | 0·86 | | | 1·56 | | 0·34 | |  | |
| **Table S18 (continued)** |  | | |  | | |  | |  | |  | |
|  | Odds Ratio | | | 95% CI L | | | 95% CI U | | p-value | |  | |
| *Three-way interaction between sexual orientation, self-reported mental health condition and age* |  | | |  | | |  | |  | |  | |
| Sexual minority#Yes#35-44 | 1·10 | | | 0·81 | | | 1·48 | | 0·55 | |  | |
| Sexual minority#Yes#45-54 | 1·27 | | | 0·93 | | | 1·74 | | 0·14 | |  | |
| Sexual minority#Yes#55-64 | 1·05 | | | 0·75 | | | 1·46 | | 0·78 | |  | |
| Sexual minority#Yes#65-74 | 0·79 | | | 0·51 | | | 1·24 | | 0·31 | |  | |
| Sexual minority#Yes#75+ | 0·75 | | | 0·35 | | | 1·62 | | 0·46 | |  | |
|  |  | | |  | | |  | |  | |  | |
| *Sex* |  | | |  | | |  | |  | |  | |
| Male | 1·06 | | | 1·03 | | | 1·08 | | 0·00 | |  | |
| Non-binary | 0·91 | | | 0·75 | | | 1·12 | | 0·39 | |  | |
| Prefer to self-describe | 0·56 | | | 0·44 | | | 0·71 | | 0·00 | |  | |
| Prefer not to say | 0·83 | | | 0·71 | | | 0·97 | | 0·02 | |  | |
|  |  | | |  | | |  | |  | |  | |
| *Gender identity* |  | | |  | | |  | |  | |  | |
| Transgender | 0·86 | | | 0·75 | | | 0·98 | | 0·03 | |  | |
| Prefer not to say | 0·77 | | | 0·64 | | | 0·93 | | 0·01 | |  | |
|  |  | | |  | | |  | |  | |  | |
| *Ethnicity* |  | | |  | | |  | |  | |  | |
| Mixed/Multiple ethnic groups | 0·83 | | | 0·76 | | | 0·89 | | 0·00 | |  | |
| Asian/Asian British | 0·67 | | | 0·64 | | | 0·69 | | 0·00 | |  | |
| Black/African/Caribbean/Black British | 0·81 | | | 0·77 | | | 0·86 | | 0·00 | |  | |
| Other ethnic group | 0·72 | | | 0·67 | | | 0·77 | | 0·00 | |  | |
|  |  | | |  | | |  | |  | |  | |
| *Multimorbidity* |  | | |  | | |  | |  | |  | |
| No multimorbidity | 0·79 | | | 0·77 | | | 0·82 | | 0·00 | |  | |
| Multimorbidity | 0·60 | | | 0·58 | | | 0·62 | | 0·00 | |  | |
|  |  | | |  | | |  | |  | |  | |
| *Patient IMD quintile* |  | | |  | | |  | |  | |  | |
| Q2 | 1·08 | | | 1·04 | | | 1·11 | | 0·00 | |  | |
| Q3 | 1·19 | | | 1·15 | | | 1·23 | | 0·00 | |  | |
| Q4 | 1·25 | | | 1·21 | | | 1·30 | | 0·00 | |  | |
| Q5 (least deprived) | 1·33 | | | 1·29 | | | 1·38 | | 0·00 | |  | |
|  |  | | |  | | |  | |  | |  | |
| *Region* |  | | |  | | |  | |  | |  | |
| London | 0·90 | | | 0·87 | | | 0·93 | | 0·00 | |  | |
|  |  | | |  | | |  | |  | |  | |
| *Survey year* |  | | |  | | |  | |  | |  | |
| 2023 | 1·06 | | | 1·04 | | | 1·08 | | 0·00 | |  | |
|  |  | | |  | | |  | |  | |  | |
| Observations | 500 618 | | |  | | |  | |  | |  | |
| **Table S19** | |  | | | |  | |  | |  |  |  |
| *Fully adjusted logistic regression model assessing the interaction between sexual orientation, self-reported long-term mental health condition and age on confidence & trust in the HCP* | | | | | | | | | | |  |  |
|  |  |  |  |  |  |  |  |  |  |  |  |  |
|  | | Odds Ratio | | | 95% CI L | | 95% CI U | | p-value | |  |  |
| *Sexual orientation* | |  | | |  | |  | |  | |  |  |
| Sexual minority | | 1·02 | | | 0·85 | | 1·23 | | 0·81 | |  |  |
|  | |  | | |  | |  | |  | |  |  |
| *Self-reported mental health condition* | |  | | |  | |  | |  | |  |  |
| Yes | | 0·71 | | | 0·63 | | 0·80 | | 0·00 | |  |  |
|  | |  | | |  | |  | |  | |  |  |
| *Interaction between sexual orientation and self-reported mental health condition* | |  | | |  | |  | |  | |  |  |
| Sexual minority # Yes | | 0·94 | | | 0·72 | | 1·22 | | 0·63 | |  |  |
|  | |  | | |  | |  | |  | |  |  |
| *Age* | |  | | |  | |  | |  | |  |  |
| 25-34 | | 0·87 | | | 0·81 | | 0·93 | | 0·00 | |  |  |
| 35-44 | | 1·00 | | | 0·93 | | 1·06 | | 0·90 | |  |  |
| 45-54 | | 1·33 | | | 1·25 | | 1·42 | | 0·00 | |  |  |
| 55-64 | | 1·91 | | | 1·79 | | 2·04 | | 0·00 | |  |  |
| 65-74 | | 2·78 | | | 2·59 | | 2·97 | | 0·00 | |  |  |
| 75+ | | 3·65 | | | 3·39 | | 3·92 | | 0·00 | |  |  |
|  | |  | | |  | |  | |  | |  |  |
| *Interaction between sexual orientation and age* | |  | | |  | |  | |  | |  |  |
| Sexual minority#25-34 | | 0·87 | | | 0·69 | | 1·09 | | 0·22 | |  |  |
| Sexual minority#35-44 | | 0·94 | | | 0·75 | | 1·16 | | 0·55 | |  |  |
| Sexual minority#45-54 | | 0·83 | | | 0·66 | | 1·04 | | 0·10 | |  |  |
| Sexual minority#55-64 | | 0·87 | | | 0·68 | | 1·10 | | 0·24 | |  |  |
| Sexual minority#65-74 | | 0·88 | | | 0·65 | | 1·18 | | 0·39 | |  |  |
| Sexual minority#75+ | | 0·65 | | | 0·47 | | 0·90 | | 0·01 | |  |  |
|  | |  | | |  | |  | |  | |  |  |
| *Interaction between self-reported mental health condition and age* | |  | | |  | |  | |  | |  |  |
| Yes#25-34 | | 1·15 | | | 1·00 | | 1·32 | | 0·05 | |  |  |
| Yes#35-44 | | 1·27 | | | 1·11 | | 1·45 | | 0·00 | |  |  |
| Yes#45-54 | | 1·14 | | | 0·99 | | 1·30 | | 0·06 | |  |  |
| Yes#55-64 | | 1·04 | | | 0·91 | | 1·19 | | 0·53 | |  |  |
| Yes#65-74 | | 0·94 | | | 0·80 | | 1·10 | | 0·43 | |  |  |
| Yes#75+ | | 0·80 | | | 0·66 | | 0·97 | | 0·02 | |  |  |
| *Three-way interaction between sexual orientation, self-reported mental health condition and age* | |  | | |  | |  | |  | |  |  |
| Sexual minority#Yes#25-34 | | 1·33 | | | 0·96 | | 1·84 | | 0·09 | |  |  |
| Sexual minority#Yes#35-44 | | 1·10 | | | 0·79 | | 1·54 | | 0·56 | |  |  |
| **Table S19 (continued)** | |  | | |  | |  | |  | |  |  |
|  | | Odds Ratio | | | 95% CI L | | 95% CI U | | p-value | |  |  |
| *Three-way interaction between sexual orientation, self-reported mental health condition and age* | |  | | |  | |  | |  | |  |  |
| Sexual minority#Yes#45-54 | | 1·66 | | | 1·16 | | 2·37 | | 0·06 | |  |  |
| Sexual minority#Yes#55-64 | | 1·37 | | | 0·92 | | 2·04 | | 0·12 | |  |  |
| Sexual minority#Yes#65-74 | | 1·24 | | | 0·69 | | 2·23 | | 0·48 | |  |  |
| Sexual minority#Yes#75+ | | 0·77 | | | 0·36 | | 1·64 | | 0·50 | |  |  |
|  | |  | | |  | |  | |  | |  |  |
| *Sex* | |  | | |  | |  | |  | |  |  |
| Male | | 1·04 | | | 1·01 | | 1·06 | | 0·01 | |  |  |
| Non-binary | | 1·12 | | | 0·89 | | 1·40 | | 0·35 | |  |  |
| Prefer to self-describe | | 0·46 | | | 0·36 | | 0·59 | | 0·00 | |  |  |
| Prefer not to say | | 0·73 | | | 0·61 | | 0·86 | | 0·00 | |  |  |
|  | |  | | |  | |  | |  | |  |  |
| *Gender identity* | |  | | |  | |  | |  | |  |  |
| Transgender | | 0·67 | | | 0·57 | | 0·79 | | 0·00 | |  |  |
| Prefer not to say | | 0·70 | | | 0·59 | | 0·84 | | 0·00 | |  |  |
|  | |  | | |  | |  | |  | |  |  |
| *Ethnicity* | |  | | |  | |  | |  | |  |  |
| Mixed/Multiple ethnic groups | | 0·87 | | | 0·80 | | 0·95 | | 0·00 | |  |  |
| Asian/Asian British | | 0·75 | | | 0·72 | | 0·78 | | 0·00 | |  |  |
| Black/African/Caribbean/Black British | | 1·23 | | | 1·15 | | 1·31 | | 0·00 | |  |  |
| Other ethnic group | | 0·70 | | | 0·64 | | 0·75 | | 0·00 | |  |  |
|  | |  | | |  | |  | |  | |  |  |
| *Multimorbidity* | |  | | |  | |  | |  | |  |  |
| No multimorbidity | | 0·76 | | | 0·74 | | 0·78 | | 0·00 | |  |  |
| Multimorbidity | | 0·57 | | | 0·55 | | 0·59 | | 0·00 | |  |  |
|  | |  | | |  | |  | |  | |  |  |
| *Patient IMD quintile* | |  | | |  | |  | |  | |  |  |
| Q2 | | 1·16 | | | 1·12 | | 1·21 | | 0·00 | |  |  |
| Q3 | | 1·37 | | | 1·32 | | 1·43 | | 0·00 | |  |  |
| Q4 | | 1·48 | | | 1·43 | | 1·54 | | 0·00 | |  |  |
| Q5 (least deprived) | | 1·62 | | | 1·56 | | 1·69 | | 0·00 | |  |  |
|  | |  | | |  | |  | |  | |  |  |
| *Region* | |  | | |  | |  | |  | |  |  |
| London | | 0·87 | | | 0·84 | | 0·90 | | 0·00 | |  |  |
|  | |  | | |  | |  | |  | |  |  |
| *Survey year* | |  | | |  | |  | |  | |  |  |
| 2023 | | 1·00 | | | 0·98 | | 1·03 | | 0·78 | |  |  |
|  | |  | | |  | |  | |  | |  |  |
| Observations | | 1 125 400 | | |  | |  | |  | |  |  |

| **Table S20** |  | |  | |  | |  |  |
| --- | --- | --- | --- | --- | --- | --- | --- | --- |
| *Fully adjusted logistic regression model assessing the interaction between sexual orientation, self-reported long-term mental health condition and age on perceived negative HCP interpersonal skills* | | | | | | | |  |
|  |  |  |  |  |  |  |  |  |
|  | Odds Ratio | 95% CI L | | 95% CI U | | p-value | |  |
| *Sexual orientation* |  |  | |  | |  | |  |
| Sexual minority | 1·00 | 0·85 | | 1·19 | | 0·96 | |  |
|  |  |  | |  | |  | |  |
| *Self-reported mental health condition* |  |  | |  | |  | |  |
| Yes | 1·51 | 1·36 | | 1·68 | | 0·00 | |  |
|  |  |  | |  | |  | |  |
| *Interaction between sexual orientation and self-reported mental health condition* |  |  | |  | |  | |  |
| Sexual minority # Yes | 1·09 | 0·87 | | 1·38 | | 0·45 | |  |
|  |  |  | |  | |  | |  |
| *Age* |  |  | |  | |  | |  |
| 25-34 | 0·98 | 0·93 | | 1·04 | | 0·56 | |  |
| 35-44 | 0·79 | 0·74 | | 0·83 | | 0·00 | |  |
| 45-54 | 0·57 | 0·54 | | 0·60 | | 0·00 | |  |
| 55-64 | 0·40 | 0·38 | | 0·42 | | 0·00 | |  |
| 65-74 | 0·27 | 0·25 | | 0·28 | | 0·00 | |  |
| 75+ | 0·19 | 0·18 | | 0·21 | | 0·00 | |  |
|  |  |  | |  | |  | |  |
| *Interaction between sexual orientation and age* |  |  | |  | |  | |  |
| Sexual minority#25-34 | 1·11 | 0·90 | | 1·36 | | 0·33 | |  |
| Sexual minority#35-44 | 1·13 | 0·93 | | 1·38 | | 0·22 | |  |
| Sexual minority#45-54 | 1·11 | 0·90 | | 1·38 | | 0·33 | |  |
| Sexual minority#55-64 | 1·08 | 0·86 | | 1·36 | | 0·50 | |  |
| Sexual minority#65-74 | 1·14 | 0·85 | | 1·53 | | 0·38 | |  |
| Sexual minority#75+ | 1·50 | 1·06 | | 2·11 | | 0·02 | |  |
|  |  |  | |  | |  | |  |
| *Interaction between self-reported mental health condition and age* |  |  | |  | |  | |  |
| Yes#25-34 | 0·80 | 0·71 | | 0·91 | | 0·00 | |  |
| Yes#35-44 | 0·75 | 0·67 | | 0·85 | | 0·00 | |  |
| Yes#45-54 | 0·83 | 0·74 | | 0·94 | | 0·00 | |  |
| Yes#55-64 | 0·89 | 0·79 | | 1·01 | | 0·07 | |  |
| Yes#65-74 | 0·92 | 0·79 | | 1·07 | | 0·26 | |  |
| Yes#75+ | 1·16 | 0·96 | | 1·41 | | 0·12 | |  |
|  |  |  | |  | |  | |  |
| *Three-way interaction between sexual orientation, self-reported mental health condition and age* |  |  | |  | |  | |  |
| Sexual minority#Yes#25-34 | 0·88 | 0·66 | | 1·19 | | 0·41 | |  |
| Sexual minority#Yes#35-44 | 0·95 | 0·70 | | 1·30 | | 0·75 | |  |
| **Table S20 (continued)** |  |  | |  | |  | |  |
|  | Odds Ratio | 95% CI L | | 95% CI U | | p-value | |  |
| *Three-way interaction between sexual orientation, self-reported mental health condition and age* |  |  | |  | |  | |  |
| Sexual minority#Yes#45-54 | 0·72 | 0·52 | | 1·01 | | 0·06 | |  |
| Sexual minority#Yes#55-64 | 0·99 | 0·68 | | 1·43 | | 0·95 | |  |
| Sexual minority#Yes#65-74 | 0·78 | 0·45 | | 1·35 | | 0·38 | |  |
| Sexual minority#Yes#75+ | 1·19 | 0·51 | | 2·78 | | 0·69 | |  |
|  |  |  | |  | |  | |  |
| *Sex* |  |  | |  | |  | |  |
| Male | 0·95 | 0·93 | | 0·98 | | 0·00 | |  |
| Non-binary | 0·94 | 0·75 | | 1·17 | | 0·56 | |  |
| Prefer to self-describe | 2·03 | 1·60 | | 2·57 | | 0·00 | |  |
| Prefer not to say | 1·30 | 1·11 | | 1·53 | | 0·00 | |  |
|  |  |  | |  | |  | |  |
| *Gender identity* |  |  | |  | |  | |  |
| Transgender | 1·11 | 0·94 | | 1·30 | | 0·21 | |  |
| Prefer not to say | 1·24 | 1·05 | | 1·47 | | 0·01 | |  |
|  |  |  | |  | |  | |  |
| *Ethnicity* |  |  | |  | |  | |  |
| Mixed/Multiple ethnic groups | 1·10 | 1·02 | | 1·19 | | 0·02 | |  |
| Asian/Asian British | 1·34 | 1·29 | | 1·39 | | 0·00 | |  |
| Black/African/Caribbean/Black British | 0·71 | 0·67 | | 0·76 | | 0·00 | |  |
| Other ethnic group | 1·37 | 1·27 | | 1·48 | | 0·00 | |  |
|  |  |  | |  | |  | |  |
| *Multimorbidity* |  |  | |  | |  | |  |
| No multimorbidity | 1·30 | 1·26 | | 1·34 | | 0·00 | |  |
| Multimorbidity | 1·74 | 1·68 | | 1·80 | | 0·00 | |  |
|  |  |  | |  | |  | |  |
| *Patient IMD quintile* |  |  | |  | |  | |  |
| Q2 | 0·91 | 0·88 | | 0·94 | | 0·00 | |  |
| Q3 | 0·80 | 0·77 | | 0·83 | | 0·00 | |  |
| Q4 | 0·75 | 0·72 | | 0·78 | | 0·00 | |  |
| Q5 (least deprived) | 0·70 | 0·68 | | 0·73 | | 0·00 | |  |
|  |  |  | |  | |  | |  |
| *Region* |  |  | |  | |  | |  |
| London | 1·19 | 1·16 | | 1·23 | | 0·00 | |  |
|  |  |  | |  | |  | |  |
| *Survey year* |  |  | |  | |  | |  |
| 2023 | 0·98 | 0·96 | | 1·01 | | 0·13 | |  |
|  |  |  | |  | |  | |  |
| Observations | 1 088 059 |  | |  | |  | |  |

| **Table S21** |  |  |  |  |  |
| --- | --- | --- | --- | --- | --- |
| *Fully adjusted logistic regression model assessing the interaction between sexual orientation, self-reported long-term mental health condition and age on perceived involvement in care and treatment decisions* | | | | |  |
|  |  |  |  |  |  |
|  | Odds Ratio | 95% CI L | 95% CI U | p-value |  |
| *Sexual orientation* |  |  |  |  |  |
| Sexual minority | 0·87 | 0·73 | 1·04 | 0·13 |  |
|  |  |  |  |  |  |
| *Self-reported mental health condition* |  |  |  |  |  |
| Yes | 0·79 | 0·70 | 0·88 | 0·00 |  |
|  |  |  |  |  |  |
| *Interaction between sexual orientation and self-reported mental health condition* |  |  |  |  |  |
| Sexual minority # Yes | 1·12 | 0·87 | 1·44 | 0·37 |  |
|  |  |  |  |  |  |
| *Age* |  |  |  |  |  |
| 25-34 | 0·95 | 0·89 | 1·01 | 0·11 |  |
| 35-44 | 1·03 | 0·97 | 1·09 | 0·30 |  |
| 45-54 | 1·29 | 1·22 | 1·37 | 0·00 |  |
| 55-64 | 1·65 | 1·55 | 1·75 | 0·00 |  |
| 65-74 | 2·16 | 2·03 | 2·29 | 0·00 |  |
| 75+ | 2·60 | 2·44 | 2·77 | 0·00 |  |
|  |  |  |  |  |  |
| *Interaction between sexual orientation and age* |  |  |  |  |  |
| Sexual minority#25-34 | 1·03 | 0·83 | 1·27 | 0·78 |  |
| Sexual minority#35-44 | 1·05 | 0·86 | 1·29 | 0·62 |  |
| Sexual minority#45-54 | 1·00 | 0·81 | 1·24 | 0·99 |  |
| Sexual minority#55-64 | 1·07 | 0·87 | 1·33 | 0·51 |  |
| Sexual minority#65-74 | 1·04 | 0·81 | 1·33 | 0·75 |  |
| Sexual minority#75+ | 0·85 | 0·65 | 1·11 | 0·23 |  |
|  |  |  |  |  |  |
| *Interaction between self-reported mental health condition and age* |  |  |  |  |  |
| Yes#25-34 | 1·20 | 1·05 | 1·37 | 0·01 |  |
| Yes#35-44 | 1·22 | 1·07 | 1·38 | 0·00 |  |
| Yes#45-54 | 1·10 | 0·97 | 1·25 | 0·13 |  |
| Yes#55-64 | 1·06 | 0·94 | 1·21 | 0·35 |  |
| Yes#65-74 | 0·97 | 0·84 | 1·12 | 0·68 |  |
| Yes#75+ | 0·83 | 0·70 | 0·99 | 0·03 |  |
|  |  |  |  |  |  |
| *Three-way interaction between sexual orientation, self-reported mental health condition and age* |  |  |  |  |  |
| Sexual minority#Yes#25-34 | 0·97 | 0·71 | 1·32 | 0·83 |  |
| **Table S21 (continued)** |  |  |  |  |  |
|  | Odds Ratio | 95% CI L | 95% CI U | p-value |  |
| *Three-way interaction between sexual orientation, self-reported mental health condition and age* |  |  |  |  |  |
| Sexual minority#Yes#35-44 | 0·86 | 0·62 | 1·18 | 0·34 |  |
| Sexual minority#Yes#45-54 | 1·28 | 0·91 | 1·80 | 0·16 |  |
| Sexual minority#Yes#55-64 | 1·11 | 0·78 | 1·58 | 0·58 |  |
| Sexual minority#Yes#65-74 | 1·07 | 0·67 | 1·72 | 0·78 |  |
| Sexual minority#Yes#75+ | 0·50 | 0·21 | 1·21 | 0·13 |  |
|  |  |  |  |  |  |
| *Sex* |  |  |  |  |  |
| Male | 0·88 | 0·86 | 0·90 | 0·00 |  |
| Non-binary | 0·95 | 0·77 | 1·17 | 0·64 |  |
| Prefer to self-describe | 0·60 | 0·48 | 0·76 | 0·00 |  |
| Prefer not to say | 0·68 | 0·59 | 0·79 | 0·00 |  |
|  |  |  |  |  |  |
| *Gender identity* |  |  |  |  |  |
| Transgender | 0·72 | 0·63 | 0·83 | 0·00 |  |
| Prefer not to say | 0·65 | 0·55 | 0·76 | 0·00 |  |
|  |  |  |  |  |  |
| *Ethnicity* |  |  |  |  |  |
| Mixed/Multiple ethnic groups | 0·84 | 0·78 | 0·90 | 0·00 |  |
| Asian/Asian British | 0·70 | 0·68 | 0·73 | 0·00 |  |
| Black/African/Caribbean/Black British | 0·91 | 0·86 | 0·96 | 0·00 |  |
| Other ethnic group | 0·61 | 0·57 | 0·65 | 0·00 |  |
|  |  |  |  |  |  |
| *Multimorbidity* |  |  |  |  |  |
| No multimorbidity | 0·81 | 0·79 | 0·83 | 0·00 |  |
| Multimorbidity | 0·63 | 0·61 | 0·65 | 0·00 |  |
|  |  |  |  |  |  |
| *Patient IMD quintile* |  |  |  |  |  |
| Q2 | 1·16 | 1·13 | 1·20 | 0·00 |  |
| Q3 | 1·34 | 1·29 | 1·38 | 0·00 |  |
| Q4 | 1·42 | 1·38 | 1·47 | 0·00 |  |
| Q5 (least deprived) | 1·59 | 1·53 | 1·64 | 0·00 |  |
|  |  |  |  |  |  |
| *Region* |  |  |  |  |  |
| London | 0·89 | 0·87 | 0·92 | 0·00 |  |
|  |  |  |  |  |  |
| *Survey year* |  |  |  |  |  |
| 2023 | 1·05 | 1·03 | 1·08 | 0·00 |  |
|  |  |  |  |  |  |
| Observations | 1 019 397 |  |  |  |  |

| **Table S22**  *Fully adjusted logistic regression model assessing the interaction between sexual orientation, self-reported long-term mental health condition and age on needs met* | | | | |  |
| --- | --- | --- | --- | --- | --- |
|  |  |  |  |  |  |
|  | Odds Ratio | 95% CI L | 95% CI U | p-value |  |
| *Sexual orientation* |  |  |  |  |  |
| Sexual minority | 0·99 | 0·84 | 1·16 | 0·87 |  |
|  |  |  |  |  |  |
| *Self-reported mental health condition* |  |  |  |  |  |
| Yes | 0·74 | 0·67 | 0·83 | 0·00 |  |
|  |  |  |  |  |  |
| *Interaction between sexual orientation and self-reported mental health condition* |  |  |  |  |  |
| Sexual minority # Yes | 1·08 | 0·85 | 1·36 | 0·53 |  |
|  |  |  |  |  |  |
| *Age* |  |  |  |  |  |
| 25-34 | 1·00 | 0·94 | 1·06 | 0·99 |  |
| 35-44 | 1·13 | 1·07 | 1·19 | 0·00 |  |
| 45-54 | 1·54 | 1·45 | 1·62 | 0·00 |  |
| 55-64 | 2·17 | 2·05 | 2·30 | 0·00 |  |
| 65-74 | 3·23 | 3·05 | 3·43 | 0·00 |  |
| 75+ | 4·03 | 3·78 | 4·29 | 0·00 |  |
|  |  |  |  |  |  |
| *Interaction between sexual orientation and age* |  |  |  |  |  |
| Sexual minority#25-34 | 0·89 | 0·73 | 1·08 | 0·22 |  |
| Sexual minority#35-44 | 0·91 | 0·75 | 1·10 | 0·33 |  |
| Sexual minority#45-54 | 0·99 | 0·81 | 1·21 | 0·89 |  |
| Sexual minority#55-64 | 1·02 | 0·82 | 1·26 | 0·88 |  |
| Sexual minority#65-74 | 0·86 | 0·66 | 1·11 | 0·24 |  |
| Sexual minority#75+ | 0·77 | 0·57 | 1·03 | 0·08 |  |
|  |  |  |  |  |  |
| *Interaction between self-reported mental health condition and age* |  |  |  |  |  |
| Yes#25-34 | 1·19 | 1·05 | 1·35 | 0·01 |  |
| Yes#35-44 | 1·24 | 1·10 | 1·40 | 0·00 |  |
| Yes#45-54 | 1·11 | 0·98 | 1·25 | 0·10 |  |
| Yes#55-64 | 1·06 | 0·94 | 1·20 | 0·37 |  |
| Yes#65-74 | 0·98 | 0·85 | 1·14 | 0·84 |  |
| Yes#75+ | 0·71 | 0·60 | 0·85 | 0·00 |  |
|  |  |  |  |  |  |
| *Three-way interaction between sexual orientation, self-reported mental health condition and age* |  |  |  |  |  |
| Sexual minority#Yes#25-34 | 1·09 | 0·82 | 1·47 | 0·55 |  |
| Sexual minority#Yes#35-44 | 0·91 | 0·67 | 1·23 | 0·53 |  |
| **Table S22 (continued)** |  |  |  |  |  |
|  | Odds Ratio | 95% CI L | 95% CI U | p-value |  |
| *Three-way interaction between sexual orientation, self-reported mental health condition and age* |  |  |  |  |  |
| Sexual minority#Yes#45-54 | 1·20 | 0·87 | 1·66 | 0·26 |  |
| Sexual minority#Yes#55-64 | 1·05 | 0·73 | 1·49 | 0·80 |  |
| Sexual minority#Yes#65-74 | 1·42 | 0·82 | 2·45 | 0·21 |  |
| Sexual minority#Yes#75+ | 0·52 | 0·20 | 1·35 | 0·18 |  |
|  |  |  |  |  |  |
| *Sex* |  |  |  |  |  |
| Male | 0·92 | 0·90 | 0·94 | 0·00 |  |
| Non-binary | 0·93 | 0·76 | 1·15 | 0·51 |  |
| Prefer to self-describe | 0·48 | 0·38 | 0·60 | 0·00 |  |
| Prefer not to say | 0·69 | 0·60 | 0·80 | 0·00 |  |
|  |  |  |  |  |  |
| *Gender identity* |  |  |  |  |  |
| Transgender | 0·89 | 0·77 | 1·03 | 0·11 |  |
| Prefer not to say | 0·77 | 0·65 | 0·91 | 0·00 |  |
|  |  |  |  |  |  |
| *Ethnicity* |  |  |  |  |  |
| Mixed/Multiple ethnic groups | 0·85 | 0·79 | 0·92 | 0·00 |  |
| Asian/Asian British | 0·73 | 0·70 | 0·75 | 0·00 |  |
| Black/African/Caribbean/Black British | 0·93 | 0·88 | 0·98 | 0·01 |  |
| Other ethnic group | 0·65 | 0·60 | 0·69 | 0·00 |  |
|  |  |  |  |  |  |
| *Multimorbidity* |  |  |  |  |  |
| No multimorbidity | 0·79 | 0·77 | 0·82 | 0·00 |  |
| Multimorbidity | 0·62 | 0·60 | 0·64 | 0·00 |  |
|  |  |  |  |  |  |
| *Patient IMD quintile* |  |  |  |  |  |
| Q2 | 1·15 | 1·12 | 1·19 | 0·00 |  |
| Q3 | 1·27 | 1·23 | 1·32 | 0·00 |  |
| Q4 | 1·37 | 1·33 | 1·42 | 0·00 |  |
| Q5 (least deprived) | 1·48 | 1·43 | 1·54 | 0·00 |  |
|  |  |  |  |  |  |
| *Region* |  |  |  |  |  |
| London | 0·89 | 0·87 | 0·92 | 0·00 |  |
|  |  |  |  |  |  |
| *Survey year* |  |  |  |  |  |
| 2023 | 1·01 | 0·99 | 1·03 | 0·24 |  |
|  |  |  |  |  |  |
| Observations | 1 129 055 |  |  |  |  |

| **Table S23** |  | |  | |  | |  |  |
| --- | --- | --- | --- | --- | --- | --- | --- | --- |
| *Fully adjusted logistic regression model assessing the interaction between sexual orientation, self-reported long-term mental health condition and ethnicity on help-seeking behaviour* | | | | | | | |  |
|  |  |  |  |  |  |  |  |  |
|  | Odds Ratio | | 95% CI L | | 95% CI U | | p-value |  |
| *Sexual orientation* |  | |  | |  | |  |  |
| Sexual minority | 1·22 | | 1·17 | | 1·27 | | 0·00 |  |
|  |  | |  | |  | |  |  |
| *Self-reported mental health condition* |  | |  | |  | |  |  |
| Yes | 1·10 | | 1·07 | | 1·12 | | 0·00 |  |
|  |  | |  | |  | |  |  |
| *Interaction between sexual orientation and self-reported mental health condition* |  | |  | |  | |  |  |
| Sexual minority # Yes | 0·94 | | 0·87 | | 1·02 | | 0·12 |  |
|  |  | |  | |  | |  |  |
| *Ethnicity* |  | |  | |  | |  |  |
| Mixed/Multiple ethnic groups | 1·16 | | 1·10 | | 1·23 | | 0·00 |  |
| Asian/Asian British | 1·39 | | 1·36 | | 1·42 | | 0·00 |  |
| Black/African/Caribbean/Black British | 0·96 | | 0·93 | | 0·99 | | 0·01 |  |
| Other ethnic group | 1·21 | | 1·14 | | 1·27 | | 0·00 |  |
|  |  | |  | |  | |  |  |
| *Interaction between sexual orientation and ethnicity* |  | |  | |  | |  |  |
| Sexual minority#Mixed/Multiple ethnic groups | 1·18 | | 0·97 | | 1·43 | | 0·09 |  |
| Sexual minority#Asian/Asian British | 0·81 | | 0·72 | | 0·91 | | 0·00 |  |
| Sexual minority#Black/African/Caribbean/Black British | 0·94 | | 0·81 | | 1·09 | | 0·42 |  |
| Sexual minority#Other ethnic group | 0·84 | | 0·71 | | 0·99 | | 0·04 |  |
|  |  | |  | |  | |  |  |
| *Interaction between self-reported mental health condition and ethnicity* |  | |  | |  | |  |  |
| Yes#Mixed/Multiple ethnic groups | 0·92 | | 0·79 | | 1·06 | | 0·26 |  |
| Yes#Asian/Asian British | 0·88 | | 0·79 | | 0·98 | | 0·02 |  |
| Yes#Black/African/Caribbean/Black British | 1·05 | | 0·93 | | 1·18 | | 0·43 |  |
| Yes#Other ethnic group | 0·87 | | 0·73 | | 1·04 | | 0·13 |  |
|  |  | |  | |  | |  |  |
| *Three-way interaction between sexual orientation, self-reported mental health condition and ethnicity* |  | |  | |  | |  |  |
| Sexual minority#Yes#Mixed/Multiple ethnic groups | 0·74 | | 0·52 | | 1·07 | | 0·11 |  |
| Sexual minority#Yes#Asian/Asian British | 1·01 | | 0·70 | | 1·45 | | 0·96 |  |
| Sexual minority#Yes#Black/African/Caribbean/Black British | 1·21 | | 0·74 | | 1·97 | | 0·46 |  |
| Sexual minority#Yes#Other ethnic group | 1·07 | | 0·67 | | 1·71 | | 0·77 |  |
|  |  | |  | |  | |  |  |
| *Sex* |  | |  | |  | |  |  |
| Male | 0·82 | | 0·81 | | 0·83 | | 0·00 |  |
| **Table S23 (continued)** |  | |  | |  | |  |  |
|  | Odds Ratio | | 95% CI L | | 95% CI U | | p-value |  |
| *Sex* |  | |  | |  | |  |  |
| Non-binary | 0·99 | | 0·85 | | 1·16 | | 0·93 |  |
| Prefer to self-describe | 0·85 | | 0·71 | | 1·02 | | 0·08 |  |
| Prefer not to say | 0·90 | | 0·81 | | 1·00 | | 0·04 |  |
|  |  | |  | |  | |  |  |
| *Gender identity* |  | |  | |  | |  |  |
| Transgender | 0·92 | | 0·83 | | 1·01 | | 0·09 |  |
| Prefer not to say | 0·93 | | 0·82 | | 1·05 | | 0·22 |  |
|  |  | |  | |  | |  |  |
| *Age* |  | |  | |  | |  |  |
| 25-34 | 0·96 | | 0·93 | | 0·99 | | 0·02 |  |
| 35-44 | 0·82 | | 0·79 | | 0·85 | | 0·00 |  |
| 45-54 | 0·57 | | 0·55 | | 0·59 | | 0·00 |  |
| 55-64 | 0·43 | | 0·41 | | 0·44 | | 0·00 |  |
| 65-74 | 0·34 | | 0·33 | | 0·35 | | 0·00 |  |
| 75+ | 0·28 | | 0·27 | | 0·29 | | 0·00 |  |
|  |  | |  | |  | |  |  |
| *Multimorbidity* |  | |  | |  | |  |  |
| No multimorbidity | 1·03 | | 1·02 | | 1·05 | | 0·00 |  |
| Multimorbidity | 1·20 | | 1·18 | | 1·22 | | 0·00 |  |
|  |  | |  | |  | |  |  |
| *Patient IMD quintile* |  | |  | |  | |  |  |
| Q2 | 1·04 | | 1·02 | | 1·06 | | 0·00 |  |
| Q3 | 1·05 | | 1·03 | | 1·07 | | 0·00 |  |
| Q4 | 1·08 | | 1·06 | | 1·10 | | 0·00 |  |
| Q5 (least deprived) | 1·11 | | 1·09 | | 1·13 | | 0·00 |  |
|  |  | |  | |  | |  |  |
| *Region* |  | |  | |  | |  |  |
| London | 1·07 | | 1·05 | | 1·09 | | 0·00 |  |
|  |  | |  | |  | |  |  |
| *Survey year* |  | |  | |  | |  |  |
| 2023 | 1·07 | | 1·06 | | 1·08 | | 0·00 |  |
|  |  | |  | |  | |  |  |
| Observations | 1 138 852 |  | |  |  |  |  |  |

| **Table S24** |  |  |  |  |  |
| --- | --- | --- | --- | --- | --- |
| *Fully adjusted logistic regression model assessing the interaction between sexual orientation, self-reported long-term mental health condition and ethnicity on mental health needs recognised by the HCP* | | | | |  |
|  |  |  |  |  |  |
|  | Odds Ratio | 95% CI L | 95% CI U | p-value |  |
| *Sexual orientation* |  |  |  |  |  |
| Sexual minority | 0·81 | 0·75 | 0·86 | 0·00 |  |
|  |  |  |  |  |  |
| *Self-reported mental health condition* |  |  |  |  |  |
| Yes | 0·93 | 0·90 | 0·96 | 0·00 |  |
|  |  |  |  |  |  |
| *Interaction between sexual orientation and self-reported mental health condition* |  |  |  |  |  |
| Sexual minority # Yes | 1·19 | 1·08 | 1·33 | 0·00 |  |
|  |  |  |  |  |  |
| *Ethnicity* |  |  |  |  |  |
| Mixed/Multiple ethnic groups | 0·74 | 0·68 | 0·82 | 0·00 |  |
| Asian/Asian British | 0·64 | 0·62 | 0·67 | 0·00 |  |
| Black/African/Caribbean/Black British | 0·79 | 0·74 | 0·84 | 0·00 |  |
| Other ethnic group | 0·65 | 0·60 | 0·71 | 0·00 |  |
|  |  |  |  |  |  |
| *Interaction between sexual orientation and ethnicity* |  |  |  |  |  |
| Sexual minority#Mixed/Multiple ethnic groups | 1·44 | 1·05 | 1·99 | 0·03 |  |
| Sexual minority#Asian/Asian British | 1·28 | 1·10 | 1·49 | 0·00 |  |
| Sexual minority#Black/African/Caribbean/Black British | 0·78 | 0·62 | 0·98 | 0·04 |  |
| Sexual minority#Other ethnic group | 1·37 | 1·06 | 1·76 | 0·01 |  |
|  |  |  |  |  |  |
| *Interaction between self-reported mental health condition and ethnicity* |  |  |  |  |  |
| Yes#Mixed/Multiple ethnic groups | 1·28 | 1·06 | 1·55 | 0·01 |  |
| Yes#Asian/Asian British | 1·08 | 0·98 | 1·20 | 0·13 |  |
| Yes#Black/African/Caribbean/Black British | 1·33 | 1·14 | 1·56 | 0·00 |  |
| Yes#Other ethnic group | 1·36 | 1·11 | 1·66 | 0·00 |  |
|  |  |  |  |  |  |
| *Three-way interaction between sexual orientation, self-reported mental health condition and ethnicity* |  |  |  |  |  |
| Sexual minority#Yes#Mixed/Multiple ethnic groups | 0·70 | 0·44 | 1·10 | 0·12 |  |
| Sexual minority#Yes#Asian/Asian British | 0·98 | 0·66 | 1·46 | 0·91 |  |
| Sexual minority#Yes#Black/African/Caribbean/Black British | 1·39 | 0·80 | 2·40 | 0·24 |  |
| Sexual minority#Yes#Other ethnic group | 0·88 | 0·52 | 1·48 | 0·62 |  |
|  |  |  |  |  |  |
| *Sex* |  |  |  |  |  |
| Male | 1·06 | 1·03 | 1·08 | 0·00 |  |
| **Table S24 (continued)** |  |  |  |  |  |
|  | Odds Ratio | 95% CI L | 95% CI U | p-value |  |
| *Sex* |  |  |  |  |  |
| Non-binary | 0·90 | 0·73 | 1·10 | 0·31 |  |
| Prefer to self-describe | 0·55 | 0·43 | 0·71 | 0·00 |  |
| Prefer not to say | 0·83 | 0·71 | 0·97 | 0·02 |  |
|  |  |  |  |  |  |
| *Gender identity* |  |  |  |  |  |
| Transgender | 0·85 | 0·74 | 0·97 | 0·02 |  |
| Prefer not to say | 0·77 | 0·63 | 0·92 | 0·01 |  |
|  |  |  |  |  |  |
| *Age* |  |  |  |  |  |
| 25-34 | 1·02 | 0·97 | 1·07 | 0·45 |  |
| 35-44 | 1·09 | 1·03 | 1·14 | 0·00 |  |
| 45-54 | 1·28 | 1·22 | 1·35 | 0·00 |  |
| 55-64 | 1·53 | 1·45 | 1·61 | 0·00 |  |
| 65-74 | 1·83 | 1·73 | 1·93 | 0·00 |  |
| 75+ | 2·24 | 2·12 | 2·38 | 0·00 |  |
|  |  |  |  |  |  |
| *Multimorbidity* |  |  |  |  |  |
| No multimorbidity | 0·80 | 0·78 | 0·83 | 0·00 |  |
| Multimorbidity | 0·60 | 0·58 | 0·62 | 0·00 |  |
|  |  |  |  |  |  |
| *Patient IMD quintile* |  |  |  |  |  |
| Q2 | 1·08 | 1·04 | 1·11 | 0·00 |  |
| Q3 | 1·19 | 1·15 | 1·23 | 0·00 |  |
| Q4 | 1·25 | 1·21 | 1·29 | 0·00 |  |
| Q5 (least deprived) | 1·33 | 1·28 | 1·38 | 0·00 |  |
|  |  |  |  |  |  |
| *Region* |  |  |  |  |  |
| London | 0·90 | 0·87 | 0·93 | 0·00 |  |
|  |  |  |  |  |  |
| *Survey year* |  |  |  |  |  |
| 2023 | 1·06 | 1·04 | 1·08 | 0·00 |  |
|  |  |  |  |  |  |
| Observations | 500 618 |  |  |  |  |

| **Table S25** |  | |  | |  | |  |  |
| --- | --- | --- | --- | --- | --- | --- | --- | --- |
| *Fully adjusted logistic regression model assessing the interaction between sexual orientation, self-reported long-term mental health condition and ethnicity on confidence & trust in the HCP* | | | | | | | |  |
|  |  |  |  |  |  |  |  |  |
|  | Odds Ratio | | 95% CI L | | 95% CI U | | p-value |  |
| *Sexual orientation* |  | |  | |  | |  |  |
| Sexual minority | 0·94 | | 0·87 | | 1·01 | | 0·08 |  |
|  |  | |  | |  | |  |  |
| *Self-reported mental health condition* |  | |  | |  | |  |  |
| Yes | 0·80 | | 0·77 | | 0·83 | | 0·00 |  |
|  |  | |  | |  | |  |  |
| *Interaction between sexual orientation and self-reported mental health condition* |  | |  | |  | |  |  |
| Sexual minority # Yes | 1·08 | | 0·96 | | 1·21 | | 0·22 |  |
|  |  | |  | |  | |  |  |
| *Ethnicity* |  | |  | |  | |  |  |
| Mixed/Multiple ethnic groups | 0·82 | | 0·75 | | 0·90 | | 0·00 |  |
| Asian/Asian British | 0·74 | | 0·71 | | 0·78 | | 0·00 |  |
| Black/African/Caribbean/Black British | 1·27 | | 1·18 | | 1·36 | | 0·00 |  |
| Other ethnic group | 0·71 | | 0·65 | | 0·77 | | 0·00 |  |
|  |  | |  | |  | |  |  |
| *Interaction between sexual orientation and ethnicity* |  | |  | |  | |  |  |
| Sexual minority#Mixed/Multiple ethnic groups | 1·32 | | 0·96 | | 1·81 | | 0·09 |  |
| Sexual minority#Asian/Asian British | 1·07 | | 0·86 | | 1·34 | | 0·53 |  |
| Sexual minority#Black/African/Caribbean/Black British | 0·67 | | 0·49 | | 0·92 | | 0·01 |  |
| Sexual minority#Other ethnic group | 0·68 | | 0·51 | | 0·91 | | 0·01 |  |
|  |  | |  | |  | |  |  |
| *Interaction between self-reported mental health condition and ethnicity* |  | |  | |  | |  |  |
| Yes#Mixed/Multiple ethnic groups | 1·17 | | 0·94 | | 1·46 | | 0·17 |  |
| Yes#Asian/Asian British | 0·95 | | 0·84 | | 1·08 | | 0·44 |  |
| Yes#Black/African/Caribbean/Black British | 0·90 | | 0·74 | | 1·09 | | 0·27 |  |
| Yes#Other ethnic group | 1·13 | | 0·89 | | 1·43 | | 0·31 |  |
|  |  | |  | |  | |  |  |
| *Three-way interaction between sexual orientation, self-reported mental health condition and ethnicity* |  | |  | |  | |  |  |
| Sexual minority#Yes#Mixed/Multiple ethnic groups | 0·78 | | 0·48 | | 1·27 | | 0·33 |  |
| Sexual minority#Yes#Asian/Asian British | 1·37 | | 0·84 | | 2·21 | | 0·21 |  |
| Sexual minority#Yes#Black/African/Caribbean/Black British | 2·02 | | 0·99 | | 4·12 | | 0·05 |  |
| Sexual minority#Yes#Other ethnic group | 2·03 | | 1·03 | | 3·98 | | 0·04 |  |
|  |  | |  | |  | |  |  |
| *Sex* |  | |  | |  | |  |  |
| Male | 1·04 | | 1·01 | | 1·06 | | 0·01 |  |
| **Table S25 (continued)** |  | |  | |  | |  |  |
|  | Odds Ratio | | 95% CI L | | 95% CI U | | p-value |  |
| *Sex* |  | |  | |  | |  |  |
| Non-binary | 1·09 | | 0·87 | | 1·37 | | 0·46 |  |
| Prefer to self-describe | 0·46 | | 0·36 | | 0·59 | | 0·00 |  |
| Prefer not to say | 0·72 | | 0·61 | | 0·86 | | 0·00 |  |
|  |  | |  | |  | |  |  |
| *Gender identity* |  | |  | |  | |  |  |
| Transgender | 0·67 | | 0·57 | | 0·78 | | 0·00 |  |
| Prefer not to say | 0·70 | | 0·59 | | 0·84 | | 0·00 |  |
|  |  | |  | |  | |  |  |
| *Age* |  | |  | |  | |  |  |
| 25-34 | 0·91 | | 0·86 | | 0·96 | | 0·00 |  |
| 35-44 | 1·06 | | 1·00 | | 1·11 | | 0·04 |  |
| 45-54 | 1·38 | | 1·31 | | 1·46 | | 0·00 |  |
| 55-64 | 1·94 | | 1·84 | | 2·05 | | 0·00 |  |
| 65-74 | 2·81 | | 2·65 | | 2·98 | | 0·00 |  |
| 75+ | 3·67 | | 3·44 | | 3·91 | | 0·00 |  |
|  |  | |  | |  | |  |  |
| *Multimorbidity* |  | |  | |  | |  |  |
| No multimorbidity | 0·76 | | 0·74 | | 0·79 | | 0·00 |  |
| Multimorbidity | 0·57 | | 0·55 | | 0·59 | | 0·00 |  |
|  |  | |  | |  | |  |  |
| *Patient IMD quintile* |  | |  | |  | |  |  |
| Q2 | 1·16 | | 1·12 | | 1·21 | | 0·00 |  |
| Q3 | 1·37 | | 1·32 | | 1·42 | | 0·00 |  |
| Q4 | 1·48 | | 1·43 | | 1·54 | | 0·00 |  |
| Q5 (least deprived) | 1·62 | | 1·56 | | 1·69 | | 0·00 |  |
|  |  | |  | |  | |  |  |
| *Region* |  | |  | |  | |  |  |
| London | 0·87 | | 0·84 | | 0·90 | | 0·00 |  |
|  |  | |  | |  | |  |  |
| *Survey year* |  | |  | |  | |  |  |
| 2023 | 1·00 | | 0·98 | | 1·03 | | 0·77 |  |
|  |  | |  | |  | |  |  |
| Observations | 1 125 400 |  | |  |  |  |  |  |

| **Table S26** |  |  |  |  |  |
| --- | --- | --- | --- | --- | --- |
| *Fully adjusted logistic regression model assessing the interaction between sexual orientation, self-reported long-term mental health condition and ethnicity on perceived negative HCP interpersonal skills* | | | | |  |
|  |  |  |  |  |  |
|  | Odds Ratio | 95% CI L | 95% CI U | p-value |  |
| *Sexual orientation* |  |  |  |  |  |
| Sexual minority | 1·15 | 1·08 | 1·23 | 0·00 |  |
|  |  |  |  |  |  |
| *Self-reported mental health condition* |  |  |  |  |  |
| Yes | 1·26 | 1·21 | 1·31 | 0·00 |  |
|  |  |  |  |  |  |
| *Interaction between sexual orientation and self-reported mental health condition* |  |  |  |  |  |
| Sexual minority # Yes | 1·01 | 0·90 | 1·12 | 0·91 |  |
|  |  |  |  |  |  |
| *Ethnicity* |  |  |  |  |  |
| Mixed/Multiple ethnic groups | 1·14 | 1·04 | 1·25 | 0·01 |  |
| Asian/Asian British | 1·35 | 1·30 | 1·41 | 0·00 |  |
| Black/African/Caribbean/Black British | 0·69 | 0·65 | 0·74 | 0·00 |  |
| Other ethnic group | 1·38 | 1·27 | 1·50 | 0·00 |  |
|  |  |  |  |  |  |
| *Interaction between sexual orientation and ethnicity* |  |  |  |  |  |
| Sexual minority#Mixed/Multiple ethnic groups | 0·63 | 0·47 | 0·84 | 0·00 |  |
| Sexual minority#Asian/Asian British | 0·71 | 0·57 | 0·89 | 0·00 |  |
| Sexual minority#Black/African/Caribbean/Black British | 1·11 | 0·80 | 1·55 | 0·52 |  |
| Sexual minority#Other ethnic group | 1·09 | 0·81 | 1·46 | 0·58 |  |
|  |  |  |  |  |  |
| *Interaction between self-reported mental health condition and ethnicity* |  |  |  |  |  |
| Yes#Mixed/Multiple ethnic groups | 1·05 | 0·85 | 1·29 | 0·68 |  |
| Yes#Asian/Asian British | 1·12 | 1·00 | 1·26 | 0·06 |  |
| Yes#Black/African/Caribbean/Black British | 1·31 | 1·08 | 1·59 | 0·01 |  |
| Yes#Other ethnic group | 0·86 | 0·68 | 1·08 | 0·20 |  |
|  |  |  |  |  |  |
| *Three-way interaction between sexual orientation, self-reported mental health condition and ethnicity* |  |  |  |  |  |
| Sexual minority#Yes#Mixed/Multiple ethnic groups | 1·31 | 0·82 | 2·09 | 0·26 |  |
| Sexual minority#Yes#Asian/Asian British | 0·78 | 0·50 | 1·23 | 0·29 |  |
| Sexual minority#Yes#Black/African/Caribbean/Black British | 0·53 | 0·27 | 1·06 | 0·07 |  |
| Sexual minority#Yes#Other ethnic group | 0·91 | 0·51 | 1·62 | 0·74 |  |
|  |  |  |  |  |  |
| *Sex* |  |  |  |  |  |
| Male | 0·95 | 0·93 | 0·98 | 0·00 |  |
| **Table S26 (continued)** |  |  |  |  |  |
|  | Odds Ratio | 95% CI L | 95% CI U | p-value |  |
| *Sex* |  |  |  |  |  |
| Non-binary | 0·95 | 0·77 | 1·18 | 0·65 |  |
| Prefer to self-describe | 2·02 | 1·60 | 2·56 | 0·00 |  |
| Prefer not to say | 1·31 | 1·12 | 1·53 | 0·00 |  |
|  |  |  |  |  |  |
| *Gender identity* |  |  |  |  |  |
| Transgender | 1·12 | 0·96 | 1·32 | 0·16 |  |
| Prefer not to say | 1·26 | 1·06 | 1·49 | 0·01 |  |
|  |  |  |  |  |  |
| *Age* |  |  |  |  |  |
| 25-34 | 0·93 | 0·89 | 0·98 | 0·00 |  |
| 35-44 | 0·74 | 0·71 | 0·78 | 0·00 |  |
| 45-54 | 0·55 | 0·52 | 0·57 | 0·00 |  |
| 55-64 | 0·39 | 0·37 | 0·41 | 0·00 |  |
| 65-74 | 0·26 | 0·25 | 0·27 | 0·00 |  |
| 75+ | 0·19 | 0·18 | 0·20 | 0·00 |  |
|  |  |  |  |  |  |
| *Multimorbidity* |  |  |  |  |  |
| No multimorbidity | 1·29 | 1·26 | 1·33 | 0·00 |  |
| Multimorbidity | 1·73 | 1·67 | 1·79 | 0·00 |  |
|  |  |  |  |  |  |
| *Patient IMD quintile* |  |  |  |  |  |
| Q2 | 0·91 | 0·88 | 0·94 | 0·00 |  |
| Q3 | 0·80 | 0·77 | 0·83 | 0·00 |  |
| Q4 | 0·75 | 0·72 | 0·78 | 0·00 |  |
| Q5 (least deprived) | 0·71 | 0·68 | 0·73 | 0·00 |  |
|  |  |  |  |  |  |
| *Region* |  |  |  |  |  |
| London | 1·19 | 1·16 | 1·23 | 0·00 |  |
|  |  |  |  |  |  |
| *Survey year* |  |  |  |  |  |
| 2023 | 0·98 | 0·96 | 1·01 | 0·13 |  |
|  |  |  |  |  |  |
| Observations | 1 088 059 |  |  |  |  |

| **Table S27** |  |  |  |  |  |
| --- | --- | --- | --- | --- | --- |
| *Fully adjusted logistic regression model assessing the interaction between sexual orientation, self-reported long-term mental health condition and ethnicity on perceived involvement in care and treatment Decisions* | | | | |  |
|  |  |  |  |  |  |
|  | Odds Ratio | 95% CI L | 95% CI U | p-value |  |
| *Sexual orientation* |  |  |  |  |  |
| Sexual minority | 0·90 | 0·84 | 0·97 | 0·00 |  |
|  |  |  |  |  |  |
| *Self-reported mental health condition* |  |  |  |  |  |
| Yes | 0·87 | 0·84 | 0·90 | 0·00 |  |
|  |  |  |  |  |  |
| *Interaction between sexual orientation and self-reported mental health condition* |  |  |  |  |  |
| Sexual minority # Yes | 1·08 | 0·97 | 1·20 | 0·18 |  |
|  |  |  |  |  |  |
| *Ethnicity* |  |  |  |  |  |
| Mixed/Multiple ethnic groups | 0·82 | 0·75 | 0·90 | 0·00 |  |
| Asian/Asian British | 0·70 | 0·67 | 0·72 | 0·00 |  |
| Black/African/Caribbean/Black British | 0·92 | 0·87 | 0·97 | 0·00 |  |
| Other ethnic group | 0·59 | 0·55 | 0·64 | 0·00 |  |
|  |  |  |  |  |  |
| *Interaction between sexual orientation and ethnicity* |  |  |  |  |  |
| Sexual minority#Mixed/Multiple ethnic groups | 1·21 | 0·89 | 1·66 | 0·23 |  |
| Sexual minority#Asian/Asian British | 1·06 | 0·88 | 1·27 | 0·55 |  |
| Sexual minority#Black/African/Caribbean/Black British | 0·68 | 0·54 | 0·87 | 0·00 |  |
| Sexual minority#Other ethnic group | 0·98 | 0·77 | 1·23 | 0·84 |  |
|  |  |  |  |  |  |
| *Interaction between self-reported mental health condition and ethnicity* |  |  |  |  |  |
| Yes#Mixed/Multiple ethnic groups | 1·02 | 0·83 | 1·25 | 0·83 |  |
| Yes#Asian/Asian British | 0·99 | 0·88 | 1·11 | 0·80 |  |
| Yes#Black/African/Caribbean/Black British | 1·18 | 0·98 | 1·41 | 0·08 |  |
| Yes#Other ethnic group | 1·21 | 0·98 | 1·50 | 0·07 |  |
|  |  |  |  |  |  |
| *Three-way interaction between sexual orientation, self-reported mental health condition and ethnicity* |  |  |  |  |  |
| Sexual minority#Yes#Mixed/Multiple ethnic groups | 0·77 | 0·47 | 1·26 | 0·29 |  |
| Sexual minority#Yes#Asian/Asian British | 1·60 | 0·99 | 2·58 | 0·06 |  |
| Sexual minority#Yes#Black/African/Caribbean/Black British | 1·47 | 0·81 | 2·69 | 0·21 |  |
| Sexual minority#Yes#Other ethnic group | 0·94 | 0·52 | 1·68 | 0·83 |  |
|  |  |  |  |  |  |
| *Sex* |  |  |  |  |  |
| Male | 0·88 | 0·86 | 0·90 | 0·00 |  |
| **Table S27 (continued)** |  |  |  |  |  |
|  | Odds Ratio | 95% CI L | 95% CI U | p-value |  |
| *Sex* |  |  |  |  |  |
| Non-binary | 0·93 | 0·75 | 1·15 | 0·50 |  |
| Prefer to self-describe | 0·60 | 0·48 | 0·76 | 0·00 |  |
| Prefer not to say | 0·68 | 0·58 | 0·79 | 0·00 |  |
|  |  |  |  |  |  |
| *Gender identity* |  |  |  |  |  |
| Transgender | 0·72 | 0·63 | 0·82 | 0·00 |  |
| Prefer not to say | 0·65 | 0·55 | 0·76 | 0·00 |  |
|  |  |  |  |  |  |
| *Age* |  |  |  |  |  |
| 25-34 | 1·00 | 0·95 | 1·05 | 0·93 |  |
| 35-44 | 1·08 | 1·03 | 1·14 | 0·00 |  |
| 45-54 | 1·34 | 1·27 | 1·40 | 0·00 |  |
| 55-64 | 1·69 | 1·61 | 1·77 | 0·00 |  |
| 65-74 | 2·20 | 2·08 | 2·31 | 0·00 |  |
| 75+ | 2·63 | 2·49 | 2·78 | 0·00 |  |
|  |  |  |  |  |  |
| *Multimorbidity* |  |  |  |  |  |
| No multimorbidity | 0·82 | 0·79 | 0·84 | 0·00 |  |
| Multimorbidity | 0·63 | 0·61 | 0·65 | 0·00 |  |
|  |  |  |  |  |  |
| *Patient IMD quintile* |  |  |  |  |  |
| Q2 | 1·16 | 1·13 | 1·20 | 0·00 |  |
| Q3 | 1·33 | 1·29 | 1·38 | 0·00 |  |
| Q4 | 1·42 | 1·38 | 1·47 | 0·00 |  |
| Q5 (least deprived) | 1·58 | 1·53 | 1·64 | 0·00 |  |
|  |  |  |  |  |  |
| *Region* |  |  |  |  |  |
| London | 0·89 | 0·87 | 0·92 | 0·00 |  |
|  |  |  |  |  |  |
| *Survey year* |  |  |  |  |  |
| 2023 | 1·05 | 1·03 | 1·08 | 0·00 |  |
|  |  |  |  |  |  |
| Observations | 1 019 397 |  |  |  |  |

| **Table S28** |  |  |  |  |  |
| --- | --- | --- | --- | --- | --- |
| *Fully adjusted logistic regression model assessing the interaction between sexual orientation, self-reported long-term mental health condition and ethnicity on needs met* | | | | |  |
|  |  |  |  |  |  |
|  | Odds Ratio | 95% CI L | 95% CI U | p-value |  |
| *Sexual orientation* |  |  |  |  |  |
| Sexual minority | 0·92 | 0·86 | 0·98 | 0·01 |  |
|  |  |  |  |  |  |
| *Self-reported mental health condition* |  |  |  |  |  |
| Yes | 0·83 | 0·80 | 0·86 | 0·00 |  |
|  |  |  |  |  |  |
| *Interaction between sexual orientation and self-reported mental health condition* |  |  |  |  |  |
| Sexual minority # Yes | 1·09 | 0·98 | 1·21 | 0·13 |  |
|  |  |  |  |  |  |
| *Ethnicity* |  |  |  |  |  |
| Mixed/Multiple ethnic groups | 0·84 | 0·78 | 0·92 | 0·00 |  |
| Asian/Asian British | 0·72 | 0·69 | 0·75 | 0·00 |  |
| Black/African/Caribbean/Black British | 0·95 | 0·89 | 1·00 | 0·07 |  |
| Other ethnic group | 0·63 | 0·58 | 0·68 | 0·00 |  |
|  |  |  |  |  |  |
| *Interaction between sexual orientation and ethnicity* |  |  |  |  |  |
| Sexual minority#Mixed/Multiple ethnic groups | 1·15 | 0·88 | 1·51 | 0·30 |  |
| Sexual minority#Asian/Asian British | 1·03 | 0·85 | 1·24 | 0·78 |  |
| Sexual minority#Black/African/Caribbean/Black British | 0·75 | 0·59 | 0·95 | 0·02 |  |
| Sexual minority#Other ethnic group | 1·13 | 0·87 | 1·47 | 0·37 |  |
|  |  |  |  |  |  |
| *Interaction between self-reported mental health condition and ethnicity* |  |  |  |  |  |
| Yes#Mixed/Multiple ethnic groups | 0·98 | 0·79 | 1·21 | 0·85 |  |
| Yes#Asian/Asian British | 1·01 | 0·90 | 1·13 | 0·90 |  |
| Yes#Black/African/Caribbean/Black British | 0·93 | 0·78 | 1·10 | 0·40 |  |
| Yes#Other ethnic group | 1·05 | 0·83 | 1·33 | 0·67 |  |
|  |  |  |  |  |  |
| *Three-way interaction between sexual orientation, self-reported mental health condition and ethnicity* |  |  |  |  |  |
| Sexual minority#Yes#Mixed/Multiple ethnic groups | 0·92 | 0·58 | 1·45 | 0·72 |  |
| Sexual minority#Yes#Asian/Asian British | 1·42 | 0·89 | 2·26 | 0·14 |  |
| Sexual minority#Yes#Black/African/Caribbean/Black British | 2·57 | 1·32 | 5·00 | 0·01 |  |
| Sexual minority#Yes#Other ethnic group | 1·03 | 0·58 | 1·83 | 0·91 |  |
|  |  |  |  |  |  |
| *Sex* |  |  |  |  |  |
| Male | 0·92 | 0·90 | 0·94 | 0·00 |  |
| **Table S28 (continued)** |  |  |  |  |  |
|  | Odds Ratio | 95% CI L | 95% CI U | p-value |  |
| *Sex* |  |  |  |  |  |
| Non-binary | 0·93 | 0·75 | 1·14 | 0·47 |  |
| Prefer to self-describe | 0·48 | 0·38 | 0·60 | 0·00 |  |
| Prefer not to say | 0·69 | 0·59 | 0·80 | 0·00 |  |
|  |  |  |  |  |  |
| *Gender identity* |  |  |  |  |  |
| Transgender | 0·89 | 0·77 | 1·02 | 0·10 |  |
| Prefer not to say | 0·76 | 0·65 | 0·90 | 0·00 |  |
|  |  |  |  |  |  |
| *Age* |  |  |  |  |  |
| 25-34 | 1·03 | 0·98 | 1·08 | 0·19 |  |
| 35-44 | 1·17 | 1·12 | 1·22 | 0·00 |  |
| 45-54 | 1·57 | 1·50 | 1·65 | 0·00 |  |
| 55-64 | 2·20 | 2·10 | 2·30 | 0·00 |  |
| 65-74 | 3·25 | 3·09 | 3·42 | 0·00 |  |
| 75+ | 3·99 | 3·78 | 4·22 | 0·00 |  |
|  |  |  |  |  |  |
| *Multimorbidity* |  |  |  |  |  |
| No multimorbidity | 0·80 | 0·78 | 0·82 | 0·00 |  |
| Multimorbidity | 0·62 | 0·60 | 0·64 | 0·00 |  |
|  |  |  |  |  |  |
| *Patient IMD quintile* |  |  |  |  |  |
| Q2 | 1·15 | 1·12 | 1·19 | 0·00 |  |
| Q3 | 1·27 | 1·23 | 1·32 | 0·00 |  |
| Q4 | 1·37 | 1·33 | 1·42 | 0·00 |  |
| Q5 (least deprived) | 1·48 | 1·43 | 1·53 | 0·00 |  |
|  |  |  |  |  |  |
| *Region* |  |  |  |  |  |
| London | 0·89 | 0·87 | 0·92 | 0·00 |  |
|  |  |  |  |  |  |
| *Survey year* |  |  |  |  |  |
| 2023 | 1·01 | 0·99 | 1·03 | 0·24 |  |
|  |  |  |  |  |  |
| Observations | 1 129 055 |  |  |  |  |

| **Table S29** |  |  |  |  |  |
| --- | --- | --- | --- | --- | --- |
| *Fully adjusted logistic regression model assessing the interaction between sexual orientation, self-reported long-term mental health condition and deprivation level on help-seeking behaviour* | | | | |  |
|  |  |  |  |  |  |
|  | Odds Ratio | 95% CI L | 95% CI U | p-value |  |
| *Sexual orientation* |  |  |  |  |  |
| Sexual minority | 1·15 | 1·07 | 1·24 | 0·00 |  |
|  |  |  |  |  |  |
| *Self-reported mental health condition* |  |  |  |  |  |
| Yes | 1·02 | 0·98 | 1·06 | 0·31 |  |
|  |  |  |  |  |  |
| *Interaction between sexual orientation and self-reported mental health condition* |  |  |  |  |  |
| Sexual minority#Yes | 0·93 | 0·81 | 1·06 | 0·29 |  |
|  |  |  |  |  |  |
| *Patient IMD quintile* |  |  |  |  |  |
| Q2 | 1·02 | 1·00 | 1·04 | 0·09 |  |
| Q3 | 1·03 | 1·01 | 1·05 | 0·00 |  |
| Q4 | 1·06 | 1·04 | 1·08 | 0·00 |  |
| Q5 (least deprived) | 1·10 | 1·08 | 1·12 | 0·00 |  |
|  |  |  |  |  |  |
| *Interaction between sexual orientation and deprivation level* |  |  |  |  |  |
| Sexual minority#Q2 | 1·07 | 0·97 | 1·18 | 0·19 |  |
| Sexual minority#Q3 | 1·01 | 0·91 | 1·12 | 0·91 |  |
| Sexual minority#Q4 | 1·08 | 0·97 | 1·20 | 0·16 |  |
| Sexual minority#Q5 (least deprived) | 1·02 | 0·91 | 1·14 | 0·78 |  |
|  |  |  |  |  |  |
| *Interaction between mental health condition and deprivation level* |  |  |  |  |  |
| Yes#Q2 | 1·08 | 1·02 | 1·15 | 0·01 |  |
| Yes#Q3 | 1·10 | 1·03 | 1·16 | 0·00 |  |
| Yes#Q4 | 1·14 | 1·07 | 1·21 | 0·00 |  |
| Yes#Q5 (least deprived) | 1·05 | 0·98 | 1·11 | 0·15 |  |
|  |  |  |  |  |  |
| *Three-way interaction between sexual orientation, self-reported mental health condition and deprivation* |  |  |  |  |  |
| Sexual minority#Yes#Q2 | 1·07 | 0·88 | 1·31 | 0·48 |  |
| Sexual minority#Yes#Q3 | 1·17 | 0·95 | 1·45 | 0·13 |  |
| Sexual minority#Yes#Q4 | 0·90 | 0·72 | 1·13 | 0·36 |  |
| Sexual minority#Yes#Q5 (least deprived) | 0·96 | 0·75 | 1·22 | 0·71 |  |
|  |  |  |  |  |  |
| *Sex* |  |  |  |  |  |
| Male | 0·82 | 0·81 | 0·83 | 0·00 |  |
| **Table S29 (continued)** |  |  |  |  |  |
|  | Odds Ratio | 95% CI L | 95% CI U | p-value |  |
| *Sex* |  |  |  |  |  |
| Non-binary | 1·00 | 0·85 | 1·16 | 0·96 |  |
| Prefer to self-describe | 0·85 | 0·71 | 1·02 | 0·08 |  |
| Prefer not to say | 0·90 | 0·81 | 0·99 | 0·04 |  |
|  |  |  |  |  |  |
| *Gender identity* |  |  |  |  |  |
| Transgender | 0·91 | 0·82 | 1·01 | 0·07 |  |
| Prefer not to say | 0·92 | 0·81 | 1·03 | 0·16 |  |
|  |  |  |  |  |  |
| *Age* |  |  |  |  |  |
| 25-34 | 0·96 | 0·93 | 0·99 | 0·02 |  |
| 35-44 | 0·82 | 0·79 | 0·85 | 0·00 |  |
| 45-54 | 0·57 | 0·55 | 0·59 | 0·00 |  |
| 55-64 | 0·42 | 0·41 | 0·44 | 0·00 |  |
| 65-74 | 0·34 | 0·33 | 0·35 | 0·00 |  |
| 75+ | 0·28 | 0·27 | 0·29 | 0·00 |  |
|  |  |  |  |  |  |
| *Multimorbidity* |  |  |  |  |  |
| No multimorbidity | 1·03 | 1·02 | 1·05 | 0·00 |  |
| Multimorbidity | 1·20 | 1·18 | 1·22 | 0·00 |  |
|  |  |  |  |  |  |
| *Ethnicity* |  |  |  |  |  |
| Mixed/Multiple ethnic groups | 1·15 | 1·10 | 1·21 | 0·00 |  |
| Asian/Asian British | 1·36 | 1·33 | 1·39 | 0·00 |  |
| Black/African/Caribbean/Black British | 0·95 | 0·93 | 0·99 | 0·00 |  |
| Other ethnic group | 1·17 | 1·12 | 1·23 | 0·00 |  |
|  |  |  |  |  |  |
| *Region* |  |  |  |  |  |
| London | 1·07 | 1·05 | 1·09 | 0·00 |  |
|  |  |  |  |  |  |
| *Survey year* |  |  |  |  |  |
| 2023 | 1·07 | 1·06 | 1·08 | 0·00 |  |
|  |  |  |  |  |  |
| Observations | 1 138 852 |  |  |  |  |

| **Table S30** |  |  |  |  |  |
| --- | --- | --- | --- | --- | --- |
| *Fully adjusted logistic regression model assessing the interaction between sexual orientation, self-reported long-term mental health condition and deprivation level on mental health needs recognised by the HCP* | | | | |  |
|  |  |  |  |  |  |
|  | Odds Ratio | 95% CI L | 95% CI U | p-value |  |
| *Sexual orientation* |  |  |  |  |  |
| Sexual minority | 0·88 | 0·79 | 0·99 | 0·04 |  |
|  |  |  |  |  |  |
| *Self-reported mental health condition* |  |  |  |  |  |
| Yes | 0·98 | 0·93 | 1·03 | 0·43 |  |
|  |  |  |  |  |  |
| *Interaction between sexual orientation and self-reported mental health condition* |  |  |  |  |  |
| Sexual minority#Yes | 1·06 | 0·89 | 1·26 | 0·52 |  |
|  |  |  |  |  |  |
| *Patient IMD quintile* |  |  |  |  |  |
| Q2 | 1·07 | 1·03 | 1·11 | 0·00 |  |
| Q3 | 1·20 | 1·15 | 1·25 | 0·00 |  |
| Q4 | 1·28 | 1·23 | 1·33 | 0·00 |  |
| Q5 (least deprived) | 1·35 | 1·30 | 1·41 | 0·00 |  |
|  |  |  |  |  |  |
| *Interaction between sexual orientation and deprivation level* |  |  |  |  |  |
| Sexual minority#Q2 | 1·02 | 0·87 | 1·19 | 0·79 |  |
| Sexual minority#Q3 | 0·95 | 0·80 | 1·13 | 0·55 |  |
| Sexual minority#Q4 | 0·85 | 0·71 | 1·02 | 0·08 |  |
| Sexual minority#Q5 (least deprived) | 0·89 | 0·73 | 1·10 | 0·28 |  |
|  |  |  |  |  |  |
| *Interaction between mental health condition and deprivation level* |  |  |  |  |  |
| Yes#Q2 | 1·01 | 0·93 | 1·09 | 0·89 |  |
| Yes#Q3 | 0·99 | 0·91 | 1·07 | 0·78 |  |
| Yes#Q4 | 0·94 | 0·86 | 1·02 | 0·12 |  |
| Yes#Q5 (least deprived) | 0·94 | 0·86 | 1·02 | 0·14 |  |
|  |  |  |  |  |  |
| *Three-way interaction between sexual orientation, self-reported mental health condition and deprivation* |  |  |  |  |  |
| Sexual minority#Yes#Q2 | 1·07 | 0·84 | 1·37 | 0·59 |  |
| Sexual minority#Yes#Q3 | 1·01 | 0·76 | 1·35 | 0·93 |  |
| Sexual minority#Yes#Q4 | 1·29 | 0·96 | 1·72 | 0·09 |  |
| Sexual minority#Yes#Q5 (least deprived) | 1·26 | 0·92 | 1·72 | 0·14 |  |
|  |  |  |  |  |  |
| *Sex* |  |  |  |  |  |
| Male | 1·06 | 1·03 | 1·08 | 0·00 |  |
| **Table S30 (continued)** |  |  |  |  |  |
|  | Odds Ratio | 95% CI L | 95% CI U | p-value |  |
| *Sex* |  |  |  |  |  |
| Non-binary | 0·90 | 0·74 | 1·11 | 0·33 |  |
| Prefer to self-describe | 0·56 | 0·44 | 0·71 | 0·00 |  |
| Prefer not to say | 0·83 | 0·71 | 0·97 | 0·02 |  |
|  |  |  |  |  |  |
| *Gender identity* |  |  |  |  |  |
| Transgender | 0·84 | 0·74 | 0·97 | 0·02 |  |
| Prefer not to say | 0·77 | 0·64 | 0·93 | 0·01 |  |
|  |  |  |  |  |  |
| *Age* |  |  |  |  |  |
| 25-34 | 1·02 | 0·97 | 1·08 | 0·42 |  |
| 35-44 | 1·09 | 1·04 | 1·14 | 0·00 |  |
| 45-54 | 1·28 | 1·22 | 1·35 | 0·00 |  |
| 55-64 | 1·54 | 1·46 | 1·61 | 0·00 |  |
| 65-74 | 1·84 | 1·74 | 1·94 | 0·00 |  |
| 75+ | 2·26 | 2·14 | 2·39 | 0·00 |  |
|  |  |  |  |  |  |
| *Multimorbidity* |  |  |  |  |  |
| No multimorbidity | 0·80 | 0·78 | 0·83 | 0·00 |  |
| Multimorbidity | 0·60 | 0·58 | 0·62 | 0·00 |  |
|  |  |  |  |  |  |
| *Ethnicity* |  |  |  |  |  |
| Mixed/Multiple ethnic groups | 0·83 | 0·77 | 0·89 | 0·00 |  |
| Asian/Asian British | 0·66 | 0·64 | 0·69 | 0·00 |  |
| Black/African/Caribbean/Black British | 0·81 | 0·76 | 0·86 | 0·00 |  |
| Other ethnic group | 0·71 | 0·66 | 0·77 | 0·00 |  |
|  |  |  |  |  |  |
| *Region* |  |  |  |  |  |
| London | 0·90 | 0·87 | 0·93 | 0·00 |  |
|  |  |  |  |  |  |
| *Survey year* |  |  |  |  |  |
| 2023 | 1·06 | 1·04 | 1·08 | 0·00 |  |
|  |  |  |  |  |  |
| Observations | 500 618 |  |  |  |  |

| **Table S31** |  |  |  |  |  |
| --- | --- | --- | --- | --- | --- |
| *Fully adjusted logistic regression model assessing the interaction between sexual orientation, self-reported long-term mental health condition and deprivation level on confidence & trust in the HCP* | | | | |  |
|  |  |  |  |  |  |
|  | Odds Ratio | 95% CI L | 95% CI U | p-value |  |
| *Sexual orientation* |  |  |  |  |  |
| Sexual minority | 0·98 | 0·87 | 1·11 | 0·76 |  |
|  |  |  |  |  |  |
| *Self-reported mental health condition* |  |  |  |  |  |
| Yes | 0·83 | 0·78 | 0·88 | 0·00 |  |
|  |  |  |  |  |  |
| *Interaction between sexual orientation and self-reported mental health condition* |  |  |  |  |  |
| Sexual minority#Yes | 1·15 | 0·94 | 1·39 | 0·17 |  |
|  |  |  |  |  |  |
| *Patient IMD quintile* |  |  |  |  |  |
| Q2 | 1·16 | 1·12 | 1·21 | 0·00 |  |
| Q3 | 1·41 | 1·36 | 1·48 | 0·00 |  |
| Q4 | 1·53 | 1·47 | 1·60 | 0·00 |  |
| Q5 (least deprived) | 1·68 | 1·61 | 1·76 | 0·00 |  |
|  |  |  |  |  |  |
| *Interaction between sexual orientation and deprivation level* |  |  |  |  |  |
| Sexual minority#Q2 | 1·02 | 0·86 | 1·21 | 0·85 |  |
| Sexual minority#Q3 | 0·83 | 0·68 | 1·01 | 0·06 |  |
| Sexual minority#Q4 | 0·91 | 0·75 | 1·11 | 0·37 |  |
| Sexual minority#Q5 (least deprived) | 0·80 | 0·64 | 1·00 | 0·05 |  |
|  |  |  |  |  |  |
| *Interaction between mental health condition and deprivation level* |  |  |  |  |  |
| Yes#Q2 | 1·02 | 0·93 | 1·12 | 0·70 |  |
| Yes#Q3 | 0·93 | 0·84 | 1·02 | 0·13 |  |
| Yes#Q4 | 0·90 | 0·81 | 0·99 | 0·04 |  |
| Yes#Q5 (least deprived) | 0·89 | 0·80 | 1·00 | 0·04 |  |
|  |  |  |  |  |  |
| *Three-way interaction between sexual orientation, self-reported mental health condition and deprivation* |  |  |  |  |  |
| Sexual minority#Yes#Q2 | 0·88 | 0·67 | 1·17 | 0·39 |  |
| Sexual minority#Yes#Q3 | 1·10 | 0·80 | 1·53 | 0·55 |  |
| Sexual minority#Yes#Q4 | 0·95 | 0·68 | 1·33 | 0·78 |  |
| Sexual minority#Yes#Q5 (least deprived) | 1·07 | 0·74 | 1·53 | 0·72 |  |
|  |  |  |  |  |  |
| *Sex* |  |  |  |  |  |
| Male | 1·04 | 1·01 | 1·06 | 0·00 |  |
| **Table S31 (continued)** |  |  |  |  |  |
|  | Odds Ratio | 95% CI L | 95% CI U | p-value |  |
| *Sex* |  |  |  |  |  |
| Non-binary | 1·09 | 0·87 | 1·37 | 0·44 |  |
| Prefer to self-describe | 0·46 | 0·36 | 0·59 | 0·00 |  |
| Prefer not to say | 0·73 | 0·61 | 0·86 | 0·00 |  |
|  |  |  |  |  |  |
| *Gender identity* |  |  |  |  |  |
| Transgender | 0·66 | 0·57 | 0·77 | 0·00 |  |
| Prefer not to say | 0·70 | 0·59 | 0·83 | 0·00 |  |
|  |  |  |  |  |  |
| *Age* |  |  |  |  |  |
| 25-34 | 0·90 | 0·86 | 0·95 | 0·00 |  |
| 35-44 | 1·05 | 1·00 | 1·11 | 0·06 |  |
| 45-54 | 1·37 | 1·30 | 1·45 | 0·00 |  |
| 55-64 | 1·93 | 1·83 | 2·04 | 0·00 |  |
| 65-74 | 2·79 | 2·64 | 2·96 | 0·00 |  |
| 75+ | 3·64 | 3·42 | 3·88 | 0·00 |  |
|  |  |  |  |  |  |
| *Multimorbidity* |  |  |  |  |  |
| No multimorbidity | 0·77 | 0·74 | 0·79 | 0·00 |  |
| Multimorbidity | 0·57 | 0·55 | 0·59 | 0·00 |  |
|  |  |  |  |  |  |
| *Ethnicity* |  |  |  |  |  |
| Mixed/Multiple ethnic groups | 0·87 | 0·81 | 0·95 | 0·00 |  |
| Asian/Asian British | 0·75 | 0·72 | 0·78 | 0·00 |  |
| Black/African/Caribbean/Black British | 1·23 | 1·16 | 1·32 | 0·00 |  |
| Other ethnic group | 0·69 | 0·64 | 0·75 | 0·00 |  |
|  |  |  |  |  |  |
| *Region* |  |  |  |  |  |
| London | 0·87 | 0·84 | 0·90 | 0·00 |  |
|  |  |  |  |  |  |
| *Survey year* |  |  |  |  |  |
| 2023 | 1·00 | 0·98 | 1·03 | 0·76 |  |
|  |  |  |  |  |  |
| Observations | 1 125 400 |  |  |  |  |

| **Table S32** |  |  |  |  |  |
| --- | --- | --- | --- | --- | --- |
| *Fully adjusted logistic regression model assessing the interaction between sexual orientation, self-reported long-term mental health condition and deprivation level on perceived negative HCP interpersonal skills* | | | | |  |
|  |  |  |  |  |  |
|  | Odds Ratio | 95% CI L | 95% CI U | p-value |  |
| *Sexual orientation* |  |  |  |  |  |
| Sexual minority | 1·05 | 0·93 | 1·18 | 0·42 |  |
|  |  |  |  |  |  |
| *Self-reported mental health condition* |  |  |  |  |  |
| Yes | 1·27 | 1·19 | 1·35 | 0·00 |  |
|  |  |  |  |  |  |
| *Interaction between sexual orientation and self-reported mental health condition* |  |  |  |  |  |
| Sexual minority#Yes | 1·07 | 0·89 | 1·29 | 0·47 |  |
|  |  |  |  |  |  |
| *Patient IMD quintile* |  |  |  |  |  |
| Q2 | 0·92 | 0·89 | 0·96 | 0·00 |  |
| Q3 | 0·79 | 0·76 | 0·82 | 0·00 |  |
| Q4 | 0·74 | 0·71 | 0·78 | 0·00 |  |
| Q5 (least deprived) | 0·69 | 0·66 | 0·72 | 0·00 |  |
|  |  |  |  |  |  |
| *Interaction between sexual orientation and deprivation level* |  |  |  |  |  |
| Sexual minority#Q2 | 0·91 | 0·77 | 1·07 | 0·25 |  |
| Sexual minority#Q3 | 1·17 | 0·96 | 1·42 | 0·12 |  |
| Sexual minority#Q4 | 1·06 | 0·88 | 1·27 | 0·56 |  |
| Sexual minority#Q5 (least deprived) | 1·31 | 1·07 | 1·61 | 0·01 |  |
|  |  |  |  |  |  |
| *Interaction between mental health condition and deprivation level* |  |  |  |  |  |
| Yes#Q2 | 0·96 | 0·88 | 1·05 | 0·36 |  |
| Yes#Q3 | 1·04 | 0·95 | 1·15 | 0·39 |  |
| Yes#Q4 | 1·04 | 0·94 | 1·15 | 0·45 |  |
| Yes#Q5 (least deprived) | 1·01 | 0·91 | 1·12 | 0·85 |  |
|  |  |  |  |  |  |
| *Three-way interaction between sexual orientation, self-reported mental health condition and deprivation* |  |  |  |  |  |
| Sexual minority#Yes#Q2 | 1·04 | 0·80 | 1·35 | 0·79 |  |
| Sexual minority#Yes#Q3 | 0·85 | 0·63 | 1·16 | 0·31 |  |
| Sexual minority#Yes#Q4 | 0·89 | 0·65 | 1·22 | 0·47 |  |
| Sexual minority#Yes#Q5 (least deprived) | 0·86 | 0·62 | 1·19 | 0·35 |  |
|  |  |  |  |  |  |
| *Sex* |  |  |  |  |  |
| Male | 0·95 | 0·93 | 0·98 | 0·00 |  |
| **Table S32 (continued)** |  |  |  |  |  |
|  | Odds Ratio | 95% CI L | 95% CI U | p-value |  |
| *Sex* |  |  |  |  |  |
| Non-binary | 0·95 | 0·77 | 1·18 | 0·65 |  |
| Prefer to self-describe | 2·03 | 1·60 | 2·56 | 0·00 |  |
| Prefer not to say | 1·30 | 1·11 | 1·53 | 0·00 |  |
|  |  |  |  |  |  |
| *Gender identity* |  |  |  |  |  |
| Transgender | 1·12 | 0·96 | 1·32 | 0·16 |  |
| Prefer not to say | 1·25 | 1·06 | 1·48 | 0·01 |  |
|  |  |  |  |  |  |
| *Age* |  |  |  |  |  |
| 25-34 | 0·93 | 0·89 | 0·98 | 0·00 |  |
| 35-44 | 0·74 | 0·71 | 0·78 | 0·00 |  |
| 45-54 | 0·55 | 0·52 | 0·57 | 0·00 |  |
| 55-64 | 0·39 | 0·37 | 0·41 | 0·00 |  |
| 65-74 | 0·26 | 0·25 | 0·27 | 0·00 |  |
| 75+ | 0·19 | 0·18 | 0·20 | 0·00 |  |
|  |  |  |  |  |  |
| *Multimorbidity* |  |  |  |  |  |
| No multimorbidity | 1·29 | 1·26 | 1·33 | 0·00 |  |
| Multimorbidity | 1·73 | 1·67 | 1·79 | 0·00 |  |
|  |  |  |  |  |  |
| *Ethnicity* |  |  |  |  |  |
| Mixed/Multiple ethnic groups | 1·10 | 1·02 | 1·19 | 0·02 |  |
| Asian/Asian British | 1·34 | 1·29 | 1·39 | 0·00 |  |
| Black/African/Caribbean/Black British | 0·71 | 0·67 | 0·76 | 0·00 |  |
| Other ethnic group | 1·37 | 1·27 | 1·48 | 0·00 |  |
|  |  |  |  |  |  |
| *Region* |  |  |  |  |  |
| London | 1·19 | 1·16 | 1·23 | 0·00 |  |
|  |  |  |  |  |  |
| *Survey year* |  |  |  |  |  |
| 2023 | 0·98 | 0·96 | 1·00 | 0·12 |  |
|  |  |  |  |  |  |
| Observations | 1 088 059 |  |  |  |  |

| **Table S33** |  |  |  |  |  |
| --- | --- | --- | --- | --- | --- |
| *Fully adjusted logistic regression model assessing the interaction between sexual orientation, self-reported long-term mental health condition and deprivation level on perceive involvement in care and treatment decisions* | | | | |  |
|  |  |  |  |  |  |
|  | Odds Ratio | 95% CI L | 95% CI U | p-value |  |
| *Sexual orientation* |  |  |  |  |  |
| Sexual minority | 0·90 | 0·80 | 1·00 | 0·04 |  |
|  |  |  |  |  |  |
| *Self-reported mental health condition* |  |  |  |  |  |
| Yes | 0·89 | 0·84 | 0·94 | 0·00 |  |
|  |  |  |  |  |  |
| *Interaction between sexual orientation and self-reported mental health condition* |  |  |  |  |  |
| Sexual minority#Yes | 1·13 | 0·94 | 1·36 | 0·20 |  |
|  |  |  |  |  |  |
| *Patient IMD quintile* |  |  |  |  |  |
| Q2 | 1·15 | 1·11 | 1·19 | 0·00 |  |
| Q3 | 1·36 | 1·31 | 1·41 | 0·00 |  |
| Q4 | 1·44 | 1·38 | 1·49 | 0·00 |  |
| Q5 (least deprived) | 1·61 | 1·55 | 1·68 | 0·00 |  |
|  |  |  |  |  |  |
| *Interaction between sexual orientation and deprivation level* |  |  |  |  |  |
| Sexual minority#Q2 | 1·09 | 0·94 | 1·26 | 0·26 |  |
| Sexual minority#Q3 | 0·96 | 0·80 | 1·15 | 0·63 |  |
| Sexual minority#Q4 | 0·99 | 0·83 | 1·18 | 0·93 |  |
| Sexual minority#Q5 (least deprived) | 0·87 | 0·71 | 1·06 | 0·17 |  |
|  |  |  |  |  |  |
| *Interaction between mental health condition and deprivation level* |  |  |  |  |  |
| Yes#Q2 | 1·02 | 0·94 | 1·12 | 0·57 |  |
| Yes#Q3 | 0·95 | 0·87 | 1·04 | 0·26 |  |
| Yes#Q4 | 0·95 | 0·87 | 1·05 | 0·33 |  |
| Yes#Q5 (least deprived) | 0·95 | 0·86 | 1·05 | 0·28 |  |
|  |  |  |  |  |  |
| *Three-way interaction between sexual orientation, self-reported mental health condition and deprivation* |  |  |  |  |  |
| Sexual minority#Yes#Q2 | 0·90 | 0·69 | 1·17 | 0·45 |  |
| Sexual minority#Yes#Q3 | 0·93 | 0·69 | 1·27 | 0·66 |  |
| Sexual minority#Yes#Q4 | 1·06 | 0·78 | 1·44 | 0·70 |  |
| Sexual minority#Yes#Q5 (least deprived) | 1·10 | 0·78 | 1·55 | 0·59 |  |
|  |  |  |  |  |  |
| *Sex* |  |  |  |  |  |
| Male | 0·88 | 0·86 | 0·90 | 0·00 |  |
| **Table S33 (continued)** |  |  |  |  |  |
|  | Odds Ratio | 95% CI L | 95% CI U | p-value |  |
| *Sex* |  |  |  |  |  |
| Non-binary | 0·94 | 0·76 | 1·16 | 0·56 |  |
| Prefer to self-describe | 0·60 | 0·48 | 0·76 | 0·00 |  |
| Prefer not to say | 0·68 | 0·59 | 0·79 | 0·00 |  |
|  |  |  |  |  |  |
| *Gender identity* |  |  |  |  |  |
| Transgender | 0·71 | 0·62 | 0·82 | 0·00 |  |
| Prefer not to say | 0·65 | 0·55 | 0·76 | 0·00 |  |
|  |  |  |  |  |  |
| *Age* |  |  |  |  |  |
| 25-34 | 1·00 | 0·95 | 1·05 | 0·89 |  |
| 35-44 | 1·08 | 1·03 | 1·13 | 0·00 |  |
| 45-54 | 1·33 | 1·27 | 1·40 | 0·00 |  |
| 55-64 | 1·68 | 1·60 | 1·77 | 0·00 |  |
| 65-74 | 2·19 | 2·08 | 2·31 | 0·00 |  |
| 75+ | 2·62 | 2·48 | 2·77 | 0·00 |  |
|  |  |  |  |  |  |
| *Multimorbidity* |  |  |  |  |  |
| No multimorbidity | 0·82 | 0·80 | 0·84 | 0·00 |  |
| Multimorbidity | 0·63 | 0·61 | 0·65 | 0·00 |  |
|  |  |  |  |  |  |
| *Ethnicity* |  |  |  |  |  |
| Mixed/Multiple ethnic groups | 0·84 | 0·78 | 0·90 | 0·00 |  |
| Asian/Asian British | 0·70 | 0·68 | 0·73 | 0·00 |  |
| Black/African/Caribbean/Black British | 0·91 | 0·86 | 0·96 | 0·00 |  |
| Other ethnic group | 0·61 | 0·57 | 0·65 | 0·00 |  |
|  |  |  |  |  |  |
| *Region* |  |  |  |  |  |
| London | 0·89 | 0·87 | 0·92 | 0·00 |  |
|  |  |  |  |  |  |
| *Survey year* |  |  |  |  |  |
| 2023 | 1·05 | 1·03 | 1·08 | 0·00 |  |
|  |  |  |  |  |  |
| Observations | 1 019 397 |  |  |  |  |

| **Table S34** |  |  |  |  |  |
| --- | --- | --- | --- | --- | --- |
| *Fully adjusted logistic regression model assessing the interaction between sexual orientation, self-reported long-term mental health condition and deprivation level on needs met* | | | | |  |
|  |  |  |  |  |  |
|  | Odds Ratio | 95% CI L | 95% CI U | p-value |  |
| *Sexual orientation* |  |  |  |  |  |
| Sexual minority | 0·95 | 0·85 | 1·06 | 0·34 |  |
|  |  |  |  |  |  |
| *Self-reported mental health condition* |  |  |  |  |  |
| Yes | 0·86 | 0·81 | 0·92 | 0·00 |  |
|  |  |  |  |  |  |
| *Interaction between sexual orientation and self-reported mental health condition* |  |  |  |  |  |
| Sexual minority#Yes | 1·15 | 0·96 | 1·38 | 0·13 |  |
|  |  |  |  |  |  |
| *Patient IMD quintile* |  |  |  |  |  |
| Q2 | 1·16 | 1·11 | 1·20 | 0·00 |  |
| Q3 | 1·30 | 1·26 | 1·35 | 0·00 |  |
| Q4 | 1·41 | 1·36 | 1·46 | 0·00 |  |
| Q5 (least deprived) | 1·52 | 1·47 | 1·59 | 0·00 |  |
|  |  |  |  |  |  |
| *Interaction between sexual orientation and deprivation level* |  |  |  |  |  |
| Sexual minority#Q2 | 1·02 | 0·88 | 1·19 | 0·77 |  |
| Sexual minority#Q3 | 0·94 | 0·78 | 1·12 | 0·46 |  |
| Sexual minority#Q4 | 0·94 | 0·79 | 1·12 | 0·49 |  |
| Sexual minority#Q5 (least deprived) | 0·87 | 0·71 | 1·05 | 0·15 |  |
|  |  |  |  |  |  |
| *Interaction between mental health condition and deprivation level* |  |  |  |  |  |
| Yes#Q2 | 1·00 | 0·92 | 1·09 | 0·93 |  |
| Yes#Q3 | 0·92 | 0·84 | 1·01 | 0·07 |  |
| Yes#Q4 | 0·90 | 0·82 | 0·99 | 0·03 |  |
| Yes#Q5 (least deprived) | 0·92 | 0·84 | 1·02 | 0·11 |  |
|  |  |  |  |  |  |
| *Three-way interaction between sexual orientation, self-reported mental health condition and deprivation* |  |  |  |  |  |
| Sexual minority#Yes#Q2 | 0·90 | 0·69 | 1·17 | 0·44 |  |
| Sexual minority#Yes#Q3 | 1·00 | 0·74 | 1·34 | 1·00 |  |
| Sexual minority#Yes#Q4 | 1·03 | 0·76 | 1·40 | 0·85 |  |
| Sexual minority#Yes#Q5 (least deprived) | 0·92 | 0·67 | 1·27 | 0·62 |  |
|  |  |  |  |  |  |
| *Sex* |  |  |  |  |  |
| Male | 0·92 | 0·90 | 0·94 | 0·00 |  |
| **Table S34 (continued)** |  |  |  |  |  |
|  | Odds Ratio | 95% CI L | 95% CI U | p-value |  |
| *Sex* |  |  |  |  |  |
| Non-binary | 0·93 | 0·75 | 1·14 | 0·48 |  |
| Prefer to self-describe | 0·48 | 0·38 | 0·60 | 0·00 |  |
| Prefer not to say | 0·69 | 0·60 | 0·80 | 0·00 |  |
|  |  |  |  |  |  |
| *Gender identity* |  |  |  |  |  |
| Transgender | 0·88 | 0·77 | 1·02 | 0·09 |  |
| Prefer not to say | 0·77 | 0·65 | 0·90 | 0·00 |  |
|  |  |  |  |  |  |
| *Age* |  |  |  |  |  |
| 25-34 | 1·03 | 0·98 | 1·08 | 0·22 |  |
| 35-44 | 1·17 | 1·11 | 1·22 | 0·00 |  |
| 45-54 | 1·57 | 1·49 | 1·64 | 0·00 |  |
| 55-64 | 2·19 | 2·09 | 2·30 | 0·00 |  |
| 65-74 | 3·24 | 3·08 | 3·41 | 0·00 |  |
| 75+ | 3·97 | 3·76 | 4·20 | 0·00 |  |
|  |  |  |  |  |  |
| *Multimorbidity* |  |  |  |  |  |
| No multimorbidity | 0·80 | 0·78 | 0·82 | 0·00 |  |
| Multimorbidity | 0·62 | 0·60 | 0·64 | 0·00 |  |
|  |  |  |  |  |  |
| *Ethnicity* |  |  |  |  |  |
| Mixed/Multiple ethnic groups | 0·85 | 0·79 | 0·92 | 0·00 |  |
| Asian/Asian British | 0·73 | 0·70 | 0·75 | 0·00 |  |
| Black/African/Caribbean/Black British | 0·93 | 0·89 | 0·99 | 0·01 |  |
| Other ethnic group | 0·65 | 0·60 | 0·69 | 0·00 |  |
|  |  |  |  |  |  |
| *Region* |  |  |  |  |  |
| London | 0·89 | 0·87 | 0·92 | 0·00 |  |
|  |  |  |  |  |  |
| *Survey year* |  |  |  |  |  |
| 2023 | 1·01 | 0·99 | 1·03 | 0·24 |  |
|  |  |  |  |  |  |
| Observations | 1 129 055 |  |  |  |  |
